# Long-term inhibition of mutant LRRK2 hyper-kinase activity reduced mouse brain α-synuclein oligomers without adverse effects

**DOI:** 10.1038/s41531-022-00386-9

**Published:** 2022-09-10

**Authors:** Philip Wing-Lok Ho, Eunice Eun-Seo Chang, Chi-Ting Leung, Huifang Liu, Yasine Malki, Shirley Yin-Yu Pang, Zoe Yuen-Kiu Choi, Yingmin Liang, Weng Seng Lai, Yuefei Ruan, Kenneth Mei-Yee Leung, Susan Yung, Judith Choi-Wo Mak, Michelle Hiu-Wai Kung, David B. Ramsden, Shu-Leong Ho

**Affiliations:** 1 Division of Neurology, Department of Medicine, School of Clinical Medicine, University of Hong Kong, Pok Fu Lam, Hong Kong; 2 Division of Respiratory Medicine, Department of Medicine, School of Clinical Medicine, University of Hong Kong, Pok Fu Lam, Hong Kong; 3 Department of Chemistry, State Key Laboratory of Marine Pollution, City University of Hong Kong, Kowloon, Hong Kong; 4 Division of Nephrology, Department of Medicine, School of Clinical Medicine, University of Hong Kong, Pok Fu Lam, Hong Kong; 5 Institute of Metabolism and Systems Research, University of Birmingham, Birmingham, United Kingdom

**Keywords:** Parkinson's disease, Parkinson's disease, Cell biology

## Abstract

Parkinson’s disease (PD) is characterized by dopaminergic neurodegeneration in nigrostriatal and cortical brain regions associated with pathogenic α-synuclein (αSyn) aggregate/oligomer accumulation. LRRK2 hyperactivity is a disease-modifying therapeutic target in PD. However, LRRK2 inhibition may be associated with peripheral effects, albeit with unclear clinical consequences. Here, we significantly reduced αSyn oligomer accumulation in mouse striatum through long-term LRRK2 inhibition using GNE-7915 (specific brain-penetrant LRRK2 inhibitor) without causing adverse peripheral effects. GNE-7915 concentrations in wild-type (WT) mouse sera and brain samples reached a peak at 1 h, which gradually decreased over 24 h following a single subcutaneous (100 mg/kg) injection. The same dose in young WT and LRRK2^R1441G^ mutant mice significantly inhibited LRRK2 kinase activity (Thr73-Rab10 and Ser106-Rab12 phosphorylation) in the lung, which dissipated by 72 h post-injection. 14-month-old mutant mice injected with GNE-7915 twice weekly for 18 weeks (equivalent to ~13 human years) exhibited reduced striatal αSyn oligomer and cortical pSer129-αSyn levels, correlating with inhibition of LRRK2 hyperactivity in brain and lung to WT levels. No GNE-7915-treated mice showed increased mortality or morbidity. Unlike reports of abnormalities in lung and kidney at acute high doses of LRRK2 inhibitors, our GNE-7915-treated mice did not exhibit swollen lamellar bodies in type II pneumocytes or abnormal vacuolation in the kidney. Functional and histopathological assessments of lung, kidney and liver, including whole-body plethysmography, urinary albumin-creatinine ratio (ACR), serum alanine aminotransferase (ALT) and serum interleukin-6 (inflammatory marker) did not reveal abnormalities after long-term GNE-7915 treatment. Long-term inhibition of mutant LRRK2 hyper-kinase activity to physiological levels presents an efficacious and safe disease-modifying therapy to ameliorate synucleinopathy in PD.

## Introduction

Parkinson’s disease (PD) is a progressive, neurodegenerative disorder resulting in poverty of movement and neuropsychiatric features. No treatment exists to halt the progressive neurodegeneration in the brain. All current therapies are palliative. Clinical trials to delay disease progression have been disappointing^1^, partly because there are no reliable biomarkers which correlate to disease progression. Clinical endpoints are too insensitive to demonstrate disease modification, especially when trials are conducted over a relatively short duration.

The pathogenesis of PD involves the interplay of ageing, genetic risks and environmental factors^2^. Although most PD cases (~90%) are sporadic, genetic susceptibility plays a role in the disease pathogenesis, with at least 28 mutated genes linked to familial PD^3^, including the α-synuclein (*SNCA*) and leucine-rich repeat kinase 2 (*LRRK2*) genes. α-Synuclein protein (αSyn) is widely expressed in the brain, especially in presynaptic termini, where it regulates synaptic vesicle trafficking^4^. It exists in dynamic equilibrium amongst different multimeric forms ranging from soluble monomers to higher-order oligomers^5^. Accumulation of misfolded αSyn aggregates in the form of Lewy bodies is a key neuropathological hallmark of PD^6^. Diminished catabolism of misfolded αSyn protein^7^ and/or its increased propensity to undergo self-oligomerization due to conformational changes^8^, or increased synthesis of the wild-type (WT) protein as a result of gene multiplication^9^ are likely causes of the formation of toxic, misfolded αSyn oligomers. Studies suggest that these oligomers can propagate through the brain, most importantly, in dopaminergic nigrostriatal pathways, including the substantia nigra pars compacta (SNpc), striatum and cerebral cortex^10^. The accumulation and propagation of these toxic αSyn oligomers in the brain are integral to the pathogenesis of PD and correlate with disease progression^11^.

Mutations in LRRK2 represent one of the most common genetic risks of familial PD^12^. LRRK2-PD shows clinical and neuropathological features similar to those of sporadic PD, with a similar age of onset^13^. LRRK2 is a multifunctional and multi-domain protein with kinase and GTPase activities^14^. In particular, pathogenic LRRK2^R1441G^ mutation occurs in the Ras-of-complex (Roc) GTPase domain and results in a toxic gain-of-function in kinase activity. Earlier studies suggest that hyper-kinase activity of mutant LRRK2 may be responsible for phosphorylation of αSyn that may trigger its accumulation and aggregation^15^. Discriminant function analysis also revealed high levels of αSyn oligomers in cerebrospinal fluid of asymptomatic *LRRK2* mutation carriers that discriminated from both symptomatic PD and healthy controls^16^. Mouse primary neurons expressing the PD-linked LRRK2^G2019S^ mutant protein have shown increased αSyn aggregation^17^. Interestingly, treatment with the LRRK2 inhibitor (HG-10-102-01) in αSyn transgenic mice reduced levels of αSyn phosphorylation and aggregation in the brain^18^. These results suggest that αSyn aggregation and propagation in PD may be linked to LRRK2 activity, and aberrant hyper-kinase activity associated with mutant LRRK2 could be a potential therapeutic target to modify disease progression in PD^19^. Clinical trials to test the safety of brain-penetrant LRRK2 inhibitors in PD patients are ongoing but lack reliable biomarkers to monitor therapeutic efficacy^1^. Potential abnormalities in the immune system and in tissues which express LRRK2, such as lung and kidneys observed in LRRK2 knockout animals and non-human primates, raise safety concerns over long-term LRRK2 inhibition in PD^20^.

Previously, we generated a mutant LRRK2^R1441G^ knock-in mouse model carrying the homozygous mutation with reduced GTPase and increased kinase activities^21^. Our previous study has established that αSyn oligomers in LRRK2^R1441G^ mutant mouse brains start to accumulate at age 14 months and become significantly increased by age 18 months compared to their WT littermates^7^. The progressive increase in striatal and cortical αSyn oligomers with age in our LRRK2 mutant mice provides a reliable biomarker to monitor synucleinopathy in the brain. Here, we utilized our mutant LRRK2^R1441G^ mice to explore whether (a) long-term treatment with a specific brain-penetrant LRRK2 kinase inhibitor (GNE-7915) is efficacious and safe in alleviating the accumulation of αSyn oligomers in the brain, (b) diminution of αSyn oligomer levels correlates with changes in Ser129-αSyn phosphorylation levels and with two other potential pre-clinical biomarkers of LRRK2 kinase activity–phosphorylation levels of Thr73-Rab10 and Ser106-Rab12, and c) our long-term LRRK2 inhibitor treatment causes any systemic inflammation, infection or peripheral side effects in lung, kidney or liver.

## Results

### Single subcutaneous GNE-7915 dose reduced lung LRRK2 kinase activity, which dissipated by 72 h

Wild-type (WT) LRRK2 exists as a phosphorylated protein under basal conditions^22^, and is constitutively phosphorylated at Ser935 in its LRR domain. This residue is dephosphorylated after kinase inhibition by LRRK2 inhibitors as an indirect measure of LRRK2 kinase activity^23^. However, LRRK2^R1441G^ mutant mice showed significantly lower levels of pSer935-LRRK2 level in the lung (↓85% of WT, *p* < 0.001) and striatum (↓68% of WT, *p* < 0.01) compared to WT (Supplementary Fig. 1). Hence, pSer935-LRRK2 levels did not reliably reflect kinase activity in our mutant mice. Moreover, Ser1292-LRRK2 autophosphorylation has been proposed as an alternative marker of LRRK2 activity^24^. However, pSer1292-LRRK2 levels in our mutant mouse brains were too low to be accurately quantified by Western blot (data not shown). Instead, we employed phosphorylation of Thr73-Rab10 (pRab10) and Ser106-Rab12 (pRab12), known bona fide LRRK2 kinase substrates in vivo^25^, as markers to reflect LRRK2 kinase activity.

**Fig. 1:**
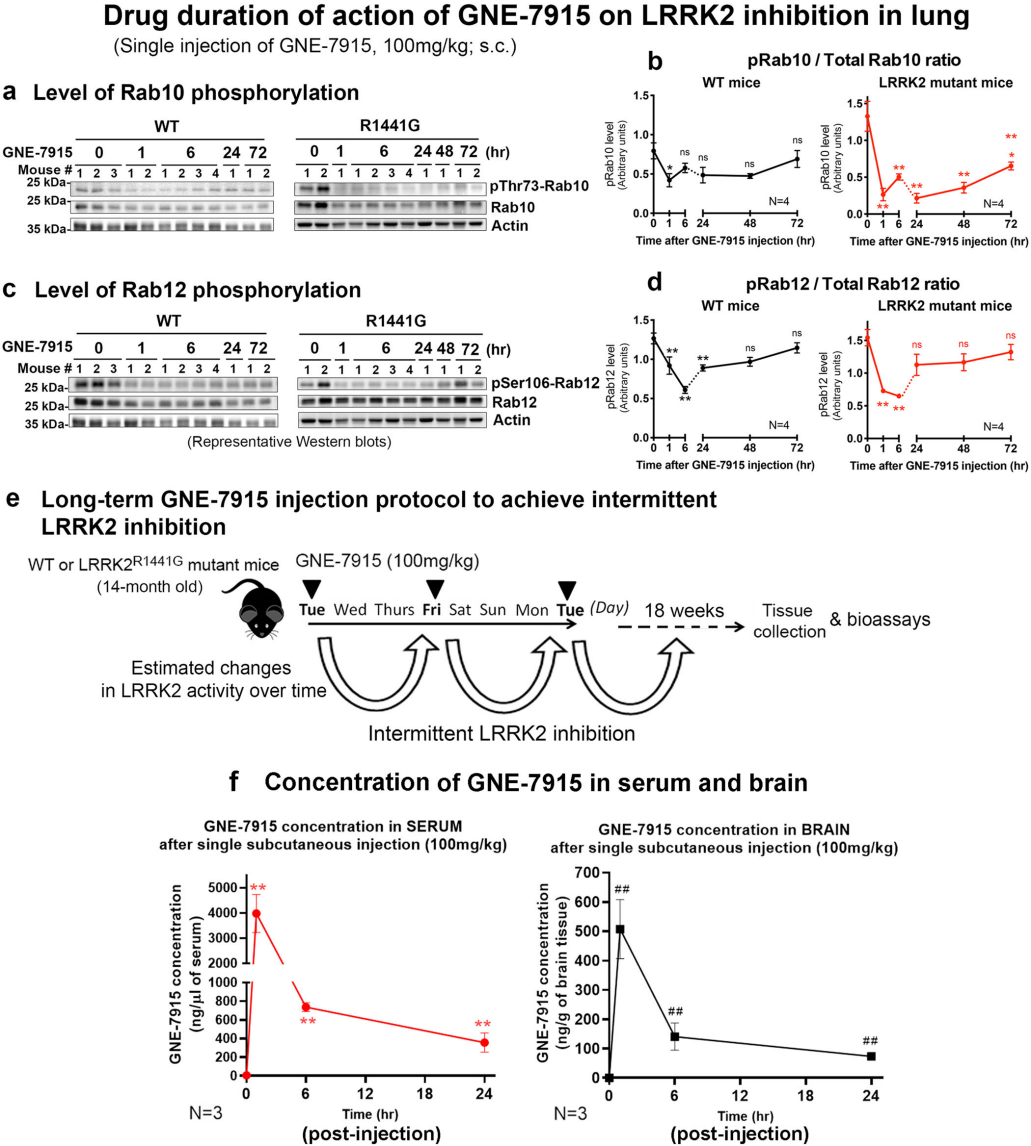
Drug duration of action of GNE-7915 on LRRK2 kinase inhibition. To determine efficacy and duration of GNE-7915 on LRRK2 inhibition, **a** pThr73-Rab10 and **c** pSer106-Rab12 levels in 3-month old WT and LRRK2^R1441G^ mutant mouse lungs were determined by western blotting at 1, 6, 24, 48 and 72 h after single GNE-7915 dose (100 mg/kg; s.c.). **b** pRab10 and **d** pRab12 levels were reduced after injection which reached maximum inhibition within 24 h. Kinase inhibition dissipated gradually with pRab10 and pRab12 levels returning to basal levels by 72 h. Data are expressed as mean ± SEM (*N* = 4). **p < 0.01 and *p < 0.05 represent statistical significance between the designated time point to its corresponding basal levels (time 0) by one-way ANOVA with post hoc Bonferroni’s multiple comparison correction. **e** Design of long-term GNE-7915 injection protocol to achieve intermittent LRRK2 inhibition over 18 weeks. **f** Bioavailability of GNE-7915 in serum and brain. GNE-7915 concentration in mouse brain and serum was quantified at different time points using LC-MS-MS after single subcutaneous injection of GNE-7915 at 100 mg/kg. Data are expressed as mean ± SD (*N* = 3 per time point). **p < 0.01 and ^##^*p* < 0.01 represent statistical significance between the designated time point to its corresponding basal levels (time 0) in serum and brain, respectively, using unpaired Student’s *t*-test.

LRRK2 kinase activity was determined by levels of pRab10 and pRab12 in lungs of WT and mutant mice at different time points after a single GNE-7915 subcutaneous injection (100 mg/kg, s.c.) (Fig. 1). In WT lungs, both pRab10 and pRab12 levels decreased 1 h after injection, and maximum kinase inhibition was reached at 24 h as measured by pRab10 (51%) and at 6 h as measured by pRab12 (54%) (all *p* < 0.05) (Fig. 1a–d). Similarly, in mutant mice, lung pRab10 and pRab12 levels were reduced 1 h post-injection, reaching maximum kinase inhibition as measured by pRab10 (88%) at 24 h and pRab12 (52%) at 6 h (all *p* < 0.05). Kinase inhibition gradually dissipated in both WT and mutant mice after 24 h, with pRab10 and pRab12 returning to their basal levels by 72 h (Fig. 1a–d).

### Long-term (18 weeks) GNE-7915 treatment regimen

Based on the duration of drug effects from our single dose experiments, we formulated a long-term injection protocol to achieve partial and intermittent LRRK2 kinase inhibition over 18 consecutive weeks. We aimed to achieve efficacy in reducing αSyn oligomer levels in mutant brains with minimal side effects from excessive LRRK2 inhibition in peripheral tissues. Fourteen-month-old (equivalent to 47 human years^26^) WT and LRRK2^R1441G^ mutant mice with similar weight were randomly assigned to receive injections of either vehicle or GNE-7915 (100 mg/kg; s.c.) every 3 or 4 days (twice weekly) for 18 consecutive weeks (equivalent to ~13 human years) until they reached about 18 months of age (Fig. 1e). GNE-7915 was administrated as freshly prepared suspensions in drug vehicle via subcutaneous injections to ensure accurate dosing, and avoid confounding differences in feeding behaviour and trauma from oral gavage.

### GNE-7915 concentrations in serum and brain following subcutaneous injection

We determined concentrations of GNE-7915 in both the whole brain and serum following a single subcutaneous dose (i.e. 100 mg/kg) of the drug using liquid chromatography/tandem mass spectrometry (LC-MS-MS). GNE-7915 was freshly dissolved in the drug vehicle at 100 mg/kg based on the corresponding body weight. Fourteen-month-old wild-type mice from different litters were randomly divided into four groups (*N* = 3) and treated for four different durations (i.e. 0, 1, 6 or 24 h) prior to sacrifice. The mice were euthanized after drug administration before serum, and whole brain samples were collected for the quantification of GNE-7915. Our LC-MS-MS results showed that GNE-7915 was detected in both serum and brain at 1 h after a single subcutaneous injection. GNE-7915 concentration reached a peak of 3980 ± 434 ng/ml (or 8.98 ± 0.98 µM) in serum at 1 h after injection and decreased gradually to 356 ± 59 ng/ml (or 0.803 ± 0.13 µM) after 24 h (all *p* < 0.01; *N* = 3; unpaired *t*-test). GNE-7915 was also detected in the brain, reaching a peak concentration of 508 ± 58 ng/g at 1 h after injection, then the dose was gradually decreased to 103 ± 7 ng/g at 24 h (all *p* < 0.01; *N* = 3; unpaired *t*-test) (Fig. 1f). These results demonstrate effective drug delivery of GNE-7915 to the brain using our treatment regimen.

### Long-term GNE-7915 inhibited mutant LRRK2 hyper-kinase activity to WT levels

**Fig. 2:**
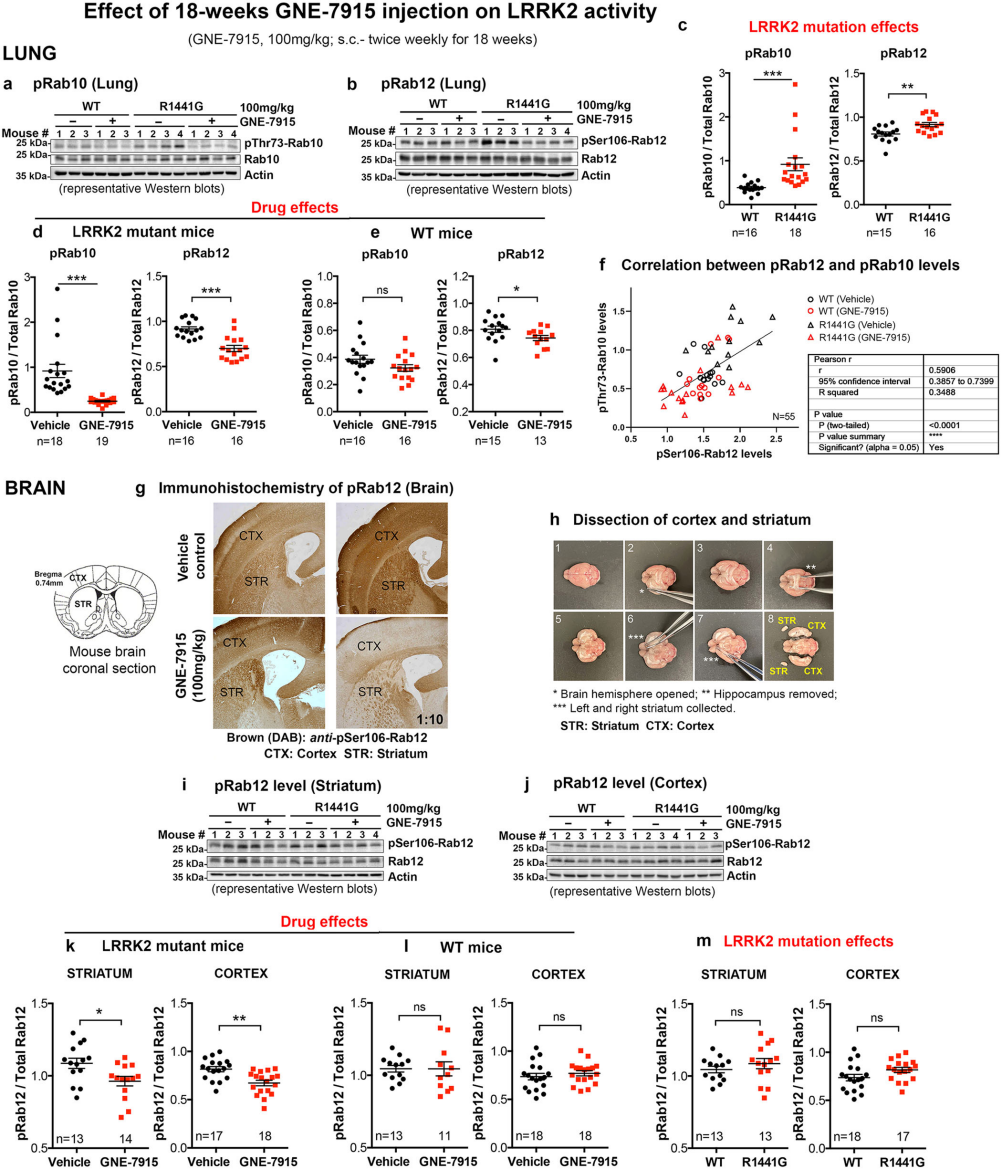
Effects of 18-week GNE-7915 treatment regimen on pThr73-Rab10 and pSer106-Rab12 levels in mouse lung and brain. **a**, **b** Whole lung lysates of WT and LRRK2^R1441G^ mutant mice were analyzed by western blot using a phosphor-specific antibody against pThr73-Rab10 and pSer106-Rab12. **c** Quantification shown based on ratio of pRab10/total Rab10 and pRab12/total Rab12 indicated LRRK2 hyperactivity in LRRK2^R1441G^ mutant mice (i.e. LRRK2 mutation effects). **d**, **e** Significant reduction in pThr73-Rab10 and pSer106-Rab12 levels in mutant lungs indicating attenuation of LRRK2 hyperactivity in LRRK2 mutant mice (i.e. drug effects). **f** Correlation plot between pRab10 and pRab12 in lung showed a significant positive correlation (Pearson correlation test: r = 0.5906, *p* < 0.0001; *N* = 55). **g** Immunohistochemistry of pRab12 in cortex and striatum. GNE-7915 treatment resulted in reduced pSer106-Rab12 staining in mutant but not in WT mice. **h** Whole cortex and striatum of the brain were freshly harvested for western blotting of pRab12 in **i** striatum and **j** cortex. GNE-7915 significantly reduced pSer106-Rab12 levels in **k** mutant but not in **l** WT mice, in both cortex and striatum. **m** pSer106-Rab12 levels in cortex and striatum were similar between vehicle-treated WT and LRRK2 mutant mice. Data were expressed as mean ± SEM. *X*-axis numbers represent total number of animals in each treatment group. Datasets were subjected to D’Agostino & Pearson normality test prior to statistical analysis. *p < 0.05, **p < 0.01 and ***p < 0.001 represent statistical significance between two designated groups by unpaired, parametric Student’s *t*-test. “ns” not significant. Detailed statistical analysis results of relevant comparisons including (1) D’Agostino & Pearson normality test, (2) Grubb’s test, (3) unpaired, parametric Student’s *t*-test and (4) non-parametric Mann–Whitney U-test were shown in the supplementary section (Supplementary Table 1).

Levels of pRab10 and pRab12 in lung lysates of vehicle-treated mutant mice were higher than in age-matched vehicle-treated WT mice by 163% and 17%, respectively (all *p* < 0.01), confirming hyperactive LRRK2 kinase activity in the mutants (Fig. 2a–c). Long-term GNE-7915 (100 mg/kg per dose; s.c., twice weekly for 18 weeks) reduced pRab10 and pRab12 levels in mutant lungs by 73% and 24%, respectively, compared with levels in their corresponding vehicle-treated controls, measured at 24 h following the final dose of the drug (all *p* < 0.01) (Fig. 2d, e). Two-way ANOVA analysis showed GNE-7915 treatment had a significant effect on both pRab10 (*p* < 0.0001) and pRab12 levels (*p* < 0.0001) in lung. Significant interaction was observed between LRRK2 mutation and GNE-7915 effects on both pRab10 (*p* < 0.001) and pRab12 (*p* < 0.01). Post hoc Tukey’s multiple comparisons showed a significant reduction of both pRab10 (*p* < 0.0001) and pRab12 (*p* < 0.0001) levels in GNE-7915-treated LRRK2 mutant mice but not WT mice. Other relevant statistical comparisons are shown in Supplementary Table 1. Furthermore, correlation analysis showed that pRab10 and pRab12 in lungs are significantly correlated in response to GNE-7915 treatment (r = 0.5906; *p* < 0.0001) (Fig. 2f), confirming that these two LRRK2-related enzymes (substrates) are valid markers of LRRK2 inhibition in lungs^25^. More importantly, mutant LRRK2 kinase hyperactivity, as indicated by increased pRab10 and pRab12 levels, was significantly inhibited after treatment to physiological levels seen in vehicle-treated WT mice, indicating that our LRRK2 inhibitor treatment regimen was not excessive. Interestingly, similar long-term treatment with GNE-7915 reduced pRab12 (*p* < 0.05) but not pRab10 levels in WT mice (Fig. 2d, e), unlike the single dose at 24 h and 48 h, which reduced both pRab10 and pRab12 levels (Fig. 1a–d).

Relative LRRK2 kinase activities were demonstrated by the levels of pRab10 and pRab12 in two brain regions (cortex and striatum) of WT and mutant mice after long-term GNE-7915 (Fig. 2g–m). Unlike in the lungs, pRab10 levels in the brain were highly varied among different mice, even within the same treatment group (Supplementary Fig. 2), rendering it unreliable as a marker of brain LRRK2 activity. In contrast, the pRab12 level was comparatively more stable in the brain, in accord with a previous study^27^. Following treatment with our immunohistochemistry protocol, visual examination of the colorimetric immunostaining intensity of pRab12 was apparently higher in the cortex and striatum of our LRRK2 mutant mice than in WT, which was reduced after GNE-7915 treatment (Fig. 2g). Importantly, our Western blot analysis did not show a statistically significant difference in pRab12 levels between vehicle-treated LRRK2 mutant and WT mice in both cortex and striatum (Fig. 2m). In contrast, our long-term twice-weekly GNE-7915 treatment regimen was sufficiently potent to reduce pRab12 levels in mutant cortex and striatum by 18% (*p* < 0.01) and 11% (*p* < 0.05), respectively, compared with levels in their corresponding vehicle controls (Fig. 2k), demonstrating effective inhibition of hyperactive mutant LRRK2 activity to physiological levels during the entire course of treatment. Two-way ANOVA analysis also revealed a significant interaction between LRRK2 mutation and GNE-7915 effects only in the cortex (*p* < 0.01) but not in the striatum. Interestingly, long-term GNE-7915 treatment did not affect pRab12 levels in WT cortex or striatum (Fig. 2l), indicating that our treatment regimen was effective only in reducing hyper-kinase activity in the mutant brain to normality but not in WT mice with normal LRRK2 activity. Other relevant statistical comparisons are shown in Supplementary Table 1.

### αSyn oligomers decreased in the striatum of mutant mice after long-term GNE-7915

**Fig. 3:**
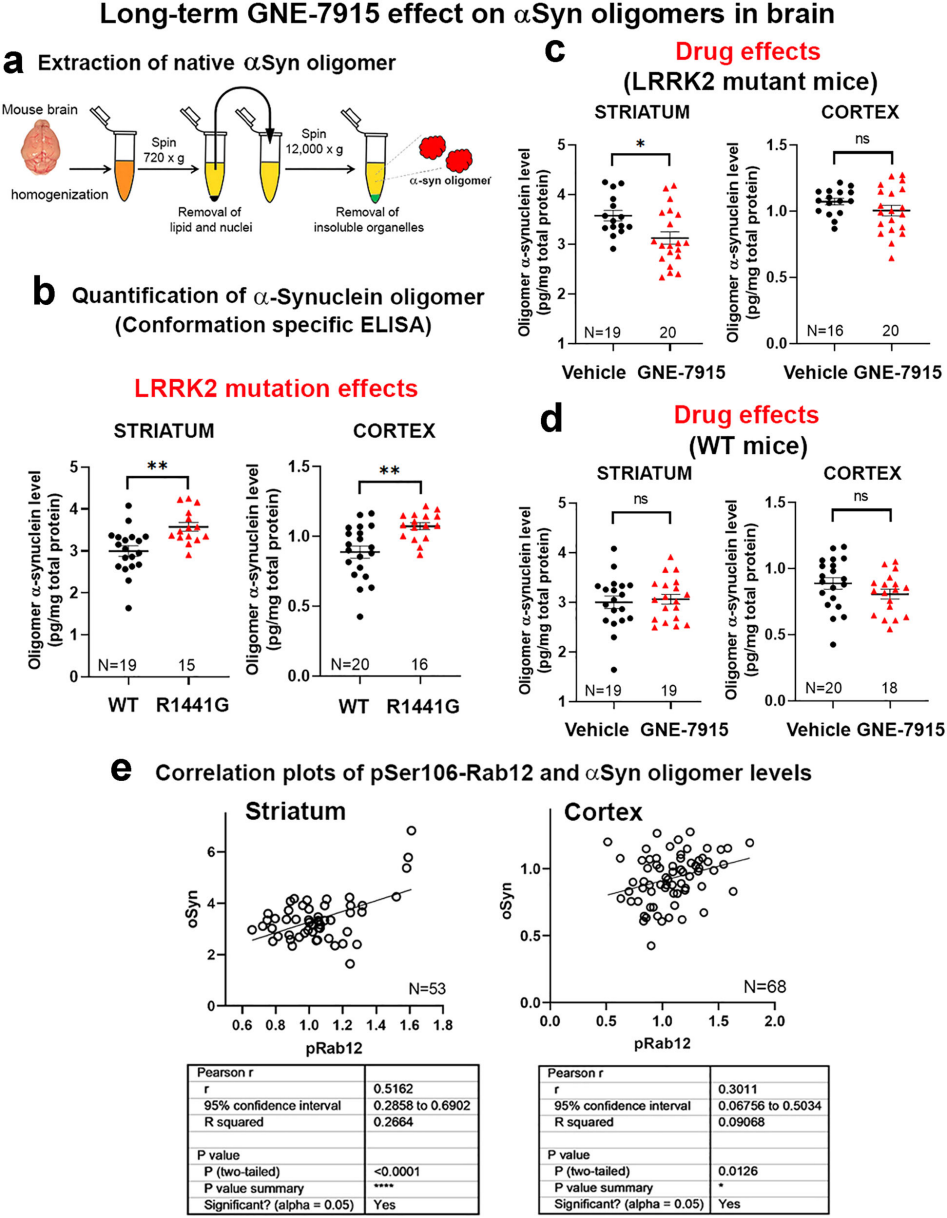
Long-term GNE-7915 effect on αSyn oligomers in LRRK2^R1441G^ mutant brain. **a** Extraction of αSyn oligomer in cortex and striatum of aged WT and LRRK2 mutant mice after long-term GNE-7915. Snap-frozen tissues were sonicated in ice-cooled PBS to preserve native conformation of oligomers. **b**–**d** Supernatants containing soluble oligomers were quantified by mouse αSyn oligomer ELISA. Total soluble oligomer levels (pg/mg total protein) based on linear standard curve generated from standards provided by the kit. **e** Correlation plot between pSer106-pRab12 (pRab12) and αSyn oligomer levels showed a significant positive correlation in striatum (Pearson correlation test: r = 0.5162, *p* < 0.0001; *N* = 53) and cortex (Pearson correlation test: r = 0.3011, *p* < 0.0126; *N* = 68). Data expressed as mean ± SEM. *X*-axis numbers represent total number of animals in each treatment group. Datasets were subjected to D’Agostino & Pearson normality test prior to statistical analysis. Outliers were identified by Grubb’s test. *p < 0.05 and **p < 0.01 represent statistical significance between two designated groups by unpaired, parametric Student’s *t*-test. “ns” not significant. Detailed statistical analysis results of relevant comparisons including (1) D’Agostino & Pearson normality test, (2) Grubb’s test, (3) unpaired, parametric Student’s *t*-test and (4) non-parametric Mann–Whitney U-test were shown in the supplementary section (Supplementary Table 1).

αSyn oligomers in striatum and cortex were quantified using a commercially validated, conformational-specific ELISA (Fig. 3a). Validation of assay specificity confirmed that this ELISA does not cross-react with two independent negative controls, (1) total cell lysates extracted from mouse embryonic fibroblasts (MEFs; which does not express αSyn), and (2) a purified recombinant monomeric αSyn protein (Abcam™; #ab218818) (Supplementary Fig. 3). Two-way ANOVA analysis revealed significant effects of LRRK2 mutation (*p* < 0.01) and GNE-7915 (*p* < 0.05) on αSyn oligomers in the striatum. A significant interaction was also observed between LRRK2 mutation and GNE-7915 effects (*p* < 0.01). Post hoc multiple comparisons showed that striatal αSyn oligomer levels were higher in vehicle-treated mutant compared to WT mice (WT-vehicle: 2.998 ± 0.12 vs. R1441G-vehicle: 3.968 ± 0.239 pg/mg total protein; *p* < 0.01) (Fig. 3b), consistent with our previous findings that LRRK2^R1441G^ mutation increased αSyn oligomer levels in brain^7^. Absolute αSyn oligomer levels in the cortex were more than three-fold lower than striatum in both WT and mutant mice (Fig. 3b). Following long-term treatment, striatal αSyn oligomer levels in GNE-7915-treated mutant mice were reduced (*p* < 0.01) (Fig. 3c), indicating that our long-term treatment regimen was efficacious in restoring the abnormally high αSyn oligomer levels in mutant to normal physiological levels seen in WT mice. Unlike mutant mice, αSyn oligomer levels in WT striatum showed no significant difference after treatment (Fig. 3d), consistent with the similar kinase activities found between vehicle- and GNE-7915 treated WT (Fig. 2l). Furthermore, correlation analysis showed that the striatal αSyn oligomer level was positively correlated with pRab12 (r = 0.5162; *p* < 0.0001), indicating that reduction of αSyn oligomers was associated with LRRK2 activity (Fig. 3e).

Similar to the results in the striatum, the αSyn oligomer level in the cortex was higher in vehicle-treated mutant mice compared with that in WT controls (WT-vehicle: 0.888 ± 0.043 vs. R1441G-vehicle: 1.048 ± 0.033 pg/mg total protein; *p* < 0.5) (Fig. 3b), consistent with our previous findings^7^. Two-way ANOVA analysis revealed a significant effect of the LRRK2 mutation (*p* < 0.01) on αSyn oligomer levels in the cortex. However, unlike striatum, long-term GNE-7915 treatment did not reduce cortical oligomer levels in both WT or mutant mice (Fig. 3d). Furthermore, no significant interaction was found between LRRK2 mutation and GNE-7915 effects in the cortex. Other related statistical comparisons are shown in Supplementary Table 1. Furthermore, levels of αSyn oligomers and pRab12 in the cortex only showed a weak positive correlation (r = 0.3011; *p* < 0.0126) (Fig. 3e).

### GNE-7915 inhibited Ser129-αSyn phosphorylation in human neuronal SH-SY5Y cells, stably overexpressing αSyn

**Fig. 4:**
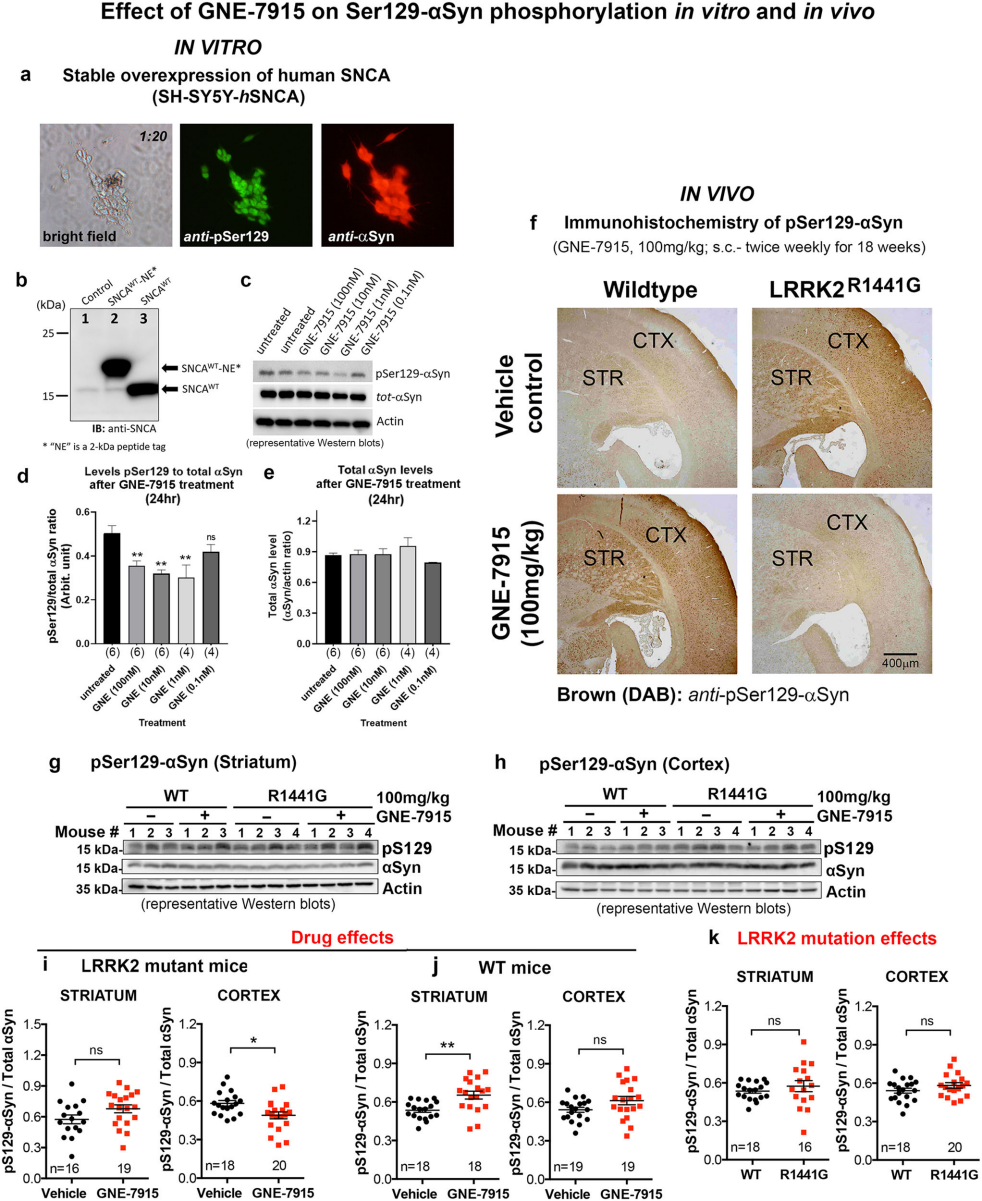
GNE-7915 inhibited Ser129-αSyn phosphorylation in vitro and in vivo. **a** Lentiviral-mediated overexpression of human αSyn in human SH-SY5Y cells. Immunocytochemistry of demonstrated αSyn overexpression (red) and pSer129-αSyn (green); **b** Normal SH-SY5Y cells expressed low level of αSyn (control lane). Two selected stable clones (SNCA^WT^ and SNCA^WT^-NE) overexpressed αSyn with/without NE-tag^7^; **c** Representative immunoblot of pSer129-αSyn in SNCA^WT^ cells after GNE-7915 treatment. **d**, **e** pSer129-αSyn level was determined as pSer129/total αSyn ratio, and compared with that of DMSO-treated controls. Total cellular αSyn level was determined by total αSyn/actin ratio. Statistical differences after GNE-7915 treatments were determined by one-way ANOVA with post hoc Dunnett’s multiple comparisons test. *X*-axis numbers represent number of independent cell treatments. *p < 0.05 and **p < 0.01 represent statistical significance between untreated control and the designated treatment group. **f** Immunohistochemistry of pSer129-αSyn in cortex and striatum of WT and LRRK2^R1441G^ mutant mice treated with either drug vehicle or GNE-7915 (100 mg/kg, s.c.; twice weekly) for 18 weeks; **g**–**k** Relative quantification of total and pSer129-αSyn levels was based on immunoblotting of SDS-soluble cortical and striatal total lysates. Data are expressed as mean ± SEM. *X*-axis numbers represent total number of animals in each treatment group. Datasets were subjected to D’Agostino & Pearson normality test prior to statistical analysis. Potential outliers were identified by Grubb’s test. Interaction between LRRK2 mutation and GNE-7915 treatment in cortex and striatum was determined by two-way ANOVA as described in the relevant Results section. *p < 0.05 and **p < 0.001 represent independent statistical comparison between two designated groups by unpaired, parametric Student’s *t*-test. “ns” not significant. Detailed statistical analysis results of relevant comparisons including (1) D’Agostino & Pearson normality test, (2) Grubb’s test, (3) unpaired, parametric Student’s *t*-test and (4) non-parametric Mann–Whitney U-test were shown in the supplementary section (Supplementary Table 1).

Phosphorylation of αSyn at residue Ser129 (pSer129) has been considered a surrogate marker of synucleinopathies in PD^28^. We explored whether LRRK2 inhibition can modulate Ser129-αSyn phosphorylation using human SH-SY5Y cells which overexpressed WT αSyn. Immunocytochemistry confirmed αSyn overexpression stained in red and pSer129-αSyn in green (Fig. 4a). Cells were treated with GNE-7915 (dose ranging from 0.1 to 100 nM) or vehicle (0.01% DMSO) for 24 h. Western blots showed pSer129-αSyn levels in cells treated with GNE-7915 at 1, 10 and 100 nM were significantly reduced compared to DMSO-treated controls (↓40.1%, ↓36.4% and ↓29.3%, respectively, all *p* < 0.01) (Fig. 4b–d). Total αSyn level was not affected after GNE-7915 treatment at all doses (Fig. 4e).

The effect of LRRK2 inhibition on αSyn phosphorylation was validated by using a second LRRK2 inhibitor, MLi-2, in our LRRK2 mutant mouse embryonic fibroblasts (MEFs), which were engineered to express high levels of recombinant αSyn protein^7^. MLi-2 treatment (100 nM) for 24 h significantly reduced pRab10 phosphorylation in a dose-dependent manner in our LRRK2 mutant MEFs, indicating significant LRRK2 kinase inhibition (Supplementary Fig. 4). MLi-2 (10 and 100 nM) demonstrated a similar effect in reducing Ser129-αSyn phosphorylation as in mouse brain after GNE-7915 injection (Supplementary Fig. 4).

### pSer129-αSyn level in LRRK2 mutant brain cortex was reduced after long-term GNE-7915 treatment

We compared endogenous levels of pSer129-αSyn in different brain regions of 3-month-old WT mice. Western blots for tyrosine hydroxylase [TH; a marker of dopaminergic neurons] showed that the olfactory bulb and striatum had intense staining indicating they were highly enriched with dopamine neurons, whereas the cortex and midbrain had less, and the cerebellum and hippocampus had little or no TH stain. (Supplementary Fig. 5). The highest levels of total αSyn were observed in the cortex, followed by the striatum, olfactory bulb, hippocampus, midbrain and cerebellum. pSer129-αSyn level was highest in the olfactory bulb, followed by the hippocampus, cortex, striatum and midbrain in descending order. pSer129-αSyn in the cerebellum was barely detectable (Supplementary Fig. 5).

We then investigated whether reduced αSyn oligomers seen in GNE-7915-treated mutant brains correlated with pSer129-αSyn levels. Immunohistochemistry of 18-month-old LRRK2 mutant mice exhibited more intense staining of pSer129-αSyn than WT mice in both cortex and striatum (Fig. 4f). Long-term GNE-7915 reduced pSer129-αSyn staining in cortex and striatum of LRRK2 mutant mice compared to vehicle treatment (Fig. 4f). Surprisingly, similar GNE-7915 treatment increased pSer129-αSyn staining in WT mice compared with vehicle treatment (Fig. 4f).

The effects of long-term GNE-7915 on brain Ser129-αSyn phosphorylation were further investigated by western blotting (Fig. 4g–j). Phosphorylation levels of Ser129-αSyn (pSer129-αSyn/total αSyn ratio) between vehicle-treated WT and LRRK2 mutant mice were similar in both cortex and striatum (Fig. 4k). However, two-way ANOVA analysis showed a significant interaction between LRRK2 mutation and GNE-7915 treatment in the cortex (*p* < 0.01), but not in striatum. Independent *t*-test analysis in LRRK2 mutant mice demonstrated significant drug effects in reducing pSer129-αSyn level in the mutant cortex after long-term GNE-7915 treatment (*p* < 0.05), but not in the striatum (Fig. 4i). Surprisingly, unlike mutant mice, GNE-7915 treatment increased pSer129-αSyn level in the striatum compared with the level in vehicle-treated WT mice (*p* < 0.01), whereas cortex was not affected after GNE-7915 treatment (Fig. 4j).

### GNE-7915 did not affect αSyn clearance rate in LRRK2 mutant embryonic fibroblasts

As long-term GNE-7915 reduced αSyn oligomer levels in LRRK2 mutant mice, we explored whether GNE-7915 affected cellular clearance of αSyn in mutant mouse-derived embryonic fibroblasts (MEFs). Immortalized mutant MEFs expressing PAmCherry-αSyn^7^ were photoactivated and treated with GNE-7915 (100 nM) for 72 h. Mean intensities of fluorescence emitted by photoactivated PAmCherry-αSyn in LRRK2 mutant cells were measured by flow cytometry at different time points (0, 24, 48 and 72 h) to establish a substrate clearance curve. GNE-7915 treatment for 72 h did not affect overall αSyn clearance compared with that of DMSO-treated mutant cells (Supplementary Fig. 6), suggesting that GNE-7915 had no effect on cellular αSyn clearance.

### Long-term GNE-7915 did not affect nigrostriatal dopaminergic cell viability

**Fig. 5:**
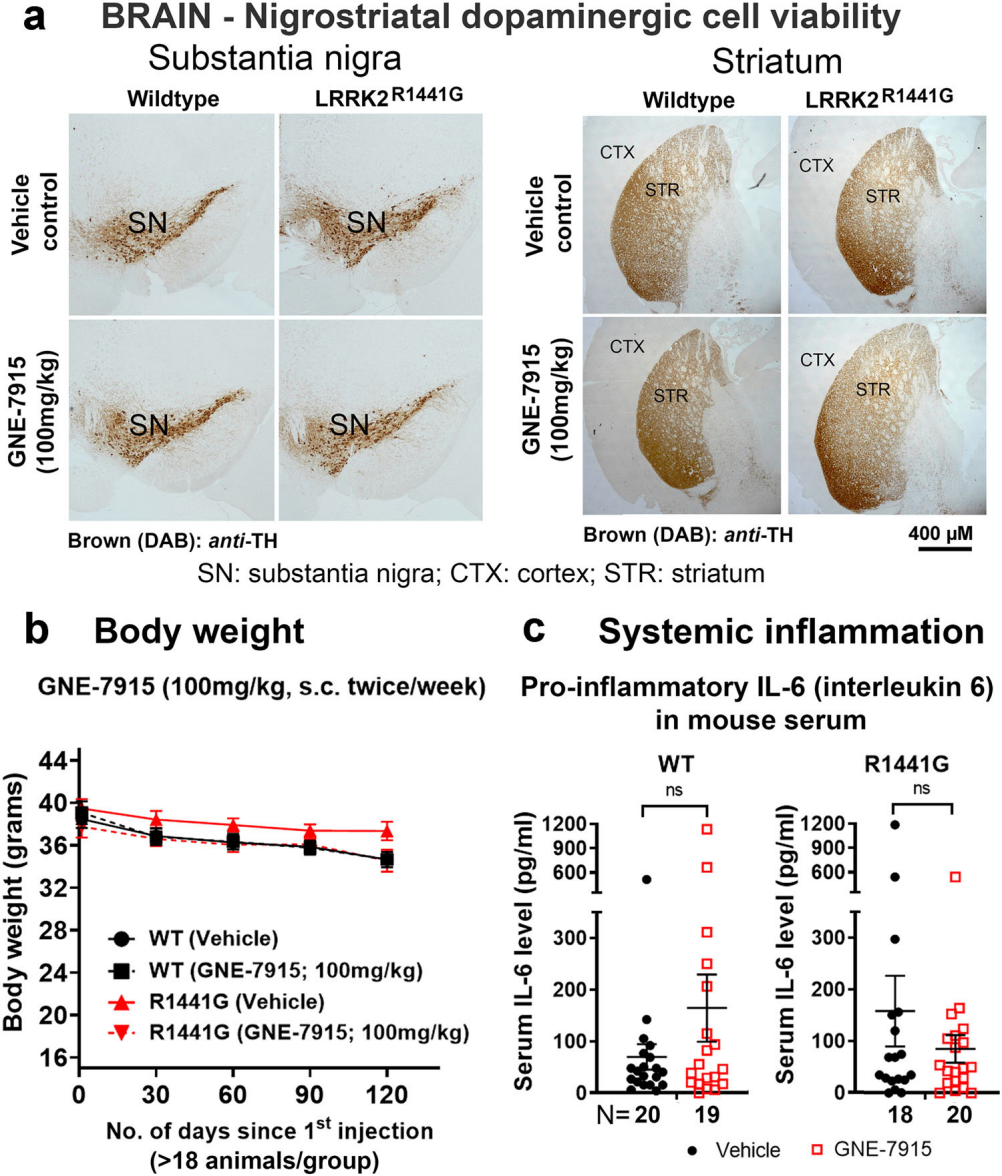
No abnormal brain nigrostriatal cell death, body weight loss or systemic inflammatory response after long-term GNE-7915. **a** Representative immunohistochemistry of tyrosine hydroxylase (TH; DA cell marker) to visualize nigrostriatal dopaminergic neurons in substantia nigra and striatum. Long-term treatment did not affect DA cell viability and morphological integrity of striatal neural network in both WT and LRRK2 mutant mice (*N* = 2 per treatment group). **b** Changes in body weight of each mouse were recorded monthly. There were no significant differences in mean body weight between WT and mutant mice, and between vehicle- and GNE-treated mice (*N* ≥ 18 per group). **c** Mouse serum was collected 24 h after the last injection for IL-6 levels quantified by ELISA. Long-term treatment did not affect serum IL-6 levels in both WT and LRRK2 mutant mice (*N* ≥ 18). Data were expressed as mean ± S.E.M. *X*-axis numbers represent total number of animals in each treatment group. Statistical comparison for drug effects between two designated groups was performed independently in WT and LRRK2 mutant mice by unpaired, Mann–Whitney U-test, a non-parametric test because of unequal variances. “ns” not significant.

We examined the effects of long-term GNE-7915 treatment on the viability and integrity of nigrostriatal dopaminergic neurons by immunohistochemistry of TH (DA cell marker protein) in the substantia nigra and striatum. No observable differences were seen in gross morphology and TH staining intensity between vehicle-treated WT and LRRK2 mutant mice and between vehicle- and GNE-7915-treated mice (Fig. 5a), indicating that long-term GNE-7915 has no observable toxicity on nigrostriatal DA neurons.

### No abnormal body weight loss and signs of systemic inflammatory response were observed after long-term GNE-7915 treatment

The average weight of LRRK2 mutant and WT mice before treatment was similar (mutant: 38.76 ± 0.68 g vs. WT: 38.63 ± 0.69 g). After long-term GNE-7915, there were no significant differences in body weight between WT and mutant mice. GNE-7915 treatment also did not affect body weight in both WT and LRRK2 mutant mice compared with the weight of their corresponding vehicle-treated control mice (Fig. 5b). As LRRK2 is highly expressed in immune cells^29^, we also determined whether GNE-7915 caused any signs of the systemic inflammatory immune response. In particular, we measured circulatory levels of interleukin-6 (IL-6), a pro-inflammatory cytokine observed in infection or inflammation. All GNE-7915-treated mice showed no signs of fatigue and muscle weakness, typical signs of systemic inflammation. IL-6 levels in serum collected at 24 h after the final GNE-7915 injection were quantified by ELISA (assay sensitivity <3 pg/ml). No differences were found in circulatory IL-6 levels between vehicle-treated WT and mutant mice. GNE-7915 did not also affect IL-6 levels in both WT and LRRK2 mutant mice compared with the levels in their corresponding vehicle-treated controls (Fig. 5c).

### No abnormal lung gross morphology, histology or function after long-term GNE-7915

**Fig. 6:**
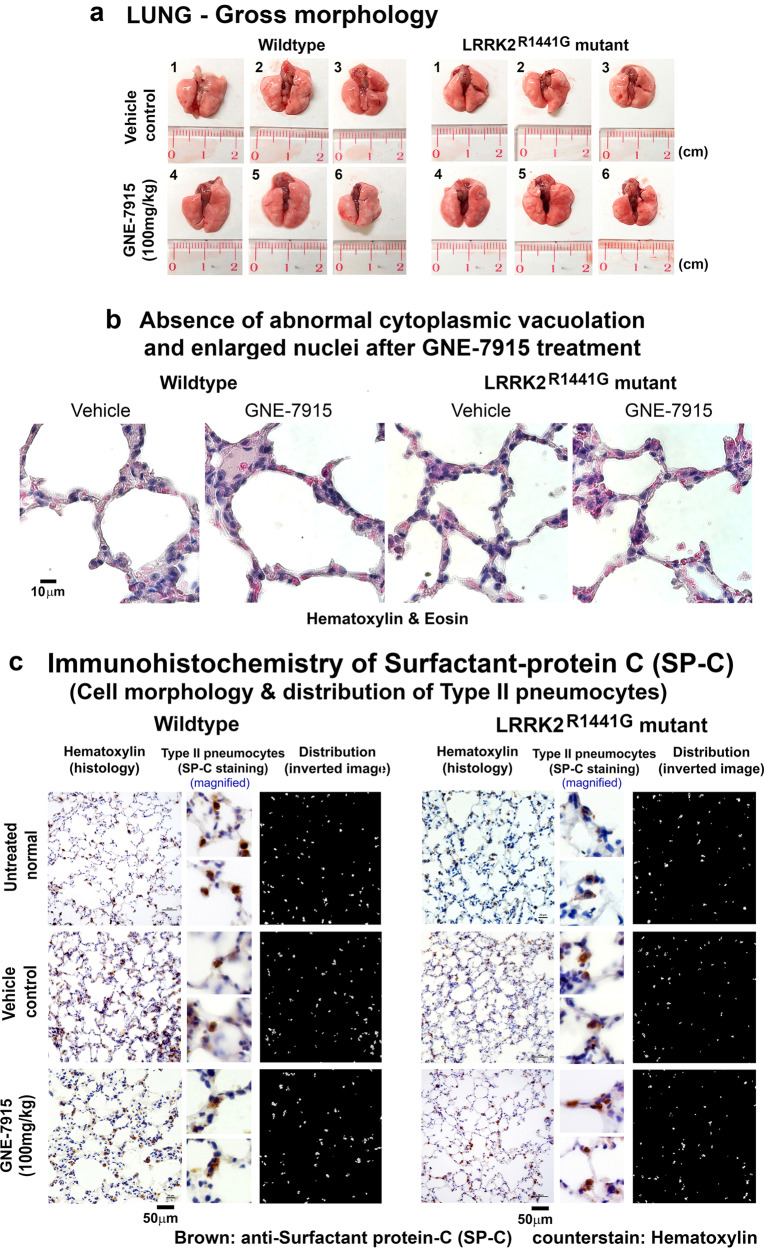
Lung histology after long-term GNE-7915 treatment. **a** Gross lung morphology in GNE-7915- and vehicle-treated WT and LRRK2 mutant mice (*N* ≥ 18). **b** Hematoxylin-eosin (H&E) staining of the lungs showing normal histology without abnormal cytoplasmic vacuolation and enlarged nuclei after GNE-7915 treatment. **c** Representative immunohistochemistry of surfactant protein C (SP-C; protein marker of type II pneumocytes) (brown) did not show abnormalities in lung after GNE-7915 treatment in WT and mutant mice. Magnification: 1 × 40. Two magnified sub-panels of each mouse showed cell morphology of type II pneumocytes (brown). Distribution of type II pneumocytes (white) was shown by inverted photomicrographs. Tissue sections were counterstained by hematoxylin (purple) and eosin (pink) for visualization of cell nuclei and cytoplasm.

LRRK2 inhibition was found to induce distinct lung morphological changes in macaque^20^ and in mice^30^, including cytoplasmic vacuolation and enlarged lamellar bodies in type II pneumocytes associated with abnormal cellular size and organization. We examined whether our long-term GNE-7915 regimen caused similar changes in mouse lungs. Macroscopic examination of the lungs showed no gross morphological changes in organ size and colour in both WT and LRRK2 mutant mice after treatment of either GNE-7915 (100 mg/kg per dose; s.c., twice weekly for 18 weeks) or drug vehicle (Fig. 6a). These lung tissues were sectioned and counterstained with hematoxylin-eosin (H&E) for independent histological examination by a respiratory specialist in a blinded manner. Both GNE-7915 and vehicle-treated mice showed no cytoplasmic vacuolation and enlarged nuclei in pneumocytes (Fig. 6b), contrasting to what was reported in macaque treated with the same drug^20^. Furthermore, we also examined the histology and distribution of type II pneumocytes after staining of surfactant protein C (SP-C; marker of type II pneumocytes), and we found no observable differences in cell size and spatial cellular distribution in both GNE-7915- and vehicle-treated WT and mutant mice (Fig. 6c).

**Fig. 7:**
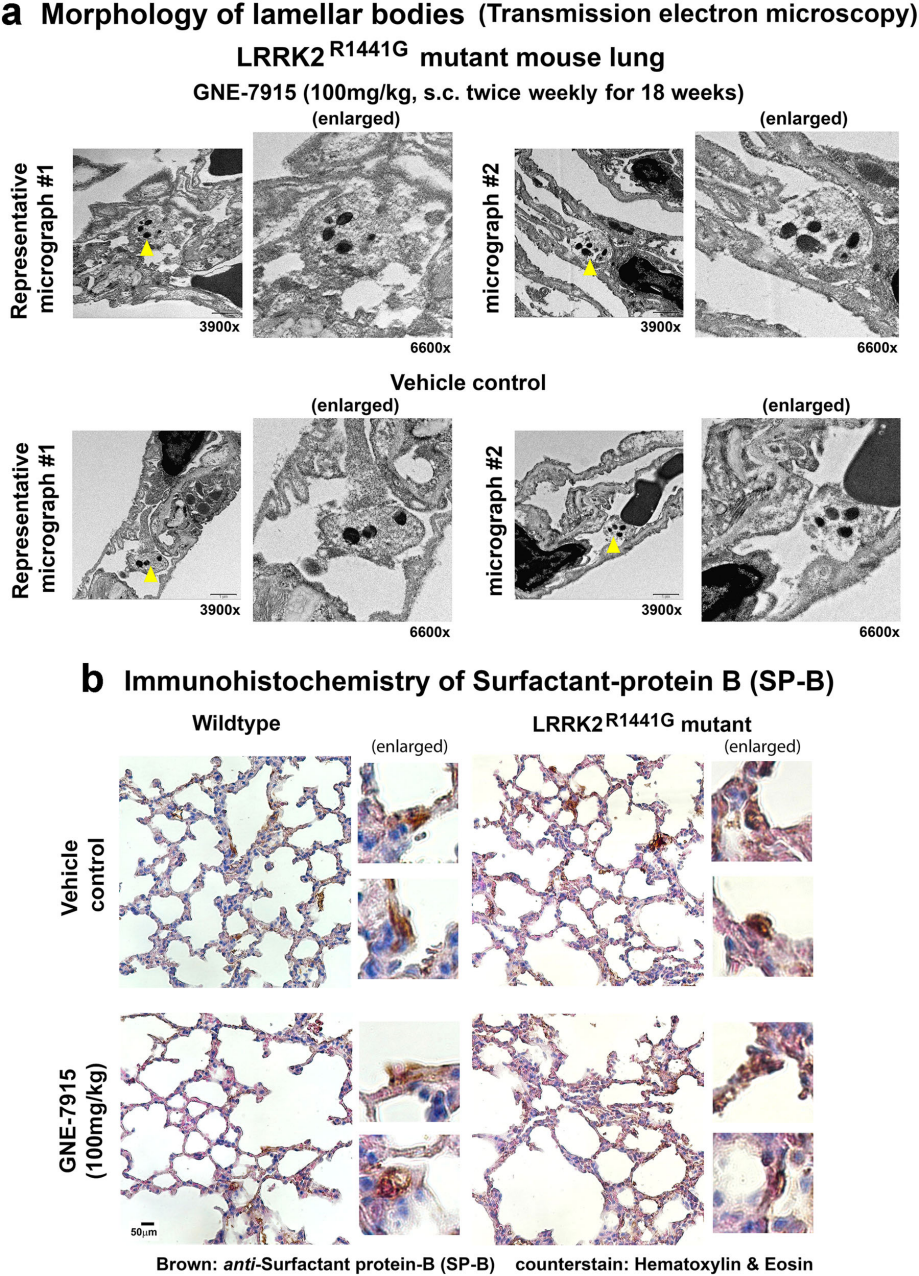
Morphological examination of lamellar bodies in pneumocytes of LRRK2 mutant mice after long-term GNE-7915 treatment. **a** Paraffin-embedded lung tissues for immunohistochemistry were further processed for examination of alveolar cell ultrastructure under transmission electron microscopy (TEM). Lamellar bodies are small electron-dense circular organelles in type II pneumocytes for storage of pulmonary surfactant (*yellow arrow*). No observable differences in the size of lamellar bodies were found in GNE-7915-treated mutant mouse lungs compared to vehicle controls (*N* = 2). Images were taken at a magnification of ×3900. sub-panel to the right of each micrograph showed magnified view of lamellar bodies at ×6600. **b** Representative immunohistochemistry of surfactant protein-B (SP-B; a protein synthesized by type II pneumocytes and stored in lamellar bodies) (brown) showed similar staining pattern between GNE-7915 and vehicle-treated WT and mutant mice (*N* = 3). Magnification: 1 × 40. Two magnified sub-panels of each mouse showed intracellular SP-B staining in magnified pneumocytes (brown). Tissue sections were counterstained by hematoxylin (purple) and eosin (pink) for visualization of cell nuclei and cytoplasm.

Lamellar bodies are small circular secretary organelles which store lung surfactant in type II pneumocytes. They are electron-dense and observed under a transmission electron microscope (TEM). Abnormally enlarged lamellar bodies had been reported to be a side effect of LRRK2 inhibitors in non-human primates^20^. We examined the gross morphology of lamellar bodies in mouse lungs after our long-term GNE-7915 treatment. TEM images of the lungs showed that there were no observable changes in the size of lamellar bodies in GNE-7915-treated mice compared to the vehicle-treated controls (Fig. 7a). Immunostaining of surfactant protein-B (SP-B; a surfactant protein synthesized by type II pneumocytes and stored in lamellar bodies)^31^, showed similar intracellular morphology and size of lamellar bodies in the vehicle and GNE-7915-treated mouse lungs (Fig. 7b).

**Fig. 8:**
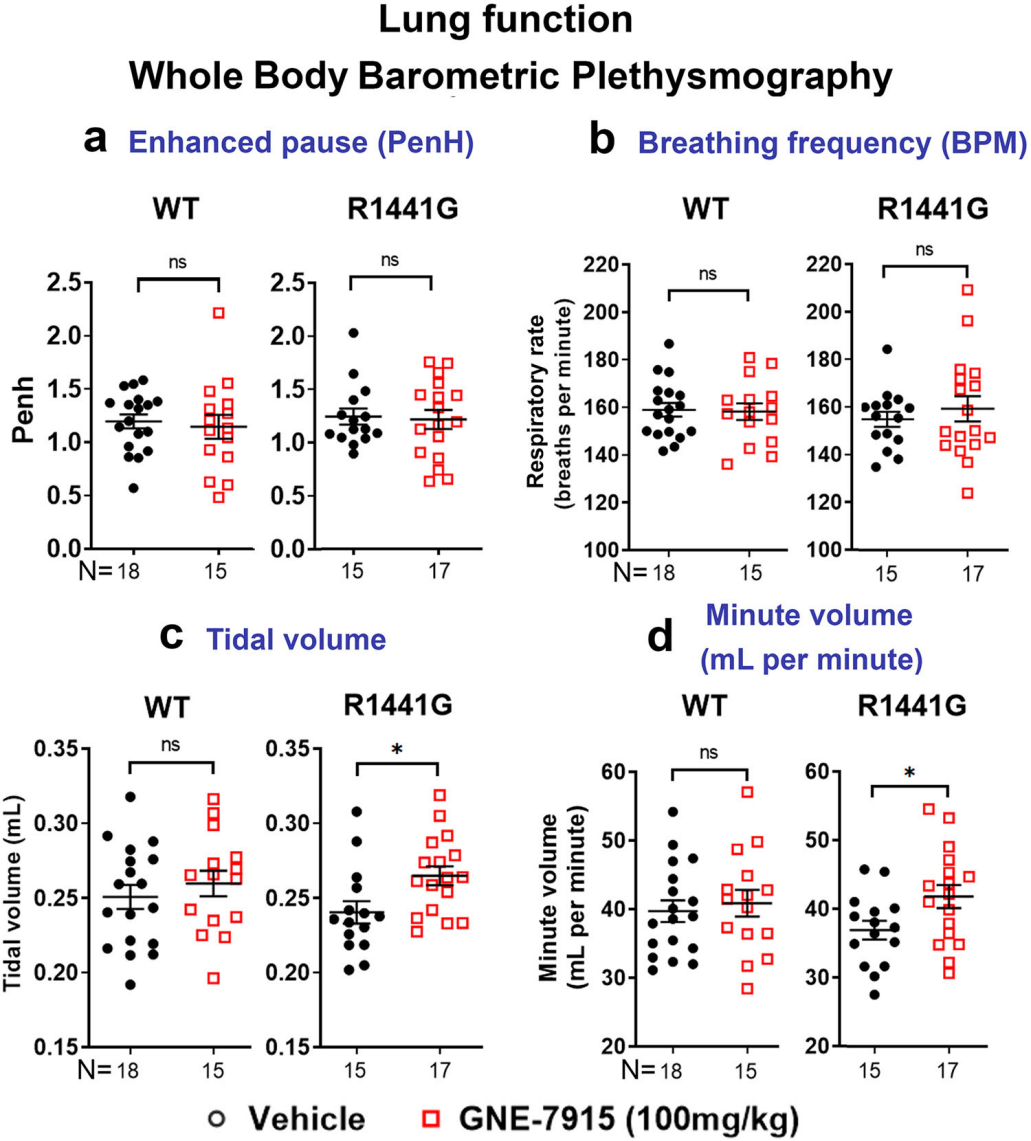
Lung function of mice after long-term GNE-7915 treatment using whole-body barometric plethysmography (WBBP). Readings were taken in conscious, unstrained mice at 24 h after final injection at the end of GNE-7915 treatment regimen. **a**–**d** No significant changes in Penh index in all treatment groups. Data are expressed as mean ± SEM. *X*-axis numbers represent total number of animals in each treatment group. Datasets were subjected to D’Agostino & Pearson normality test prior to statistical analysis. Statistical comparison between two designated groups for drug effects was performed independently in WT and LRRK2 mutant mice by unpaired, parametric Student’s *t*-test. *p < 0.05 represents independent statistical comparison between two designated groups. “ns” not significant.

In addition to histological examination of the lungs, the respiratory function was evaluated non-invasively by whole-body barometric plethysmography. Measurements were taken 24 h after the final injection. There were no significant changes in Penh (an empirical dimensionless index of breathing pattern of animals)^32^ in all treatment groups in both WT and mutant mice (Fig. 8a). Breath frequency (Fig. 8b), tidal volume (Fig. 8c) and minute volume (Fig. 8d) were similar between vehicle-treated WT and mutant mice, although a slight but significant increase in tidal volume and minute volume was observed in mutant mice after GNE-7915 (*p* < 0.05) (Fig. 8c, d). Long-term GNE-7915 did not cause any abnormal changes in lung gross morphology, histology or function in both WT and mutant mice.

### No gross abnormal renal morphology or histology after long-term GNE-7915

**Fig. 9:**
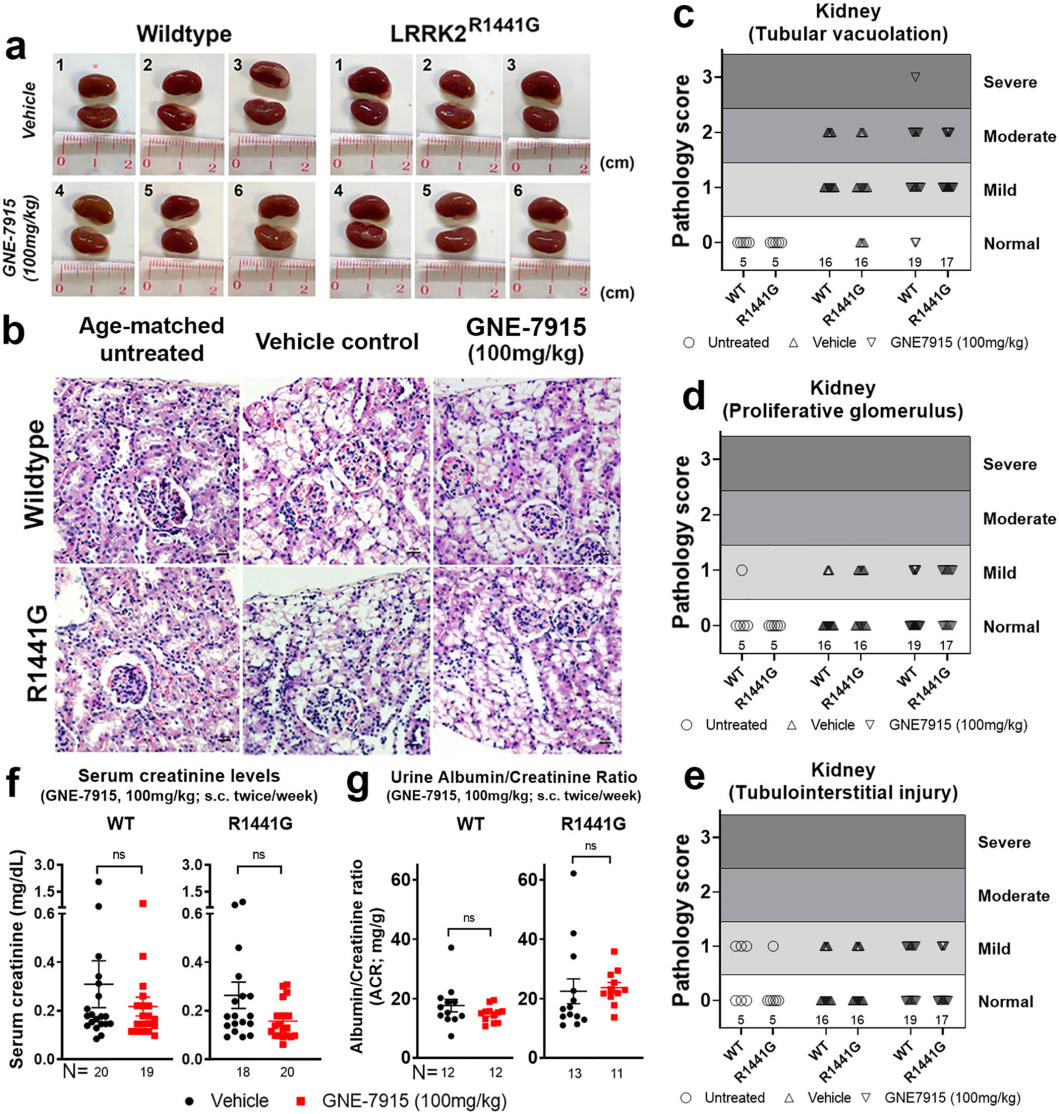
Kidney gross morphology in WT and LRRK2^R1441G^ mutant mice after long-term treatment. **a**, **b** No observable differences in gross renal morphology in WT and mutant mice compared to vehicle-treated groups; **c**–**e** Renal histopathology scores after vehicle and GNE-7915 treatment in WT and mutant mice; **f** No significant differences in serum creatinine levels, and **g** urinary albumin-to-creatinine (ACR) ratios in WT and mutant mice. *X*-axis numbers represent total number of animals in each treatment group. Data are expressed as mean ± SEM. Statistical comparison between two designated groups for drug effects was performed independently in WT and LRRK2 mutant mice by unpaired, Mann–Whitney U-test, a non-parametric test because of unequal variances. “ns” not significant.

We examined kidneys for adverse effects after GNE-7915 treatment. Both kidneys from GNE-7915- and vehicle-treated mice were harvested 24 h after the final injection. Kidney gross morphology in both WT and mutant mice appeared normal after treatment with vehicle or GNE-7915 (Fig. 9a, b). We examined renal histology and rated each individual mouse according to three commonly described pathology: (a) tubular vacuolation; (b) proliferative glomerulus; (c) tubulointerstitial injury. Severity level was defined as: “−” (not observed), “+” (mild), “++” (moderate), and “+++” (severe) (Supplementary Table 2). As the drug vehicle (i.e. 2-hydroxypropyl-β-cyclodextrin) could induce cytoplasmic vacuolation in renal tubule epithelium, we also identified any side effects of the vehicle by comparing them with age-matched WT mice without any treatment. Although mild to moderate tubular vacuolation and proliferative glomerulus were similarly found in both GNE-7915 and vehicle-treated WT and mutant mice, these abnormalities were not found in mice which had received no treatment whatsoever (Fig. 9c–e; Supplementary Table 2). These findings indicate that vacuolation and proliferative glomerulus in both vehicle-treated WT and mutant were due to long-term administration of the drug vehicle. Mice treated with GNE-7915 did not show any additional renal changes compared with changes seen with vehicle treatment (Fig. 9c–e). Furthermore, similar levels of tubulointerstitial injuries were observed in both GNE-7915 and vehicle-treated WT and mutant mice, indicating that our GNE-7915 treatment regimen did not cause any renal tubulointerstitial injuries (Fig. 9c–e).

### No abnormal urinary albumin-creatinine (ACR) ratio or serum creatinine levels after long-term GNE-7915

The kidney function of GNE-7915-treated mice was assessed by quantification of albumin and creatinine levels in one-time urine collected 24 h after the final injection. There were no differences in urinary ACR among all treatment groups, indicating kidney function was well preserved. Moreover, serum creatinine levels also showed no differences among all groups, which further confirmed that long-term GNE-7915 treatment did not cause adverse renal effects (Fig. 9f, g).

### No abnormal gross hepatic morphology or serum alanine aminotransferase (ALT) activity after long-term GNE-7915

**Fig. 10:**
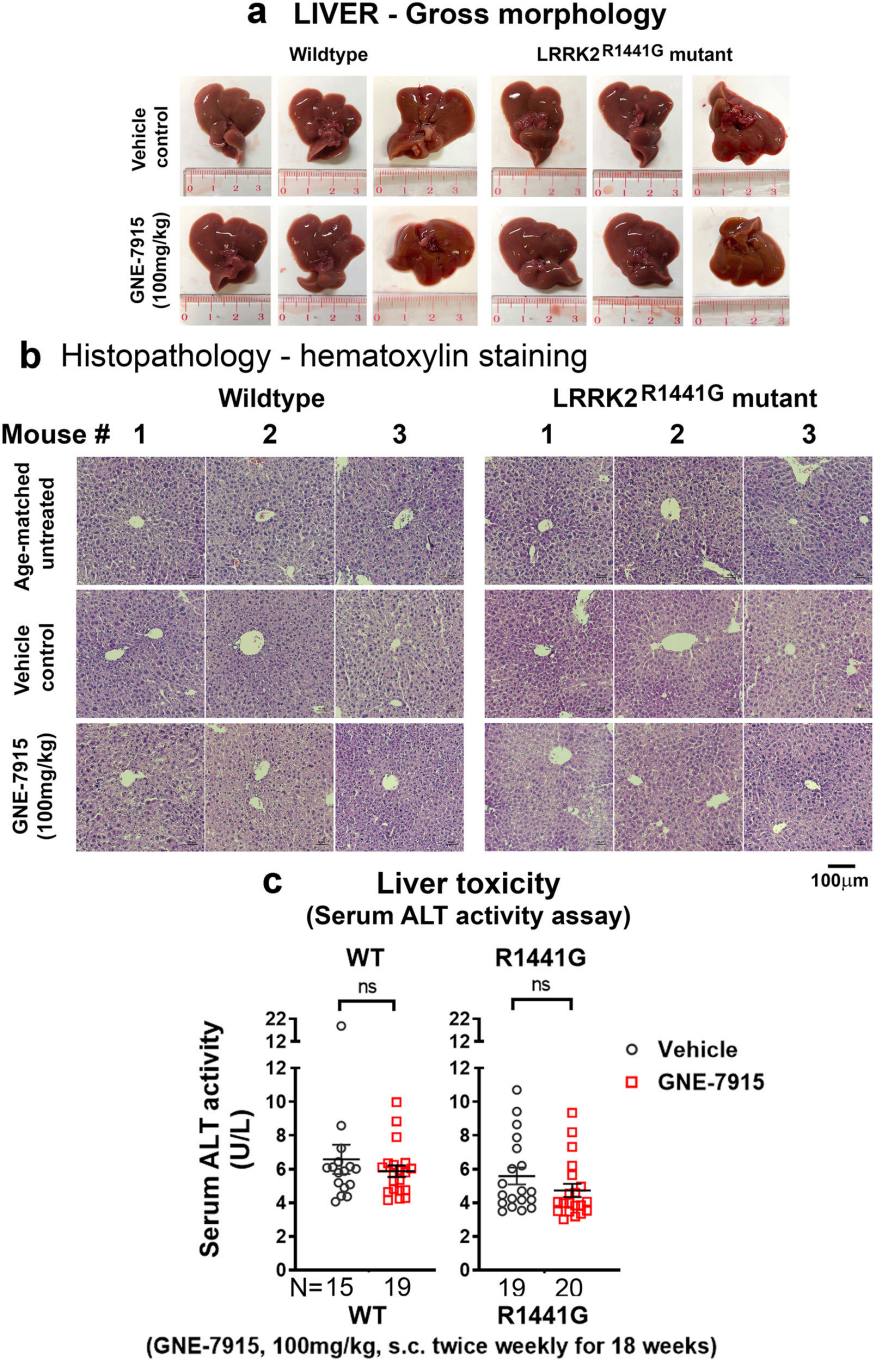
Hepatic morphology and function in GNE-7915 and vehicle-treated WT and mutant mice. **a**, **b** Whole liver showed no observable morphological changes after long-term GNE-7915 in WT and mutant mice. **c** Serum ALT levels were not significantly different between GNE-7915- and vehicle-treated mice. Data are expressed as mean ± SEM. Statistical comparison between two designated groups for drug effects was performed independently in WT and LRRK2 mutant mice by unpaired, Mann–Whitney U-test, a non-parametric test because of unequal variances. *X*-axis numbers represent total number of animals in each treatment group. “ns” not significant.

Elevated serum ALT activity is a marker of drug-induced liver damage^33^. To determine levels of hepatotoxicity, the whole liver was dissected 24 h after the final injection. Histological examination exhibited normal blood vessels and bile ducts without observable morphological differences after GNE-7915 compared with vehicle treatment in WT and mutant mice (Fig. 10a, b). Hepatocytes appeared normal after GNE-7915 compared with vehicle-treated controls (Fig. 10b). Serum ALT activity in all treated mice was similar, indicating no adverse effect on liver function after long-term GNE-7915 (Fig. 10c).

## Discussion

LRRK2 mutations represent one of the most common inherited risks in familial PD, and are present in about 1–3% of sporadic PD cases^12^. The most frequent pathogenic LRRK2 mutations are in G2019S, R1441C/G/H and Y1699C, all of which are associated with aberrant hyper-kinase activity^14^. In our LRRK2^R1441G^ knock-in mutant mice where the Roc-COR mutation is sited, the mutation does not directly confer hyperactive kinase activity per se but results in impaired GTP hydrolysis with consequent hyperactive kinase activity^14^. Increased LRRK2 kinase activity has been reported in post-mortem brains of idiopathic sporadic PD patients without LRRK2 mutation^13^, suggesting that LRRK2 gain-of-toxic-function may occur not only in LRRK2 mutants but also in sporadic PD. Here, we used an appropriate murine model to explore whether long-term inhibition of LRRK2 hyperactive kinase activity can be a promising disease-modifying therapeutic approach without causing observable adverse effects^1^.

The question arises whether our findings in mutant knock-in mice with C57/BL6 genetic background have relevance in humans. Although mouse models have their limitations, such as differences in lifespan, genetic sequence homology, neuroanatomical structure and the complexity of motor and non-motor behaviour, they have neuroanatomical similarities to humans^34^. Mouse's brain possesses a nigrostriatal system with dopaminergic pathways in which dopaminergic alterations are reflected in animal behaviour^34^. Moreover, the mouse genome has an overall 90% similarity to the human genome^35^, and a murine homolog of LRRK2 has a sequence up to 88% identical to that of human LRRK2 with conservation of PD-causing residues^36,37^. LRRK2 in humans and mice show similar expression patterns, as evidenced by the fact that LRRK2 is expressed in various organs in both species. The organs include the lung, heart, liver and kidney, and at a lower level throughout the brain in both humans and mice^38–41^. With its ~2-year lifespan and its ability to give rise to a reasonable number of offspring, the mouse allows a sufficient sample size for experimentation with the opportunity to introduce an ageing factor, which is important in PD. In our study, we attempted to mimic a long-term (equivalent to ~13 human years) treatment regimen to our 14-month-old LRRK2 mutant knock-in mice, which we had previously shown to have a greater accumulation of brain αSyn oligomers with age compared with that in their WT littermates^7^.

Similar to LRRK2-associated PD cases in humans, LRRK2^R1441G^ knock-in mice have a single base substitution resulting in non-synonymous R1441G mutation in the LRRK2 Ras-of-complex (Roc) GTPase domain^21^. The mutated *Lrrk2* gene is located at the same locus as that of WT, sharing similar gene regulation and expression profiles. LRRK2 mutant knock-in mice closely mimic human mutant LRRK2 carriers who have similar LRRK2 expression levels as those without the mutation^21^. Although most mutant LRRK2 human carriers are heterozygous, we used the homozygous knock-in mouse model to accentuate the genetic risk. Consistent with other LRRK2 knock-in mice carrying different mutations, our R1441G mutant mice do not exhibit obvious motor phenotypes. However, they exhibit subtle but significant PD-related phenotypes. For example, they are more susceptible to reserpine-induced dopamine depletion and synaptic dysfunction with a slower recovery following the exposure^21^. These mutant mice are also more hyperactive in open field behavioural tests consistent with anxiety, a prodromal feature of PD^42^. More recently, we showed that our mutant mice exhibited aberrant mitochondrial morphology and dysfunction in striatal neurons, which were associated with impaired mitophagy^43^. These subtle changes recapitulate some of the earliest pathogenic features of PD. More importantly, our mutant mice consistently show greater age-related accumulation of αSyn oligomers in the striatum and cortex compared with that in WT controls, due in part to impaired chaperone-mediated autophagy (CMA)^7^.

The current study is aimed at establishing a feasible, long-term dosing regimen using a specific brain-penetrant LRRK2 inhibitor which is efficacious in reducing brain αSyn oligomers without causing excessive kinase inhibition and adverse effects in peripheral tissues. A number of known LRRK2 inhibitors are available^1^. From these, we tested GNE-7915 which is metabolically stable and brain-penetrant [reported CSF/plasma_unbound_ ratio = 0.6 in rat (0.5 mg/kg; i.v.)]^44^. This drug has a calculated in vivo brain IC_50_ of 7 nM in mice^44^. It achieved high concentrations in peripheral tissues and brains in murine and non-human primate models and selectively inhibits LRRK2 kinase activity (IC_50_ = 9 nM in vitro). In our current study, LC-MS-MS demonstrated effective drug delivery to the brain, reaching a peak concentration of 500 ng/g of brain tissue at 1 h, which gradually decreased over 24 hours using our GNE-7915 treatment regimen at 100 mg/kg. Although earlier studies did not measure changes in phosphorylated Rab10 or Rab12 levels in the brain after GNE-7915 treatment, their reported AUC graph showed a concentration-dependent decrease in phosphorylated Ser1292-LRRK2 level (another marker of LRRK2 kinase activity) after intraperitoneal injections at both 10 and 50 mg/kg, with the predicted concentration in the brain at about 30 nM. Moreover, selectivity profiling using the DiscoverX KinomeScan competitive binding assay panel (392 kinases) demonstrated that LRRK2 had >65% probe binding, confirming GNE-7915’s selectivity to LRRK2. The possibility of non-specific kinase inhibition by GNE-7915 is relatively low^44^.

Initially, we determined the effects of LRRK2 kinase inhibition in the lungs of young mice after they had received a single subcutaneous injection of GNE-7915. The subcutaneous injection was chosen to ensure accurate dosing when the drug is given on a long-term basis. In considering suitable markers to assess the efficacy of GNE-7915 in reducing LRRK2 kinase activity, we found that pSer935- or pSer1292-LRRK2 levels were not useful because phosphorylation of both residues occurred at such low levels in LRRK2^R1441G^ mutant mice that further inhibition by GNE-7915 could not be accurately quantified. Similar observations were reported in LRRK2^R1441C^ knock-in mice from another independent study^45^. Hence, we used alternative markers of LRRK2 activity, namely pThr73-Rab10 (pRab10) and pSer106-Rab12 (pRab12)^25^. Although levels of pRab10 and pRab12 in lung correlate well with each other, as shown by correlation analysis between these two LRRK2 substrates in response to LRRK2 inhibitor treatment, we found that pRab10 expression was variable in the individual mouse brains, rendering it unreliable to reflect LRRK2 kinase activity in the brain. A similar observation was reported in another study using LRRK2^G2019S^ knock-in mice, which showed a lack of response in Rab10 phosphorylation to LRRK2 inhibition using MLi-2^27^. In contrast, pRab12 level was a more consistent marker of LRRK2 activity in the brain, which correlated well with LRRK2 inhibitor treatment and the corresponding changes in the lung. We found that Rab12 phosphorylation was maximally inhibited earlier by GNE-7915 at 6 h compared with Rab10, which was maximally inhibited at 24 h. Importantly, Rab10 and Rab12 are two independent LRRK2 substrates. Thus their relative levels of phosphorylation with respect to LRRK2 hyper-kinase activity may not be directly comparable, probably because of different kinetics of their phosphorylation reactions^46^. Therefore, it is unclear whether peripheral pRab10 and pRab12 levels can be used as surrogate biomarkers of brain LRRK2 kinase activity in human clinical trials.

Achieving efficacy in the human brain with minimal peripheral adverse effects from prolonged use of LRRK2 inhibitors in PD is challenging because different organs may have different susceptibility to LRRK2 inhibition. Drug delivery to the CNS is limited because of poorer penetration through the blood-brain barrier^47,48^. Therefore, it is reasonable that the effects of LRRK2 inhibition appear to be weaker in the brain than in peripheral tissues such as the lungs. When considering a suitable dosing regimen, our first approach was to assess the effect of a single injection of GNE-7915. LRRK2 inhibition, as determined by pRab10 and pRab12 levels, occurred in both WT and LRRK2 mutant lungs within 1 h, associated with a significant increase in GNE-7915 concentration in the serum, which reached a peak of ~9 µM. Maximal inhibition was observed between 6 and 24 h, with pRab10 and pRab12 levels being reduced by more than 50% of their basal levels. Subsequently, the kinase inhibition gradually dissipated, and LRRK2 kinase activity increased to baseline levels by 72 h. The interval required for the drug effects to dissipate determined the frequency of our subsequent injections in order to avoid excessive cumulative LRRK2 inhibition with long-term treatment. We used a twice-weekly dosing regimen to achieve a balance between excessive and possibly cumulative LRRK2 inhibitory effects and efficacy in the brain during the course of long-term treatment. A similar strategy of intermittent target inhibition had been adopted in an earlier clinical trial of tyrosine kinase inhibitors in the treatment of chronic myeloid leukaemia to preserve drug efficacy and reduce adverse effects^49^.

Excessive accumulation of misfolded αSyn aggregates in the brain beyond normal ageing is a pathological feature of PD^50^. Native αSyn exists in equilibrium amongst various forms, including oligomers, protofibrils and larger fibrillar conformations^51^. Aberrant accumulation of αSyn in PD exacerbates the formation of toxic αSyn oligomeric species that are released and propagated either as free-floating molecules or via extracellular vesicles in human brains^50,52^. αSyn oligomers perturb cellular membrane permeability and ion homeostasis, which subsequently trigger apoptotic cell death in PD^53,54^. In support of αSyn oligomers as a pathogenic species in PD, post-mortem studies reported elevated levels of αSyn oligomers in PD brains with Lewy body compared with levels in healthy controls^55,56^. Several ELISA-based studies also showed elevated levels of αSyn oligomers in cerebrospinal fluid (CSF) of PD patients^57^. More importantly, asymptomatic LRRK2 mutant carriers also exhibited increased αSyn oligomers in CSF, suggesting that αSyn oligomers may be a viable biomarker of prodromal PD^16^. Whilst there are no clinical biomarkers in routine use in PD which correlate with disease progression, accumulation of αSyn oligomers in the brain appears to be a useful marker of synucleinopathy in our LRRK2 mutant mice, which may potentially correlate with progression in the human disease^52^. Although LRRK2-associated PD cases may not show characteristic αSyn pathology such as Lewy bodies or neurites on autopsy, the current GNE-7915 regimen effectively inhibited LRRK2 kinase hyperactivity, which can potentially attenuate pathogenic events caused by aberrant LRRK2 hyperactivity, such as endo-lysosomal dysfunction^58^. Undoubtedly, an important index of drug efficacy in the treatment of PD appears to be the reduction of toxic αSyn oligomers in the brain. Our previous study established that striatal αSyn oligomer levels in our LRRK2 mutant mice significantly increased from the age of 15 months to 18 months compared to a much slower increase in age-matched WT controls^7^. This validates our aged LRRK2 mutant mice as an appropriate model to assess the therapeutic benefits of LRRK2 inhibition in PD. Previous studies suggested that αSyn toxicity may be associated with aberrant LRRK2 hyper-kinase activity in human^59,60^. Here, we have shown that 18-consecutive-week (equivalent to ~13 human years) treatment with GNE-7915 significantly reduced αSyn oligomer accumulation in mutant LRRK2 mouse striatum, a vulnerable brain region in PD. We are uncertain why the cortical αSyn oligomer level was not reduced by GNE-7915 treatment, unlike striatum. The sensitivity of our αSyn oligomer ELISA is up to 1.0 pg/ml, which does detect our current levels of oligomeric αSyn in both cortex and striatum. We believe that the lack of efficacy observed in the cortex was not due to a lack of the ELISA sensitivity but is likely to be due much lower absolute levels of αSyn oligomer in the cortex compared with that in the striatum in untreated WT and mutant mice (Fig. 3b). Nevertheless, our findings demonstrate the therapeutic potential of LRRK2 inhibitors to ameliorate synucleinopathy by reducing αSyn oligomer accumulation in ageing brains, a hitherto unreported fact. This finding is supported by the significant correlation between αSyn oligomer and pRab12 levels, confirming that the reduction of striatal αSyn oligomer levels was associated with the reduction of abnormally high LRRK2 activity in mutant animals. Our results are compatible with an earlier study that cerebral injection of LRRK2 antisense oligonucleotides to knockdown LRRK2 expression reduced pSer129-αSyn levels and the formation of αSyn inclusions in WT mouse brain that was exposed to αSyn pre-formed fibrils^61^.

The link between LRRK2 hyperactivity and aberrant accumulation of αSyn in PD brains is not entirely clear but appears to involve impaired autophagic mechanisms and aberrant phosphorylation of downstream substrates. Hyper-kinase activity of LRRK2 variants has been shown to affect endo-lysosomal transport and autophagy^62^. Previously we had shown abnormal lysosomal clustering and atypical accumulation of LAMP2A and HSPA8 in our aged LRRK2^R1441G^ mutant mouse brain, indicating impaired chaperone-mediated autophagy^7^. An earlier study showed that LRRK2 inhibitor [i.e. GSK2578215A; GlaxoSmithKline (GSK)] attenuated abnormal lysosomal morphology and function in the PD patient fibroblasts and mouse primary cultures carrying LRRK2-G2019S variant^63,64^. Although the mechanisms involved remain unclear, LRRK2 has been shown to phosphorylate various downstream substrates in endo-lysosomal trafficking and autophagic pathways, such as endophilin A1^65^, synaptojanin-1^66^ and the more recently discovered Rab-GTPases, including Rab3, Rab8, Rab10, Rab12, Rab35 and Rab43^25^. Rab-GTPases have been of particular interest, given that they are critical players orchestrating vesicle dynamics^67^. Amongst these substrates, Rab3a, Rab8 and Rab35 have been shown to interact with αSyn^18,68^. For instance, phosphorylation of Rab3a by mutant LRRK2 resulted in more intense interaction with αSyn, which promotes its embedment into membranes^69^. This membrane-bound αSyn has a high propensity to aggregate, which in turn become seeds for further aggregation, thus forming a vicious cycle once the process initiates^70^. Pathogenic LRRK2 variants may also impair lysosomal activity via downstream phosphorylation of Rab10 and Rab35, resulting in defective autophagosome transport^71^. Since αSyn oligomers are preferentially degraded via lysosomal pathway^72^, it is possible that the reduction of αSyn oligomers observed in our LRRK2 mutant mice after GNE-7915 treatment may involve modulation of downstream Rab signalling in the endo-lysosomal pathways.

Unlike in the mutants, our current GNE-7915 regimen appeared to have little or no effect on αSyn oligomers in aged WT normal brains, consistent with the lack of significant inhibition on Rab12 phosphorylation in WT mice treated with long-term GNE-7915. This could possibly be due to drug tolerance or compensation as a result of sustained LRRK2 inhibition below the normal physiological level. Furthermore, we found an unexpected increase in pSer129-αSyn levels in WT mice treated with GNE-7915. This raises concerns about whether therapeutic LRRK2 inhibition would be harmful to patients with normal LRRK2 activity. Phosphorylation of Ser129-αSyn enhances the propensity of the protein to form oligomers in vitro^73,74^. However, the elevation of pSer129-αSyn levels in WT mice after GNE-7915 treatment was not associated with an increase in αSyn oligomers or nigrostriatal dopaminergic cell death. It is unclear whether such a result is observed in other experimental models or in humans. Furthermore, clinical studies only showed a weak correlation between pSer129-αSyn levels in cerebrospinal fluid (CSF) and clinical disability scores (United Parkinson’s Disease Rating Scales: UPDRS) in PD patients^75^. Therefore, the lack of correlation between oligomer levels and Ser129-αSyn phosphorylation in our study is not surprising.

Having established an effective treatment regimen to reduce αSyn oligomers in the brain, we assessed the risks of potential adverse effects on peripheral organs. The dose of any drugs acting on the CNS is likely to be partitioned between peripheral tissues and the brain, with the former receiving the greater proportion^76^. LRRK2 is involved in diverse cellular processes, including vesicular trafficking, cytoskeleton dynamics, autophagy, neurotransmission, mitochondrial function and immune responses^1^. It is possible that the amount of LRRK2 inhibitor required to achieve brain efficacy could also induce adverse effects from excessive kinase inhibition in peripheral tissues which express LRRK2, such as lung and kidney^77^. In particular, LRRK2 protein expression in the lung is higher than that in the brain^78^, although its role in normal lung function and pathophysiology remains unclear^79^. Studies revealed that LRRK2 inhibition by twice daily oral dosing of GNE-7915 (30 mg/kg) for 2 weeks in non-human primates (cynomolgus monkeys) resulted in abnormal cytoplasmic vacuolation and enlarged lamellar bodies in type II pneumocytes although these abnormalities were reversible after cessation of the drug^20,38^. Moreover, a 15-week in-diet treatment regimen of another brain-penetrant LRRK2 inhibitor, MLi-2, in young 20-week-old mice also caused enlargement of alveolar cells^30^. Conversely, another study using oral GNE-7915 given twice daily at 200 or 300 mg/kg for 15 days in mice did not show severe pathology in the lung and kidney^38^. These divergent findings appear not only to be due to species differences in drug disposition but also to the dosage, frequency and duration of treatment. Although our GNE-7915 treatment was given twice weekly over 18 consecutive weeks, which is much longer than any other reported regimen in the literature, our regimen was well tolerated by both our WT and mutant mice without abnormal body weight loss and signs of health problems. Furthermore, we also assessed whether chronic LRRK2 inhibition caused harm to the lung. We conducted a detailed examination of the lungs from our GNE-7915-treated animals and found no observable abnormalities in gross morphology, size and colour. Cell morphology and distribution of type II pneumocytes in the lungs as visualized by immunostaining of the marker protein SP-C did not show any observable differences after GNE-7915 treatment. Moreover, we also specifically examined the size and intracellular morphology of lamellar bodies in pneumocytes after GNE-7915 treatment by transmission electron microscopy (TEM) and immunostaining of SP-B, respectively. SP-B is a surfactant protein stored in lamellar bodies, which is a useful marker to visualize intracellular pattern of lamellar bodies. Although several reports showed abnormal enlargement of lamellar bodies following LRRK2 inhibition^20,80^ or after knockout of the Lrrk2 gene^81,82^, we did not find any observable differences in both the size and morphology of the lamellar bodies of pneumocytes in our GNE-7915-treated mice compared to the vehicle-treated controls. Although the reason why LRRK2 inhibition causes enlargement of lamellar bodies in these pneumocytes is unclear, the lack of such abnormality in our GNE-7915-treated mice may be attributed to our dosing regimen that did not cause excessive LRRK2 inhibition. Respiratory function in these mice was normal and comparable with that of vehicle-treated mice, confirming that lung function was not affected by the long-term drug treatment, consistent with the histology and TEM results. Our finding is compatible with reports of reduced LRRK2 activity in humans with a heterozygous loss of function (LOF) variant resulting in 50% decrease in LRRK2 protein expression, which had no discernible effect on survival or health^83^.

As in the lung, the kidney also expresses LRRK2 at a higher level than that in the brain. Early pathophysiological changes, including darkening of kidneys and microvacuolation in proximal tubular cells, were observed in LRRK2 knockout and kinase-dead mice^77^, suggesting that LRRK2 activity is crucial to kidney homeostasis. We assessed whether long-term LRRK2 inhibition by GNE-7915 caused renal effects, including changes in kidney gross morphology, serum creatinine levels, and urinary ACR (a marker of early renal diseases or diabetic nephropathy). Gross morphology in terms of organ size and colour appeared normal in both GNE-7915-treated WT and mutant mice, consistent with earlier studies that showed no abnormal kidney phenotypes in LRRK2^G2019S^ mutant mice^77^. Moreover, serum creatinine levels and ACR also appeared normal in our GNE-7915-treated mice, indicating that renal function was preserved. We noted a similar histological change of renal cortical tubular vacuolation in both vehicle- and GNE-7915-treated WT and mutant mice that were not seen in age-matched untreated animals. Proliferative glomeruli (including the proliferation of glomerular cells or infiltration of leukocytes) were also evident in both vehicle- and GNE-7915-treated mice compared to untreated animals. Such observations led us to examine the effects of the drug vehicle on kidney histology. We used 2-hydroxylpropyl-β-cyclodextrin (β-CD) as a drug vehicle for GNE-7915, with better water solubility and less toxicity compared to alternative cyclodextrins (CDs)^84^. Administration of CDs to rats can increase apical vacuoles and lysosomes in the renal tubular epithelium that was reversible upon its cessation^85^. Such vacuolation is considered a physiological response of renal sequestration of materials which does not appear to compromise renal function^86^. We attributed this as side effects from long-term administration of β-CD as a drug vehicle rather than GNE-7915 per se, which did not cause functional damages. Furthermore, tubulointerstitial injuries in all groups of mice were similar and comparable, indicating that these were not related to GNE-7915 treatment.

LRRK2 is highly expressed in circulating and tissue immune cells^29^. Polymorphisms in LRRK2 have been linked to various immune diseases^29^. This raises concerns about whether chronic LRRK2 inhibition would have adverse effects on the immune system. An earlier study had shown that LRRK2 deficient mice exhibited hyperactive immune response and increased inflammation in lung^87^, indicating that complete ablation or excessive inhibition of LRRK2 may cause adverse effects to the immune system similar to other peripheral tissues with high LRRK2 expression. IL-6 is a cytokine secreted by innate immune cells in response to inflammation and autoimmunity^88^ which is a well-established marker of infection or inflammation^89^. Here, we measured serum IL-6 levels in WT and LRRK2 mutant mice after GNE-7915 treatment. We found that circulatory IL-6 levels were similar between WT and LRRK2 mutant control mice with vehicle treatment, suggesting that LRRK2 mutation per se does not appear to cause host tissue injuries or immune cell activation. We also found no significant difference in serum IL-6 levels between WT and mutant mice after long-term GNE-7915 treatment indicating an absence of infection or inflammation. However, it is noteworthy that all our mice were bred in a clean and relatively sterile environment within our animal facility, without any exposure to potential pathogens or environmental toxins that can trigger immune responses whilst under LRRK2 kinase inhibition. Whether long-term LRRK2 inhibition can trigger inflammatory responses or cause susceptibility to infections in humans warrants further controlled studies.

It is reassuring the completed Phase 1 clinical trials in healthy volunteers to study the safety and tolerability of oral LRRK2 inhibitors have yielded promising results^1^. Although the results of global clinical trials on mild to moderate sporadic and LRRK2-PD patients are yet to be announced (ClinicalTrials.gov Identifier: NCT04056689; NCT03710707; NCT03976349), our study showed that kinase inhibition using twice-weekly subcutaneous GNE-7915 regimen in our LRRK2 mutant knock-in mice over 18 consecutive weeks (equivalent to ~13 years in human) was well tolerated and did not cause any apparent physical abnormalities or increased mortality. We did not find any evidence of systemic inflammation, lung, kidney or liver toxicity in both WT and mutant mice related to GNE-7915 treatment. It is unclear whether histopathological changes in peripheral organs from LRRK2 inhibition may have adverse clinical consequences. The impact of long-term LRRK2 inhibition, especially in those individuals with normal LRRK2 activity, also remains unclear. Although LRRK2 KO animals may not necessarily show any major abnormalities^90^, this situation is different from therapeutic LRRK2 inhibition in individuals with normal LRRK2 activity since birth because there could be compensatory mechanisms in KO animals to obviate the effects of low endogenous LRRK2 expression^91^. Nevertheless, we have demonstrated that long-term, intermittent LRRK2 inhibition, which restored hyperactive kinase activity in mutant mice to physiological levels, significantly reduced the accumulation of αSyn oligomers in the brain. The therapeutic benefits of LRRK2 inhibition in the brain without causing peripheral adverse effects come from reducing excessive kinase activity in mutant cells to normal levels but not significantly below them. Pre-clinical assessment for LRRK2 activity from accessible peripheral tissues from PD patients may be useful to minimize adverse effects from the overdose of LRRK2 inhibitor whilst maintaining efficacy in brain^1^. Long-term LRRK2 inhibition based on an appropriate dosing regimen can be efficacious and safe in attenuating synucleinopathy in PD.

## Methods

### Mutant LRRK2^R1441G^ knock-in mouse model

A C57BL/6 mouse colony with homozygous knock-in of LRRK2^R1441G^ mutation were back-crossed with wild-type C57BL/6 for over eight generations to maintain a pure genetic background^21^. All mice were maintained with unrestricted access to food and water on a 12 h light-dark cycle, with lights on at 7 AM in the Laboratory Animal Unit, University of Hong Kong (HKU), with accreditation through the Association for Assessment and Accreditation of Laboratory Animal Care (AAALAC), under standard conditions (12 h light/dark cycle). All experimental protocols were approved by the Committee on the Use of Live Animals in Teaching and Research (CULATR) of HKU.

### Generation and culture of mouse embryonic fibroblasts (MEFs)

Littermate-matched WT and LRRK2^R1441G^ mutant mouse embryonic fibroblasts (MEFs) were isolated from embryos at day E12.5 resulting from crosses between heterozygous LRRK2^R1441G/WT^ mice^7^. All MEFs were maintained under normal culture conditions as described previously^7^. The genotype of MEF clones was confirmed by Sanger sequencing.

### Overexpression of human αSyn in SH-SY5Y cells and LRRK2 inhibitor (GNE-7915) treatment

To assess the effect of GNE-7915 on αSyn phosphorylation, human αSyn protein was stably overexpressed in SH-SY5Y cells (ATCC; CRL-2266) by transduction of lentivirus. Briefly, the human SNCA (NCBI Entrez Gene: 6622) gene was amplified by PCR from the total cDNA of SH-SY5Y cells as a template to generate an insert of cDNA fragment encoding for *h*SNCA conjugated with an 18-amino acid protein tag. This *h*SNCA-NE cDNA insert was then sub-cloned into a lentiviral backbone plasmid *pSIN4-EF2-IRES-Pur* derived from a gift (Addgene™ plasmid, 16580)^92^ to construct *pSIN4-hSNCA-NE* plasmid (Addgene™ plasmid, 102366). ‘NE’ is a novel 18-amino-acid epitope tag we developed to facilitate specific protein detection by a monoclonal NE antibody^7^ (Versitech Ltd. Hong Kong; http://www.versitech.hku.hk/reagents/ne/). To generate lentivirus, *pSIN4-hSNCA-NE* plasmid was co-transfected with helper plasmids in 293 T cells (ATCC; CRL-3216) using Lipofectamine3000™ (ThermoFisher™ Scientific, L3000-015) for 3 days. Virus-containing media were collected and filtered before transduction of SH-SY5Y cells to induce overexpression of αSyn protein. The expression of total and phosphorylated Ser129-αSyn in these cells was confirmed by Western blots and immunocytochemistry before treatment. Cells at 80% confluence were treated with GNE-7915 ([4-[[4-(ethylamino)-5-(trifluoromethyl)-2-pyrimidinyl]amino]-2-fluoro-5-methoxyphenyl]-4-morpholinyl-methanone; CAS#:1351761-44-8; Cayman™ Chemical), at a dose range from 0.1 to 100 nM for 24 h before total cell lysates were extracted for western blot analyses. Negative control cells were treated with DMSO (0.01%).

### Duration and effectiveness of single subcutaneous injection of GNE-7915 in mice

Drug efficacy and duration of GNE-7915 were determined in young 3-month-old WT and LRRK2^R1441G^ mutant mice by a single subcutaneous (s.c.) injection of GNE-7915 or drug vehicle (2-hydroxypropyl-β-cyclodextrin; 40% w/v). Mice treated with either GNE-7915 or vehicle were sacrificed at 1, 6, 24, 48 or 72 h after injection. Their lung and brain tissues were collected for western analyses of phosphorylated Thr73-Rab10 (pRab10) and phosphorylated Ser106-Rab12 (pRab12) levels to determine the relative LRRK2 kinase activity. GNE-7915-mediated LRRK2 inhibition in the lung and brain was calculated by the percentage reduction of pRab10 and pRab12 by GNE-7915 at different time points.

### Long-term GNE-7915 treatment regimen

GNE-7915 was freshly dissolved in the drug vehicle (2-hydroxypropyl-β-cyclodextrin; 40% w/v) at a stock concentration of 10 mg of GNE-7915 per mL of drug vehicle. Fourteen-month-old male WT and LRRK2^R1441G^ mutant mice from different litters were randomly assigned to receive GNE-7915 (100 mg/kg; s.c.) or a drug vehicle twice weekly for 18 consecutive weeks. The body weight of each mouse was recorded weekly during that treatment period. The maximum volume of drug suspension per subcutaneous injection was 200 µl.

### Detection and quantification of GNE-7915 using LC-MS-MS

To determine concentrations of GNE-7915, we quantified the drug in mouse serum and brain using LC-MS-MS. Briefly, 14-month-old mice were randomly divided into four different groups, which were euthanized at four time points (i.e, 0, 1, 6 or 24 h after a single subcutaneous injection of GNE-7915 (*N* = 3 per group). Mouse sera were obtained by centrifuging the blood at 3500 × *g* for 15 min at room temperature. Both serum and whole brain were snap-frozen in liquid nitrogen immediately after collection. For drug extraction, serum and brain samples were homogenized in 4 ml of acetonitrile. GNE-7915 in serum and brain samples was extracted by sonicating the homogenized samples for 20 and 40 min, respectively. The samples were then centrifuged at 10,000 rpm for 10 min to remove insoluble tissue debris. The supernatant was deproteinized and delipidated twice by the addition of 2 ml of acetonitrile and hexane, respectively. The clean acetonitrile layer was obtained after centrifugation at 10,000 rpm for 10 min. GNE-7915 in the acetonitrile extract was analyzed using an Agilent (Palo Alto, CA) 1290 Infinity liquid chromatograph coupled to a SCIEX QTRAP 3200 tandem mass spectrometer (Woodlands, Singapore) with an electrospray ionization interface. The analytical column is ZORBAX Eclipse Plus C18 (2.1 × 100 mm, 1.8 μm, Agilent) equipped with its corresponding guard column (5 × 2.1 mm, 1.8 µm, Agilent). The elution was conducted under isocratic conditions with a mobile phase composed of 90% methanol (with 10 mM ammonium acetate) and 10% Milli-Q water.

### Specimen collection and preparation

Mice were deeply anaesthetized with pentobarbital before sacrifice. Blood was collected via cardiac puncture and was allowed to clot for 30 min undisturbed at room temperature before serum collection by centrifugation at 2000 × *g* for 10 min. One-time urine was collected directly from the bladder for albumin and creatinine assays. For the preparation of tissue lysates for ELISA and western blotting, different regions of the mouse brain and peripheral organs, including lung, kidney and liver, were freshly dissected and snap-frozen in liquid nitrogen after collection before immunoblotting analysis and ELISA. Serum was collected for IL-6 levels prior to sacrifice and quantified by ELISA to detect any evidence of inflammation or infection.

### Quantification of α-synuclein oligomers

αSyn oligomer levels in brain lysates were assessed by a mouse αSyn oligomer ELISA kit (MBS724099; MyBioSource.com™). Briefly, brain tissues were freshly homogenized in ice-cooled PBS supplemented with Halt™ Protease and Phosphatase Inhibitor (ThermoFisher™ Scientific, 78444), PMSF (Pierce™, #36978) and EDTA to preserve the native conformation of αSyn oligomer. Homogenates were briefly centrifuged for 5 min at 720 × g to remove fatty tissues and nuclei. Resultant supernatants containing cytoplasmic fraction and small cellular organelles were further clarified by centrifugation at 4 °C for 15 min at 12,000 × *g*. Cytosolic fractions containing soluble αSyn oligomers were collected from the resultant supernatants, whereas insoluble membranous fractions were resuspended and washed twice in cold PBS before being completely dissolved in RIPA lysis buffer with 0.1% SDS (Abcam™, ab156034) for further analysis by Western blotting. Protein concentrations of PBS-soluble lysates were determined by Bradford assay (ThermoFisher™ Scientific, #5000205). Briefly, 500–700 µg of total lysates were subjected to αSyn oligomer ELISA according to the manufacturer’s protocol. Quantification of total soluble αSyn oligomer levels in PBS-soluble lysates (pg oligomers/mg total protein) was based on the linear standard curve generated from recombinant αSyn oligomer standards provided by the kit. To validate the specificity of this αSyn oligomer ELISA, total cell lysates of LRRK2^R1441G^ mutant mouse embryonic fibroblasts (which do not express α-synuclein), and a commercially available purified recombinant monomeric αSyn protein (Abcam™; #ab218818) were used as two independent negative controls. Both MEF total lysates and recombinant monomeric α-synuclein protein did not produce a signal in this oligomeric αSyn ELISA (Supplementary Fig. 3).

### Cellular αSyn clearance assay

To determine the rate of αSyn clearance, LRRK2 mutant MEFs stably expressing *PAmCherry-SNCA-NE* were photoactivated under UV-A (405 nm) according to our previous protocol^7^. The presence of red fluorescence emitted by photoactivated PAmCherry was confirmed under microscope. Initial red fluorescence level (t = 0) and levels in each group at 6, 24, 48 h post-photoactivation were measured by flow cytometry (Texas-red channel). The rate of αSyn clearance in mutant MEFs, with and without GNE-7915 treatment, was determined as a total fluorescent signal compared with corresponding initial levels of PAmCherry at t = 0. Presence of newly synthesized PAmCherry proteins (non-fluorescent) post-photoactivation would not confound the flow cytometry protein clearance assay.

### Assessment of lung histology and function

Basal lung function of WT and mutant LRRK2 mice was recorded after the last injection of GNE-7915 or drug vehicle. The respiratory function of mice was measured non-invasively by barometric whole-body plethysmography (WBP; DSI™, St. Paul, MN, USA). After acclimating the mice in the chambers, readings were taken one day after the final GNE-7915 injection. The resulting box pressure signals caused by volume and pressure changes during the respiratory cycle of the animal were used to calculate the tidal volumes, minute ventilation, and enhanced pause (PenH). The morphology of type II pneumocytes was assessed by immunostaining using a specific marker, surfactant protein C (SP-C) (Abcam™, ab90716) in PFA-fixed lung sections of the mice after counterstained by hematoxylin.

### Transmission electron microscopy (TEM)

Mouse lung tissue sections for TEM were prepared from the paraffin-embedded tissue blocks of WT and LRRK2 mutant mice which were freshly dissected and fixed with 4% paraformaldehyde in PBS with reference to literature^93^. Briefly, a tissue block was cut from paraffin block using a scalpel into 1 mm^2^ cubes, and subsequently dewaxed by xylene. Then, these tissue blocks were dehydrated in ethanol and incubated in 2% osmium tetroxide at 4 °C for 60 min. After dehydration in a series of graded ethanol and propylene oxide solutions, tissues were embedded in epoxy resin and sectioned on nickel grids. The resin-embedded lung tissue sections were examined under TEM (Philips EM208s transmission electron microscope) at a magnification of ×6600.

### Alanine aminotransferase (ALT) activity assay for hepatotoxicity

Potential drug-induced hepatotoxicity was assessed by ALT activity assay (#MAK052; Sigma) in serum collected from mice after 18 weeks of GNE-7015 treatment. Serum ALT level was used as a marker for drug-induced liver injury. ALT activity is determined by a coupled enzyme assay, which results in a colorimetric (570 nm)/fluorometric (λ_ex_ = 535/λ_em_ = 587 nm) product, proportional to the pyruvate generated. One unit of ALT is defined as the amount of enzyme that generates 1.0 μmole of pyruvate per min. at 37 °C.

### Interleukin-6 (IL-6) assay

IL-6 is a circulating, pro-inflammatory cytokine which is increased in inflammation and infection^88^. Serum IL-6 levels were assessed by mouse IL-6 ELISA (ThermoFisher Scientific™; KMC0061) according to the manufacturer’s protocol. The analytical sensitivity is <3 pg/ml mouse IL-6. Its concentrations (pg/ml) were calculated using the standard curve generated using mouse IL-6 provided by the kit.

### Urinary albumin-creatinine ratio (ACR) and serum creatinine levels

Urine albumin level after long-term GNE-7915 was determined by mouse albumin ELISA quantification kit (ab108792; Abcam) according to the manufacturer’s protocol. Similarly, serum creatinine was quantified by a mouse creatinine assay kit (80350; Crystal Chem™) according to the manufacturer’s protocol.

### Immunohistochemistry

For tissue histology, mice were anaesthetized with pentobarbital and then perfused with trans-cardiac cold PBS followed by 4% paraformaldehyde (PFA). Brain, lung, kidney and liver were dissected and post-fixed at 4 °C overnight. Following dehydration and embedding in paraffin wax, sample tissues were coronally sectioned at 8 µm thickness. After antigen retrieval, tyrosine hydroxylase (TH) (1:500; Millipore #AB318), phospho-Ser129 alpha-synuclein (1:200; Abcam™, ab184764) and phospho-Ser106-Rab12 (1:200; Abcam™, ab256487), were used as primary antibodies for incubation at 4 °C overnight. Sections were subsequently incubated with HRP-conjugated secondary antibodies (DAKO, P0488 and P0260), followed by TMB substrate (DAKO, K3468) for colour development. Positive immuno-stained slides were examined and photographed under a light microscope.

### Western blotting

Cells or tissue lysates in standard RIPA buffer were mixed with SDS-PAGE sample loading buffer (62.5 mM Tris, pH 6.8, 100 mM DTT, 2% SDS, 10% glycerol, 0.002% bromophenol blue) and heated at 75 °C for 10 min. Samples (15–40 µg) were electrophoresed in 12% SDS-polyacrylamide gels and transferred to PVDF membranes. Membranes were blocked with 5% (w/v) Bovine Serum Albumin (BSA) (Sigma, A9647) in TBS-T (ChemCruz, sc-3362311). and probed with primary antibodies, followed by HRP-conjugated secondary antibodies (DAKO, P0448 and P0260). Membranes were probed with ECL solution (Bio-Rad, #170-5061) for substrate detection. The intensity of the various protein bands was quantified using ImageJ software (http://rsbweb.nih.gov/ij/plugins/track/track.html). All Western blots containing comparable groups were processed in parallel and derived from the same experiment.

The following antibodies were used: Phospho-Ser935 LRRK2 (1:1000; Abcam™, ab133450), total LRRK2 (1:1000; Abcam™, ab133474), phospho-Thr73-Rab10 (1:2000; Abcam™, ab230261), total Rab10 (1:1000; Cell Signaling Technology™, #8127 S), phospho-Ser106-Rab12 (1:1000; Abcam™, ab256487), total Rab12 (1:1000; MRC-PPU Reagents and Services, SA227), phospho-Ser129 alpha-synuclein (1:3000; Fujifilm WAKO Pure Chemical Corporation, pSyn#64), total alpha-synuclein (1:3000; Abcam™, ab1903) and beta-actin (1:5000; Cell Signaling Technology™, 5125 S).

### Statistical analyses

All experiments were performed based on a sufficient number of independent trials to achieve statistical significance, as indicated in figure legends. Results were expressed as means ± SEM. Conclusions were drawn based on statistical analyses using GraphPad™ PRISM software (GraphPad Inc., CA). The normality of data sets was determined using D’Agostino & Pearson omnibus normality test. Potential outliers were identified using Grubb’s test^94^. Statistical comparisons involving interaction between genotype and GNE-7915 drug effect were determined by two-way ANOVA with post hoc Tukey’s multiple comparisons. Differences between the vehicle- and GNE-7915-treatment groups were determined by unpaired, parametric Student’s *t*-test to demonstrate drug effect independently in each of the genotype groups. An alternative analysis was performed using non-parametric, Mann–Whitney U-test to compare differences between two independent groups when the dependent variable is not normally distributed. *p*-values less than 0.05 (*p* < 0.05) were considered statistically significant.

## Supplementary information

Supplementary Information

## Data Availability

There are no data in this paper that can be submitted to a data repository. The data that support the findings in this article are available from the corresponding author on request.

## References

[1] Tolosa E, Vila M, Klein C, Rascol O (2020). LRRK2 in Parkinson disease: challenges of clinical trials. Nat. Rev. Neurol..

[2] Pang SY-Y (2019). The interplay of aging, genetics and environmental factors in the pathogenesis of Parkinson’s disease. Transl. Neurodegener..

[3] Bandres-Ciga S, Diez-Fairen M, Kim JJ, Singleton AB (2020). Genetics of Parkinson’s disease: an introspection of its journey towards precision medicine. Neurobiol. Dis..

[4] Huang M (2019). α-Synuclein: a multifunctional player in exocytosis, endocytosis, and vesicle recycling. Front. Neurosci..

[5] Lashuel HA, Overk CR, Oueslati A, Masliah E (2013). The many faces of alpha-synuclein: from structure and toxicity to therapeutic target. Nat. Rev. Neurosci..

[6] Spillantini MG, Crowther RA, Jakes R, Hasegawa M, Goedert M (1998). α-Synuclein in filamentous inclusions of lewy bodies from parkinson’s disease and dementia with Lewy Bodies. Proc. Natl Acad. Sci. USA.

[7] Ho PW-L (2020). Age-dependent accumulation of oligomeric SNCA/α-synuclein from impaired degradation in mutant LRRK2 knockin mouse model of Parkinson disease: role for therapeutic activation of chaperone-mediated autophagy (CMA). Autophagy.

[8] Rodriguez L, Marano MM, Tandon A (2018). Import and export of misfolded α-synuclein. Front. Neurosci..

[9] Singleton AB (2003). α-Synuclein locus triplication causes Parkinson’s disease. Science.

[10] Kayed R, Dettmer U, Lesné SE (2020). Soluble endogenous oligomeric α-synuclein species in neurodegenerative diseases: Expression, spreading, and cross-talk. J. Parkinsons Dis..

[11] Peelaerts W, Baekelandt V (2016). ɑ‐Synuclein strains and the variable pathologies of synucleinopathies. J. Neurochem..

[12] Healy DG (2008). Phenotype, genotype, and worldwide genetic penetrance of LRRK2 -associated Parkinson’s disease: a case-control study. Lancet Neurol..

[13] Kluss JH, Mamais A, Cookson MR (2019). LRRK2 links genetic and sporadic Parkinson’s disease. Biochem. Soc. Trans..

[14] Nguyen APT, Moore DJ (2017). Understanding the GTPase activity of LRRK2: regulation, function, and neurotoxicity. Adv. Neurobiol..

[15] Cresto N (2019). The unlikely partnership between LRRK2 and α‐synuclein in Parkinson’s disease. Eur. J. Neurosci..

[16] Majbour NK (2020). CSF total and oligomeric α-synuclein along with TNF-α as risk biomarkers for Parkinson’s disease: a study in LRRK2 mutation carriers. Transl. Neurodegener..

[17] Volpicelli-Daley LA (2016). G2019S-LRRK2 expression augments alpha-synuclein sequestration into inclusions in neurons. J. Neurosci..

[18] Bae E-J (2018). LRRK2 kinase regulates α-synuclein propagation via RAB35 phosphorylation. Nat. Commun..

[19] Azeggagh, S. & Berwick, D. C. The development of inhibitors of leucine‐rich repeat kinase 2 (LRRK2) as a therapeutic strategy for Parkinson’s disease: the current state of play. *Br. J. Pharmacol*. **179**, 1478–1495 (2022). 10.1111/bph.15575 34050929

[20] Baptista MAS (2020). LRRK2 inhibitors induce reversible changes in nonhuman primate lungs without measurable pulmonary deficits. Sci. Transl. Med..

[21] Liu H-F (2014). LRRK2 R1441G mice are more liable to dopamine depletion and locomotor inactivity. Ann. Clin. Transl. Neur..

[22] Reynolds A, Doggett EA, Riddle SM, Lebakken CS, Nichols RJ (2014). LRRK2 kinase activity and biology are not uniformly predicted by its autophosphorylation and cellular phosphorylation site status. Front. Mol. Neurosci..

[23] Prescott A (2011). Characterization of a selective inhibitor of the Parkinson’s disease kinase LRRK2. Nat. Chem. Biol..

[24] Sheng, Z. et al. Ser(1292) autophosphorylation is an indicator of LRRK2 kinase activity and contributes to the cellular effects of PD mutations. *Sci. Transl. Med*. **4**, 164ra161 (2012). 10.1126/scitranslmed.3004485 23241745

[25] Steger, M. et al. Phosphoproteomics reveals that Parkinson’s disease kinase LRRK2 regulates a subset of Rab GTPases. *eLife* **5**, e12813 (2016). 10.7554/eLife.12813 PMC4769169 26824392

[26] Flurkey, K., M. Currer, J. & Harrison, D. E. *Mouse Models in Aging Research*. 637–672 (Elsevier Inc, 2007).

[27] Kluss JH (2021). Preclinical modeling of chronic inhibition of the Parkinson’s disease associated kinase LRRK2 reveals altered function of the endolysosomal system in vivo. Mol. Neurodegener..

[28] Oueslati A (2016). Implication of alpha-synuclein phosphorylation at S129 in synucleinopathies: what have we learned in the last decade?. J. Parkinson Dis..

[29] Wallings RL, Herrick MK, Tansey MG (2020). LRRK2 at the interface between peripheral and central immune function in Parkinson’s. Front. Neurosci..

[30] Fell MJ (2015). MLi-2, a potent, selective, and centrally active compound for exploring the therapeutic potential and safety of LRRK2 kinase inhibition. J. Pharmacol. Exp. Ther..

[31] Weaver TE, Na C-L, Stahlman M (2002). Biogenesis of lamellar bodies, lysosome-related organelles involved in storage and secretion of pulmonary surfactant. Semin. Cell Dev. Biol..

[32] Lomask M (2006). Further exploration of the Penh parameter. Exp. Toxicol. Pathol..

[33] Abboud G, Kaplowitz N (2007). Drug-induced liver. Inj. Drug Saf..

[34] Meredith GE, Kang UJ (2006). Behavioral models of Parkinson’s disease in rodents: a new look at an old problem. Mov. Disord..

[35] Guenet JL (2005). The mouse genome. Genome Res..

[36] West AB (2005). Parkinson’s disease-associated mutations in leucine-rich repeat kinase 2 augment kinase activity. Proc. Natl Acad. Sci. USA.

[37] Langston RG, Rudenko IN, Cookson MR (2016). The function of orthologues of the human Parkinson’s disease gene LRRK2 across species: implications for disease modelling in preclinical research. Biochem. J..

[38] Fuji, R. N. et al. Effect of selective LRRK2 kinase inhibition on nonhuman primate lung. *Sci. Transl. Med*. **7**, 273ra15 (2015). 10.1126/scitranslmed.aaa3634 25653221

[39] Galter D (2006). LRRK2 expression linked to dopamine-innervated areas. Ann. Neurol..

[40] Westerlund M (2008). Developmental regulation of leucine-rich repeat kinase 1 and 2 expression in the brain and other rodent and human organs: Implications for Parkinson’s disease. Neurosci.

[41] Biskup S (2007). Dynamic and redundant regulation of LRRK2 and LRRK1 expression. BMC Neurosci..

[42] Liu H-F (2017). Combined LRRK2 mutation, aging and chronic low dose oral rotenone as a model of Parkinson’s disease. Sci. Rep. UK.

[43] Liu, H.-F. et al. Aberrant mitochondrial morphology and function associated with impaired mitophagy and DNM1L-MAPK/ERK signaling are found in aged mutant Parkinsonian LRRK2^R1441G^ mice. *Autophagy* **17**, 3196–3220 (2021). 10.1080/15548627.2020.1850008 PMC8526027 33300446

[44] Estrada AA (2012). Discovery of highly potent, selective, and brain-penetrable leucine-rich repeat kinase 2 (LRRK2) small molecule inhibitors. J. Med. Chem..

[45] Iannotta L (2020). Divergent effects of G2019S and R1441C LRRK2 mutations on LRRK2 and Rab10 phosphorylations in mouse tissues. Cells.

[46] Salazar C, Höfer T (2006). Kinetic models of phosphorylation cycles: a systematic approach using the rapid-equilibrium approximation for protein–protein interactions. Biosystems.

[47] Kasinathan N, Jagani HV, Alex AT, Volety SM, Rao JV (2015). Strategies for drug delivery to the central nervous system by systemic route. Drug Deliv..

[48] Pardridge WM (2005). The blood-brain barrier: bottleneck in brain drug development. NeuroRx.

[49] Shah NP (2008). Intermittent target inhibition with dasatinib 100 mg once daily preserves efficacy and improves tolerability in imatinib-resistant and -intolerant chronic-phase chronic myeloid leukemia. J. Clin. Oncol..

[50] Wong YC, Krainc D (2017). alpha-Synuclein toxicity in neurodegeneration: mechanism and therapeutic strategies. Nat. Med..

[51] Malfertheiner K, Stefanova N, Heras-Garvin A (2021). The concept of α-Synuclein strains and how different conformations may explain distinct neurodegenerative disorders. Front. Neurol..

[52] Ingelsson M (2016). alpha-Synuclein oligomers-neurotoxic molecules in parkinson’s disease and other Lewy Body disorders. Front. Neurosci..

[53] Danzer KM (2007). Different species of alpha-synuclein oligomers induce calcium influx and seeding. J. Neurosci..

[54] Ludtmann MHR (2018). α-synuclein oligomers interact with ATP synthase and open the permeability transition pore in Parkinson’s disease. Nat. Commun..

[55] Sharon R (2003). The formation of highly soluble oligomers of α-synuclein is regulated by fatty acids and enhanced in Parkinson’s disease. Neuron..

[56] Paleologou KE (2009). Detection of elevated levels of soluble α-synuclein oligomers in post-mortem brain extracts from patients with dementia with Lewy bodies. Brain.

[57] Parnetti L (2014). Cerebrospinal fluid lysosomal enzymes and alpha-synuclein in Parkinson’s disease. Mov. Disord..

[58] Erb ML, Moore DJ (2020). LRRK2 and the endolysosomal system in Parkinson’s disease. J. Parkinson’s Dis..

[59] Zhao Y (2020). LRRK2 kinase inhibitors reduce alpha-synuclein in human neuronal cell lines with the G2019S mutation. Neurobiol. Dis..

[60] Streubel-Gallasch L (2021). Parkinson’s disease–associated LRRK2 interferes with astrocyte-mediated alpha-synuclein clearance. Mol. Neurobiol..

[61] Zhao HT (2017). LRRK2 antisense oligonucleotides ameliorate α-synuclein inclusion formation in a Parkinson’s disease mouse model. Mol. Ther. Nucl. Acids.

[62] Madureira M, Connor-Robson N, Wade-Martins R (2020). “LRRK2: autophagy and lysosomal activity”. Front Neurosci..

[63] Hockey LN (2015). Dysregulation of lysosomal morphology by pathogenic LRRK2 is corrected by TPC2 inhibition. J. Cell. Sci..

[64] Schapansky J (2018). Familial knockin mutation of LRRK2 causes lysosomal dysfunction and accumulation of endogenous insoluble α-synuclein in neurons. Neurobiol. Dis..

[65] Matta S (2012). LRRK2 controls an EndoA phosphorylation cycle in synaptic endocytosis. Neuron.

[66] Islam MS (2016). Human R1441C LRRK2 regulates the synaptic vesicle proteome and phosphoproteome in a Drosophila model of Parkinson’s disease. Hum. Mol. Genet..

[67] Koss DJ, Campesan S, Giorgini F, Outeiro TF (2021). Dysfunction of RAB39B‐mediated vesicular trafficking in Lewy Body diseases. Mov. Disord..

[68] Goncalves SA (2016). shRNA-based screen identifies endocytic recycling pathway components that act as genetic modifiers of alpha-synuclein aggregation, secretion and toxicity. PLoS Genet..

[69] Chen RHC (2013). α-synuclein membrane association is regulated by the Rab3a recycling machinery and presynaptic activity. J. Biol. Chem..

[70] Lee H-J, Choi C, Lee S-J (2002). Membrane-bound α-synuclein has a high aggregation propensity and the ability to seed the aggregation of the cytosolic form. J. Biol. Chem..

[71] Boecker CA, Goldsmith J, Dou D, Cajka GG, Holzbaur ELF (2021). Increased LRRK2 kinase activity alters neuronal autophagy by disrupting the axonal transport of autophagosomes. Curr. Biol..

[72] Lee H-J, Khoshaghideh F, Patel S, Lee S-J (2004). Clearance of alpha-synuclein oligomeric intermediates via the lysosomal degradation pathway. J. Neurosci..

[73] Samuel F (2016). Effects of serine 129 phosphorylation on α-synuclein aggregation, membrane association, and internalization. J. Biol. Chem..

[74] Fujiwara H (2002). alpha-Synuclein is phosphorylated in synucleinopathy lesions. Nat. Cell Biol..

[75] Wang Y (2012). Phosphorylated alpha-synuclein in Parkinson’s disease. Sci. Transl. Med..

[76] de Lange EC (2013). The mastermind approach to CNS drug therapy: translational prediction of human brain distribution, target site kinetics, and therapeutic effects. Fluids Barriers CNS.

[77] Herzig MC (2011). LRRK2 protein levels are determined by kinase function and are crucial for kidney and lung homeostasis in mice. Hum. Mol. Genet.

[78] Paisán-Ruíz C (2004). Cloning of the gene containing mutations that cause PARK8-linked Parkinson’s disease. Neuron.

[79] Tian Y (2021). LRRK2 plays essential roles in maintaining lung homeostasis and preventing the development of pulmonary fibrosis. Proc. Natl Acad. Sci. USA.

[80] Singh F (2021). Pharmacological rescue of impaired mitophagy in Parkinson’s disease-related LRRK2 G2019S knock-in mice. eLife.

[81] Araki M, Ito K, Takatori S, Ito G, Tomita T (2021). BORCS6 is involved in the enlargement of lung lamellar bodies in Lrrk2 knockout mice. Hum. Mol. Genet.

[82] Miklavc P (2014). Surfactant secretion in LRRK2 knock-out rats: changes in lamellar body morphology and rate of exocytosis. PLoS ONE.

[83] Whiffin N (2020). The effect of LRRK2 loss-of-function variants in humans. Nat. Med..

[84] Gould S, Scott RC (2005). 2-Hydroxypropyl-beta-cyclodextrin (HP-beta-CD): a toxicology review. Food Chem. Toxicol..

[85] Donaubauer HH, Fuchs H, Langer KH, Bär A (1998). Subchronic intravenous toxicity studies with γ-cyclodextrin in rats. Regul. Toxicol. Pharmacol..

[86] Henics T, Wheatley DN (1999). Cytoplasmic vacuolation, adaptation and cell death: a view on new perspectives and features. Biol. Cell.

[87] Weindel, C. G. et al. LRRK2 maintains mitochondrial homeostasis and regulates innate immune responses to Mycobacterium tuberculosis. *Elife* **9**, e51071 (2020). 10.7554/eLife.51071 PMC7159881 32057291

[88] Tanaka T, Narazaki M, Kishimoto T (2014). IL-6 in inflammation, immunity, and disease. CSH Perspect. Biol..

[89] Rincon M (2012). Interleukin-6: from an inflammatory marker to a target for inflammatory diseases. Trends Immunol..

[90] Andres-Mateos E (2009). Unexpected lack of hypersensitivity in LRRK2 knock-out mice to MPTP (1-methyl-4-phenyl-1,2,3,6-tetrahydropyridine). J. Neurosci..

[91] Carelle-Calmels N (2009). Genetic compensation in a human genomic disorder. N. Engl. J. Med..

[92] Junying YU (2007). Induced pluripotent stem cell lines derived from human somatic cells. Science.

[93] Orenstein JM, Graham L (2007). Processing tissue and cells for transmission electron microscopy in diagnostic pathology and research. Nat. Protoc..

[94] Grubbs FE (1969). Procedures for detecting outlying observations in samples. Technometrics.

