# Supplementary material for: Long-term inhibition of mutant LRRK2 hyper-kinase activity reduced mouse brain α-synuclein oligomers without adverse effects
Source: NPJ Parkinsons Dis. 2022 Sep 10;8:115. doi: 10.1038/s41531-022-00386-9 (PMC9464237; doi:10.1038/s41531-022-00386-9)

Supplementary information

Supplementary Figures

Supplementary Figure 1

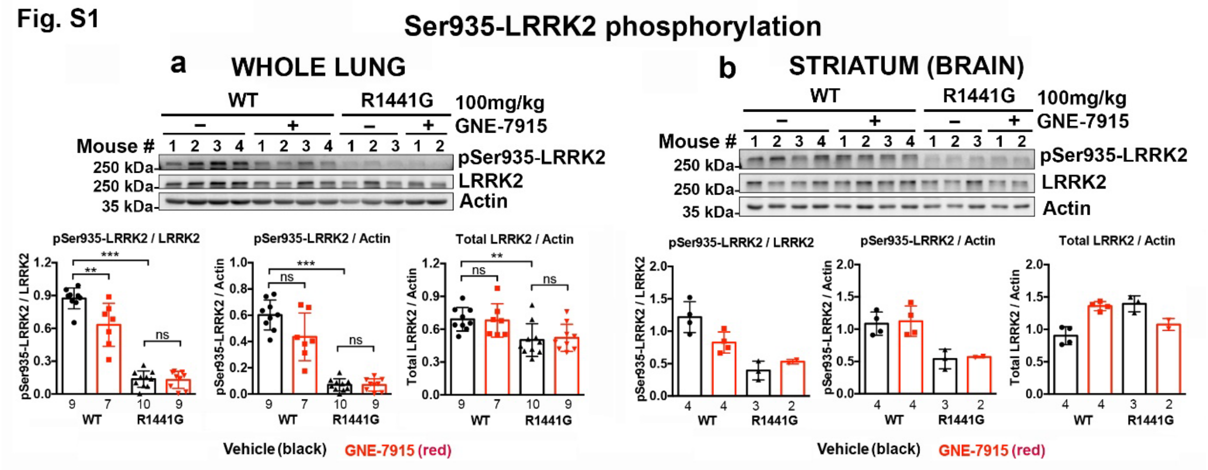

Supplementary Figure 1: Phosphorylated Ser935-LRRK2 level in LRRK2^{R1441G} mutant mice is not applicable for assessment of LRRK2 kinase inhibition by GNE-7915. The basal level of pSer935-LRRK2 in LRRK2^{R1441G} mutant **(a)** lung and, **(b)** brain striatum were significantly lower than those of age-matched WT mice. The inhibitory effect of GNE-7915 on reducing pSer935-LRRK2 in lung and brain striatum was not shown significantly in LRRK2^{R1441G} mutant mice because of very low basal level of pSer935-LRRK2. Data are expressed as mean ± SEM. ***p<0.001 & **p<0.01 represent statistical significance between designated groups by non-parametric Mann-Whitney test. ns: not significant.

Supplementary Figure 2

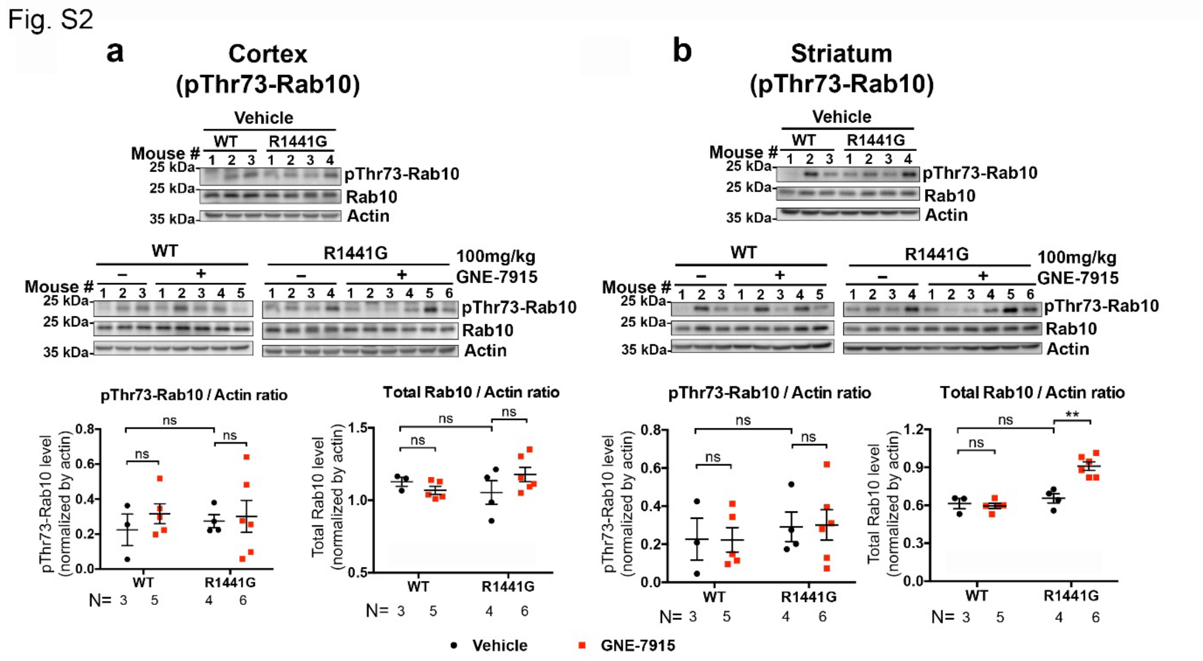

Supplementary Figure 2: Phosphorylated Thr73-Rab10 levels were highly variable among individual mouse brain and did not correlate with LRRK2 inhibition by GNE-7915. **(a)** Whole cortex and **(b)** striatum of the brain were freshly harvested after 18-week GNE-7915 treatment for western blotting of pThr73-Rab10. Data were expressed as mean ± SEM. X-axis numbers represent total number of animals in each treatment group. **p<0.01 represents statistical significance between groups by Mann-Whitney (unpaired, nonparametric) test. ns: not significant.

Supplementary Figure 3

Fig. S3

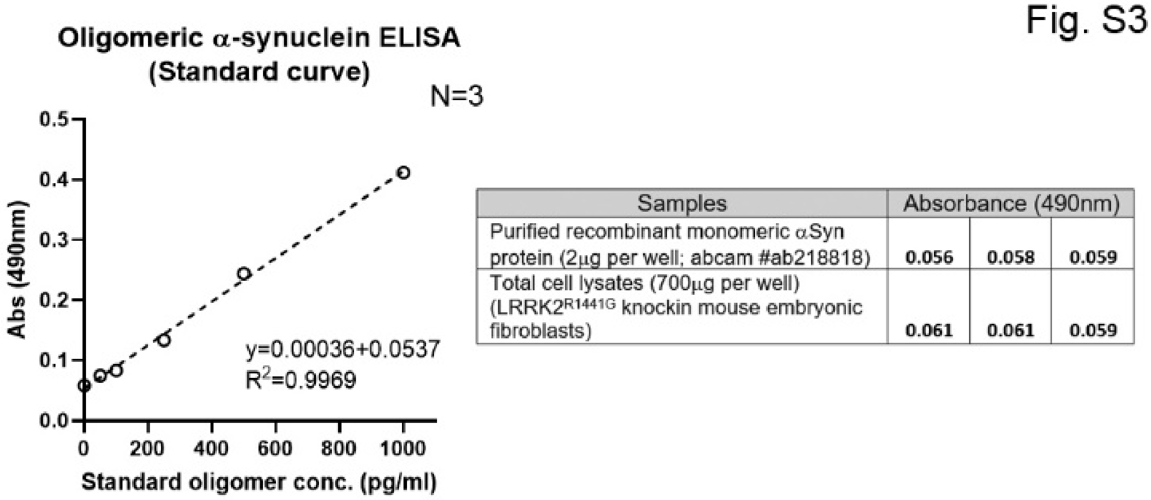

Samples	Absorbance (490nm)		
Purified recombinant monomeric αSyn protein (2μg per well; abcam #ab218818)	0.056	0.058	0.059
Total cell lysates (700μg per well) (LRRK2^{R1441G} knockin mouse embryonic fibroblasts)	0.061	0.061	0.059

Supplementary Figure 3: Specificity of oligomeric a-synuclein ELISA. Validation of assay specificity confirmed that this commercial ELISA does not cross-react with two independent negative controls, 1) total cell lysates extracted from mouse embryonic fibroblasts (MEFs; which does not express αSyn), and 2) a purified recombinant monomeric αSyn protein (Abcam™; #ab218818).

Supplementary Figure 4

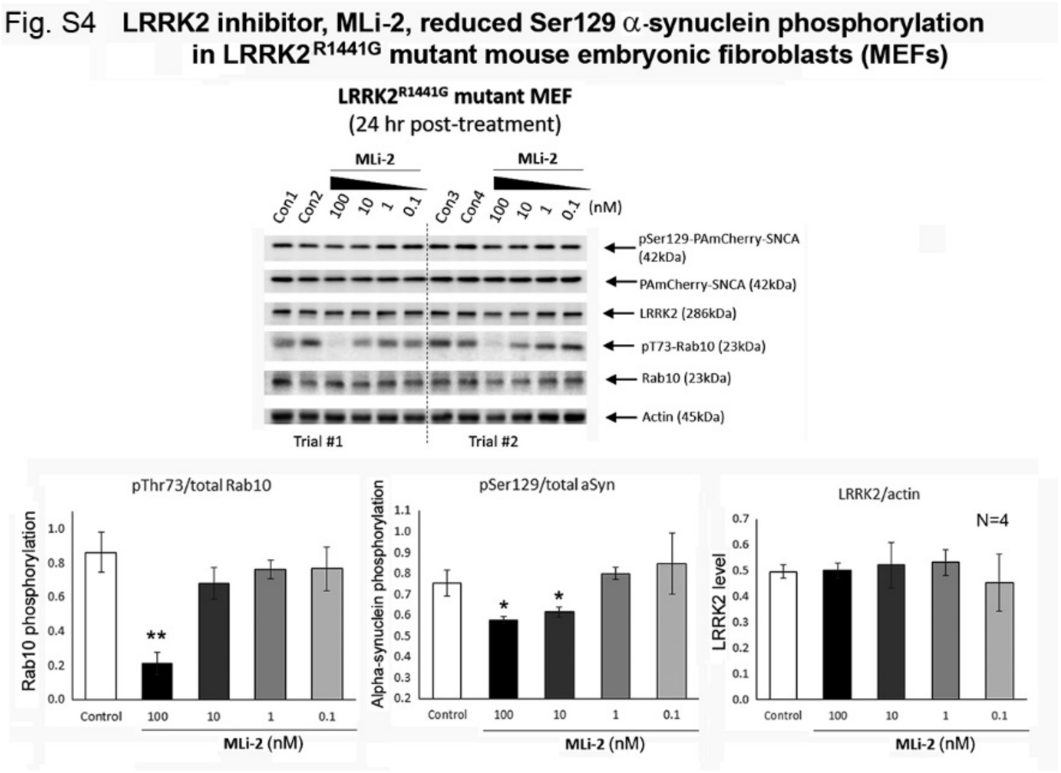

Supplementary Figure 4: Treatment of LRRK2 inhibitor, MLi-2, reduced Ser129-αSyn phosphorylation. MLi-2 was freshly dissolved in DMSO at stock concentration of 100mM. LRRK2^{R1441G} mutant MEFs engineered to express high level of recombinant alpha-synuclein (i.e. PAmCherry-SNCA) were treated with graded doses of MLi-2 (100, 10, 10, 1 and 0.1nM) for 24hr before harvested for Western blot analyses. MLi-2 at 100nM significantly reduced phosphorylation of Thr73 Rab10, indicating LRRK2 kinase inhibition. MLi-2 at 10 and 100nM significantly reduced Ser129-αSyn phosphorylation compared to untreated controls. Data were expressed as mean ± S.E.M. of four independent treatments (N=4). *$p<0.05$ & **$p<0.01$ represent statistical significance, compared to untreated controls, using unpaired Student's t test.

Supplementary Figure 5

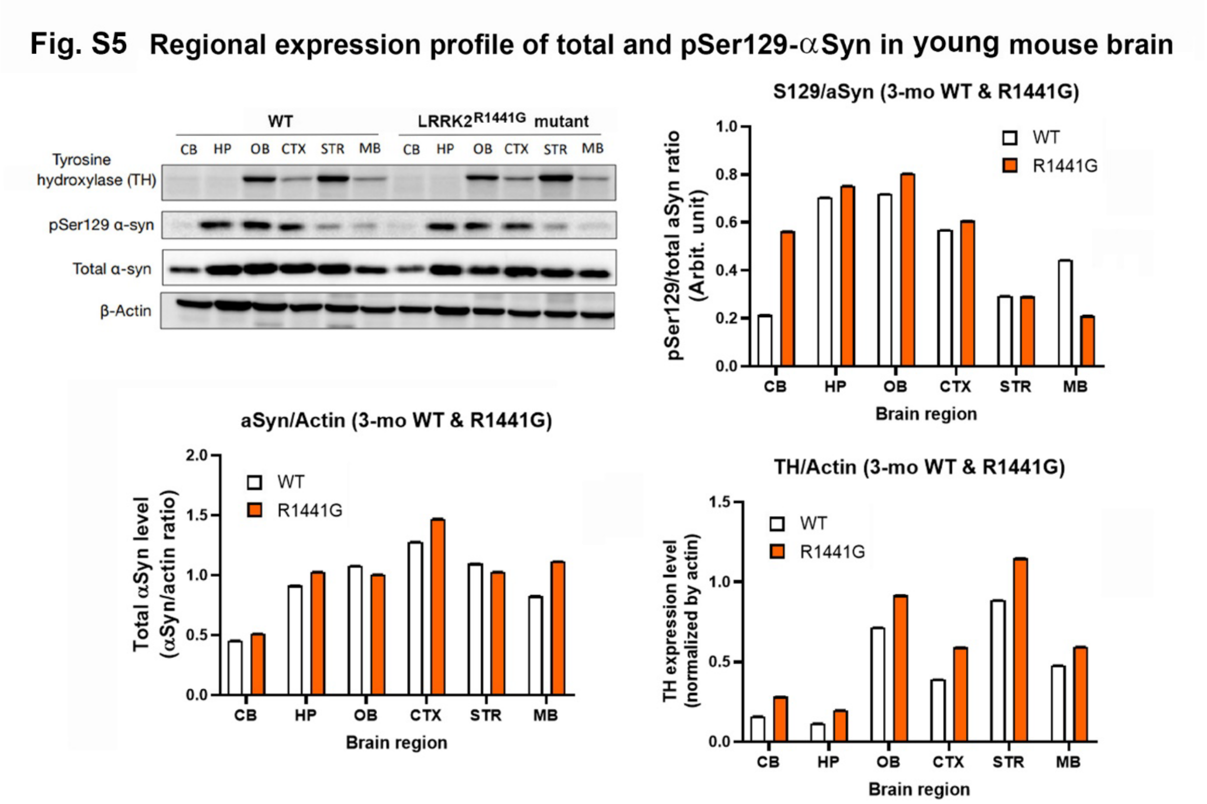

Supplementary Figure 5. Relative expression level of phosphorylated αSyn-Ser129 (pSer129) in different brain regions of young (3-month old) WT and LRRK2^{R1441G} mutant mice. Different brain regions were freshly dissected and lysed in cooled standard RIPA buffer. Total lysates were electrophoresed in SDS-PAGE and subjected to Western blotting against tyrosine hydroxylase (TH; marker protein of dopaminergic cells), pSer129 and total αSyn. The relative levels of pSer129 were determined in dopaminoceptive brain regions. To determine dopaminoceptive regions in mouse brain, we assessed tyrosine hydroxylase [TH; marker protein of dopaminergic (DA) neurons] expression in olfactory bulb (OB), cortex (CTX), hippocampus (HP), striatum (STR), and midbrain (MB) indicating regions enriched with DA neurons. The expression level of total αSyn is highest in STR, followed by CTX, OB, HP, MB, Spinal cord (SC) and CB. Western blot of TH showed that OB and STR were highly enriched with DA cells whereas CTX and MB have relatively fewer. TH was not detected in CB and HP. OB, HP, CTX expressed relatively higher levels of pSer129, and STR and MB expressed at relatively a lower level. Except CB which expressed relatively lower level of αSyn, the levels of total αSyn were similar in OB, HP, CTX, STR and MB of young WT and LRRK2 mutant mouse brains. Although αSyn was also detected in CB, pSer129 αSyn level was barely detectable in this region.

Supplementary Figure 6

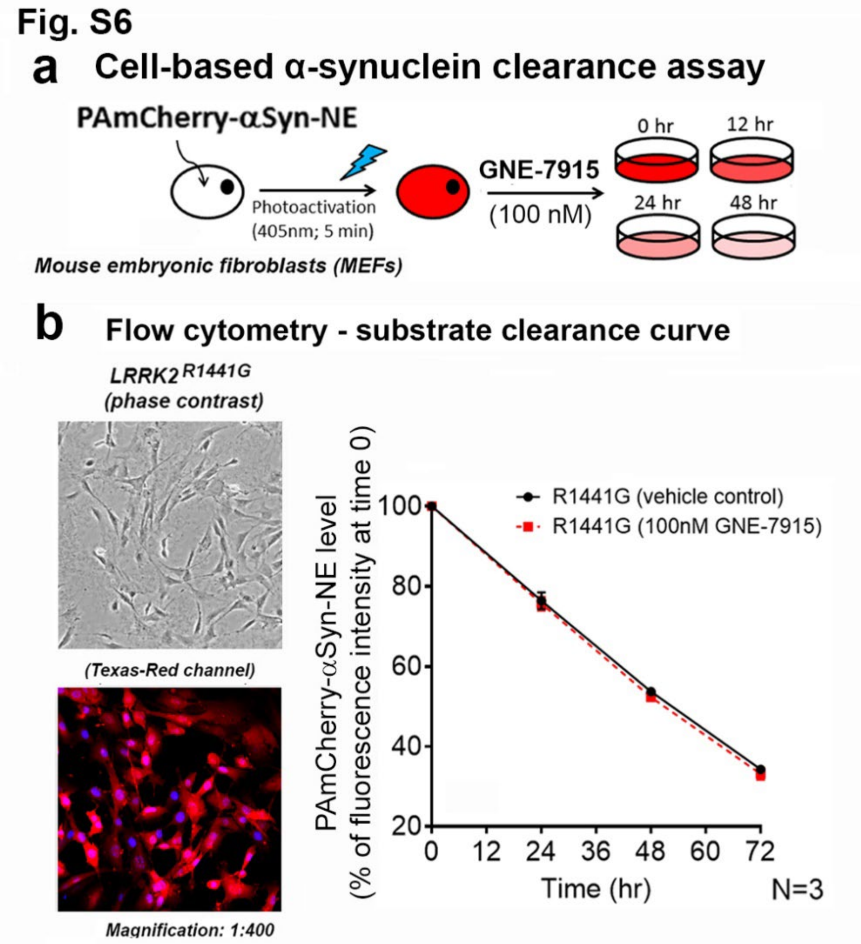

Supplementary Figure 6. GNE-7915 treatment did not alter cellular clearance of αSyn. **(a)** Immortalized LRRK2^{R1441G} mutant mouse embryonic fibroblasts (MEFs) stably expressing full-length mouse αSyn conjugated with photoactivatable PAmCherry protein (Ho *et al*., Autophagy 2020) were treated with GNE-7915 (100nM) for 72hr. **(b)** Representative flow cytometry intensity plots of photoactivated mutant MEFs harvested at different time points for αSyn clearance. Rate of protein clearance was expressed as time-dependent change in levels of PAmCherry fluorescence in mutant MEFs compared with the level at time t = 0. GNE-7915 treatment for 72 hr did not significantly affect αSyn clearance (N=3). Data are expressed as mean ± S.E.M.

Supplementary Table S1:

Two-way ANOVA of <u>CORTEX</u> α-synuclein oligomer levels in WT and LRRK2^{R1441G} mutant mice after 18-week GNE-7915 administration (Fig. 3)

**Two-way ANOVA**	Ordinary				
Alpha	0.05				
Source of Variation	% of total variation	P value	P value summary	Significant?	
**Interaction**	**0.2502**	**0.6289**	**ns**	**No**	
**LRRK2 mutation**	**22.48**	**<0.0001**	********	**Yes**	
**GNE7915 effect**	**2.730**	**0.1133**	**ns**	**No**	
ANOVA table	SS (Type III)	DF	MS	F (DFn, DFd)	P value
Interaction	0.006707	1	0.006707	F (1, 71) = 0.2356	P=0.6289
LRRK2 mutation	0.6027	1	0.6027	F (1, 71) = 21.18	P<0.0001
GNE7915 effect	0.07319	1	0.07319	F (1, 71) = 2.571	P=0.1133
Residual	2.021	71	0.02846		

Tukey's multiple comparisons test	Predicted (LS) mean diff.	95.00% CI of diff.	Summary	Adjusted P Value
WT:Veh vs. WT:GNE7915	0.08159	-0.06262 to 0.2258	ns	0.4497
WT:Veh vs. R1441G:Veh	-0.1608	-0.3072 to -0.01435	*	0.0258
WT:Veh vs. R1441G:GNE7915	-0.1171	-0.2575 to 0.02326	ns	0.1346
WT:GNE7915 vs. R1441G:Veh	-0.2424	-0.3925 to -0.09225	***	0.0004
WT:GNE7915 vs. R1441G:GNE7915	-0.1987	-0.3429 to -0.05448	**	0.0030
R1441G:Veh vs. R1441G:GNE7915	0.04367	-0.1028 to 0.1901	ns	0.8612

Single factor analysis:

D'Agostino & Pearson test	WT-Veh	WT-GNE	R1441G-Veh	R1441G-GNE
K2	1.812	1.303	1.153	0.7277
P value	0.4042	0.5213	0.5618	0.6950
Passed normality test (alpha=0.05)?	Yes	Yes	Yes	Yes
P value summary	ns	ns	ns	ns

Method to identify outliers				
**Grubbs (Alpha = 0.05)**	G = 2.375	G = 1.715	G = 2.073	G = 2.018
Number of points				
# Y values analyzed	20	18	16	20
Outliers	0	0	0	0

Unpaired, parametric Students' t test	WT-Veh vs. R1441G-Veh	R1441G-Veh vs. R1441G-GNE
P value	**0.0015**	**0.1851**
P value summary	**	**ns**
Significantly different (P < 0.05)?	Yes	No
One- or two-tailed P value?	Two-tailed	Two-tailed
t, df	t=3.442, df=34	t=1.352, df=34
**F test to compare variances**		
F, DFn, Dfd	3.917, 19, 15	3.264, 19, 15
P value	0.0099	0.0241
P value summary	**	*
Significantly different (P < 0.05)?	Yes	Yes
**Mann Whitney U-test (unpaired, non-parametric)**		

P value	**0.0019**	**0.2653**
Exact or approximate P value?	**Exact**	**Exact**
P value summary	**	**ns**
Significantly different (P < 0.05)?	Yes	No
One- or two-tailed P value?	Two-tailed	Two-tailed
Sum of ranks in column A,C	275 , 391	331.5 , 334.5
Mann-Whitney U	65	124.5

Two-way ANOVA of STRIATUM α-synuclein oligomer levels in WT and LRRK2^{R1441G} mutant mice after 18-week GNE-7915 administration (Fig. 3)

**Two-way ANOVA**	Ordinary				
Alpha	0.05				
Source of Variation	% of total variation	P value	P value summary	Significant?	
**Interaction**	**8.128**	**0.0066**	**	**Yes**	
**LRRK2 mutation**	**12.11**	**0.0011**	**	**Yes**	
**GNE7915 treatment**	**5.900**	**0.0198**	*	**Yes**	
ANOVA table	SS (Type III)	DF	MS	F (DFn, DFd)	P value
Interaction	3.621	1	3.621	F (1, 72) = 7.823	P=0.0066
LRRK2 mutation	5.396	1	5.396	F (1, 72) = 11.66	P=0.0011
GNE7915 treatment	2.628	1	2.628	F (1, 72) = 5.678	P=0.0198
Residual	33.32	72	0.4628		

**Tukey's multiple comparisons test**	Predicted (LS) mean diff.	95.00% CI of diff.	Summary	Adjusted P Value
WT:Veh vs. WT:GNE7915	-0.06467	-0.6452 to 0.5159	ns	0.9912
WT:Veh vs. R1441G:Veh	-0.9701	-1.559 to -0.3816	***	0.0003
WT:Veh vs. R1441G:GNE7915	-0.1611	-0.7344 to 0.4121	ns	0.8809
WT:GNE7915 vs. R1441G:Veh	-0.9055	-1.494 to -0.3169	***	0.0007
WT:GNE7915 vs. R1441G:GNE7915	-0.09646	-0.6697 to 0.4768	ns	0.9708
R1441G:Veh vs. R1441G:GNE7915	0.8090	0.2277 to 1.390	**	0.0026

Single factor analysis:

**D'Agostino & Pearson test**	**WT-Veh**	**WT-GNE**	**R1441G-Veh**	**R1441G-GNE**
K2	2.718	1.214	0.9874	1.714
P value	0.2569	0.5449	0.6104	0.4244
Passed normality test (alpha=0.05)?	Yes	Yes	Yes	Yes
P value summary	ns	ns	ns	ns

Method to identify outliers				
**Grubbs (Alpha = 0.05)**	G = 2.512	G = 1.985	G = 1.663	G = 1.865
Number of points				
# Y values analyzed	19	19	15	20
Outliers	0	0	0	0

**Unpaired, parametric Students' t test**	**WT-Veh vs. R1441G-Veh**	**R1441G-Veh vs. R1441G-GNE**
P value	**0.0016**	**0.0132**
P value summary	**	*
Significantly different (P < 0.05)?	Yes	Yes
One- or two-tailed P value?	Two-tailed	Two-tailed
t, df	t=3.441, df=32	t=2.619, df=33
**F test to compare variances**		

F, DFn, Dfd	1.776, 18, 14	1.952, 19, 14
P value	0.2799	0.2062
P value summary	ns	ns
Significantly different (P < 0.05)?	No	No
**Mann Whitney U-test (unpaired, non-parametric)**		
P value	**0.0006**	**0.0169**
Exact or approximate P value?	**Exact**	**Exact**
P value summary	***	*
Significantly different (P < 0.05)?	Yes	Yes
One- or two-tailed P value?	Two-tailed	Two-tailed
Sum of ranks in column A,C	237.5 , 357.5	341 , 289
Mann-Whitney U	47.50	79

Two-way ANOVA of <u>CORTEX</u> pSer129-αSyn levels in WT and LRRK2^{R1441G} mutant mice after 18-weeks GNE-7915 administration (Fig. 4)

**Two-way ANOVA**	Ordinary				
Alpha	0.05				
Source of Variation	% of total variation	P value	P value summary	Significant?	
**Interaction**	**11.89**	**0.0022**	**	**Yes**	
**Mutation**	**2.998**	**0.1144**	**ns**	**No**	
**GNE-7915**	**0.2034**	**0.6785**	**ns**	**No**	
ANOVA table	SS (Type III)	DF	MS	F (DFn, DFd)	P value
Interaction	0.1292	1	0.1292	F (1, 72) = 10.13	P=0.0022
WT / R1441G	0.03257	1	0.03257	F (1, 72) = 2.553	P=0.1144
Vehicle / GNE-7915	0.002210	1	0.002210	F (1, 72) = 0.1732	P=0.6785
Residual	0.9185	72	0.01276		

**Two-way ANOVA Analysis**<br>**Tukey's multiple comparisons test**<br><br>**pSer129-αSyn / Total αSyn**<br>**(level of phosphorylation)**	**Significant?**	**Summary**	**Adjusted P Value**
**WT:Vehicle vs. WT:GNE-7915**	No	ns	0.2138
**WT:Vehicle vs. R1441G:Vehicle**	No	ns	0.6869
WT:Vehicle vs. R1441G:GNE-7915	No	ns	0.4769
WT:GNE-7915 vs. R1441G:Vehicle	No	ns	0.8425
WT:GNE-7915 vs. R1441G:GNE-7915	Yes	**	0.0055
**R1441G:Vehicle vs. R1441G:GNE-7915**	No	ns	0.0618
**Two-way ANOVA Analysis**<br>**Tukey's multiple comparisons test**<br><br>**pSer129-αSyn / Actin**<br>**(cellular amount of phospho-αSyn)**	**Significant?**	**Summary**	**Adjusted P Value**
**WT:Vehicle vs. WT:GNE-7915**	No	ns	0.1389
**WT:Vehicle vs. R1441G:Vehicle**	No	ns	0.1818
WT:Vehicle vs. R1441G:GNE-7915	No	ns	0.8482
WT:GNE-7915 vs. R1441G:Vehicle	No	ns	0.9996
WT:GNE-7915 vs. R1441G:GNE-7915	Yes	*	0.0180
**R1441G:Vehicle vs. R1441G:GNE-7915**	Yes	*	0.0270

**Two-way ANOVA Analysis**<br>**Tukey's multiple comparisons test**<br><br>**Total αSyn / Actin**	**Significant?**	**Summary**	**Adjusted P Value**
**WT:Vehicle vs. WT:GNE-7915**	No	ns	0.3700
**WT:Vehicle vs. R1441G:Vehicle**	No	ns	0.2098
WT:Vehicle vs. R1441G:GNE-7915	No	ns	0.9194
WT:GNE-7915 vs. R1441G:Vehicle	No	ns	0.9836
WT:GNE-7915 vs. R1441G:GNE-7915	No	ns	0.7440
**R1441G:Vehicle vs. R1441G:GNE-7915**	No	ns	0.5264

Single factor analysis:

**D'Agostino & Pearson test**<br><br>**pSer129-αSyn / Total αSyn**	**WT-Veh**	**WT-GNE**	**R1441G-Veh**	**R1441G-GNE**
K2	0.3542	0.2356	1.523	0.9795
P value	0.8377	0.8889	0.4669	0.6128
Passed normality test (alpha=0.05)?	Yes	Yes	Yes	Yes
P value summary	ns	ns	ns	ns

**D'Agostino & Pearson test**<br><br>**pSer129-αSyn / Actin**	**WT-Veh**	**WT-GNE**	**R1441G-Veh**	**R1441G-GNE**
K2	1.688	1.990	2.907	1.688
P value	0.4301	0.3697	0.2337	0.4301
Passed normality test (alpha=0.05)?	Yes	Yes	Yes	Yes
P value summary	ns	ns	ns	ns

**Unpaired, parametric Student's t-test**<br><br>**pSer129-αSyn / Total αSyn**	**R1441G-Veh vs. R1441G-GNE**	**WT-Veh vs. R1441G-Veh**	**WT-Veh vs. WT-GNE**
P value	**0.0077**	**0.1666**	**0.0682**
P value summary	**	**ns**	**ns**
Significantly different? (P < 0.05)	Yes	No	No
One- or two-tailed P value?	Two-tailed	Two-tailed	Two-tailed
t, df	t=2.824 df=36	t=1.413 df=35	t=1.880 df=36
**F test to compare variances**			
F,DFn, Dfd	1.565, 19, 17	1.182, 17, 18	2.850, 18, 18
P value	0.3581	0.7267	0.0320
P value summary	ns	ns	*
Significantly different? (P < 0.05)	No	No	Yes

**Mann-Whitney U-test (unpaired, non-parametric)**<br><br>**pSer129-αSyn / Total αSyn**	**R1441G-Veh vs. R1441G-GNE**	**WT-Veh vs. R1441G-Veh**	**WT-Veh vs. WT-GNE**
P value	**0.0146**	**0.2959**	**0.1082**
Exact or approximate P value?	**Exact**	**Exact**	**Exact**

P value summary	*	**ns**	**ns**
Significantly different? (P < 0.05)	Yes	No	No
One- or two-tailed P value?	Two-tailed	Two-tailed	Two-tailed
Sum of ranks in column C,D	434.0 , 307.0	326.0 , 377.0	315.0 , 426.0
Mann-Whitney U	97.00	136.0	125.0

**Unpaired, parametric Student's t-test**<br><br>**pSer129-αSyn / Actin**	**R1441G-Veh vs. R1441G-GNE**	**WT-Veh vs. R1441G-Veh**	**WT-Veh vs. WT-GNE**
P value	**0.0068**	**0.0229**	**0.0359**
P value summary	**	*	*
Significantly different? (P < 0.05)	Yes	Yes	Yes
One- or two-tailed P value?	Two-tailed	Two-tailed	Two-tailed
t, df	t=2.869 df=36	t=2.380 df=35	t=2.180 df=36
**F test to compare variances**			
F,DFn, Dfd	1.441, 19, 17	1.218, 17, 18	1.996, 18, 18
P value	0.4534	0.6812	0.1520
P value summary	ns	ns	ns
Significantly different? (P < 0.05)	No	No	No

**Mann-Whitney U-test (unpaired, non-parametric)**<br><br>**pSer129-αSyn / Actin**	**R1441G-Veh vs. R1441G-GNE**	**WT-Veh vs. R1441G-Veh**	**WT-Veh vs. WT-GNE**
P value	**0.0064**	**0.0421**	**0.1706**
Exact or approximate P value?	**Exact**	**Exact**	**Exact**
P value summary	**	*	**ns**
Significantly different? (P < 0.05)	Yes	Yes	No
One- or two-tailed P value?	Two-tailed	Two-tailed	Two-tailed
Sum of ranks in column C,D	443.0 , 298.0	294.0 , 409.0	323.0 , 418.0
Mann-Whitney U	88.00	104.0	133.0

Two-way ANOVA of STRIATUM pSer129-αSyn levels in WT and LRRK2[R1441G] mutant mice after 18-weeks GNE-7915 administration (Fig. 4)

**Two-way ANOVA**	Ordinary				
Alpha	0.05				
Source of Variation	% of total variation	P value	P value summary	Significant?	
**Interaction**	**0.05619**	**0.8337**	**ns**	**No**	
**Mutation**	**1.120**	**0.3500**	**ns**	**No**	
**GNE-7915**	**13.75**	**0.0016**	**	**Yes**	
ANOVA table	SS (Type III)	DF	MS	F (DFn, DFd)	P value
Interaction	0.0008778	1	0.0008778	F (1, 67) = 0.04445	P=0.8337
WT / R1441G	0.01750	1	0.01750	F (1, 67) = 0.8860	P=0.3500
Vehicle / GNE-7915	0.2148	1	0.2148	F (1, 67) = 10.88	P=0.0016
Residual	1.323	67	0.01975		

Two-way ANOVA Analysis<br>Tukey's multiple comparisons test<br><br>pSer129-αSyn / Total αSyn<br>(level of phosphorylation)	Significant?	Summary	Adjusted P Value
**WT:Vehicle vs. WT:GNE-7915**	No	ns	0.0685
**WT:Vehicle vs. R1441G:Vehicle**	No	ns	0.8553
WT:Vehicle vs. R1441G:GNE-7915	Yes	*	0.0162
WT:GNE-7915 vs. R1441G:Vehicle	No	ns	0.3684
WT:GNE-7915 vs. R1441G:GNE-7915	No	ns	0.9520
**R1441G:Vehicle vs. R1441G:GNE-7915**	No	ns	0.1441
**Two-way ANOVA Analysis<br>Tukey's multiple comparisons test<br><br>pSer129-αSyn / Actin<br>(cellular amount of phospho-αSyn)**	**Significant?**	**Summary**	**Adjusted P Value**
**WT:Vehicle vs. WT:GNE-7915**	No	ns	0.7918
**WT:Vehicle vs. R1441G:Vehicle**	No	ns	0.9068
WT:Vehicle vs. R1441G:GNE-7915	No	ns	0.9921
WT:GNE-7915 vs. R1441G:Vehicle	No	ns	0.9960
WT:GNE-7915 vs. R1441G:GNE-7915	No	ns	0.9133
**R1441G:Vehicle vs. R1441G:GNE-7915**	No	ns	0.9767
**Two-way ANOVA Analysis<br>Tukey's multiple comparisons test<br><br>Total αSyn / Actin**	**Significant?**	**Summary**	**Adjusted P Value**
**WT:Vehicle vs. WT:GNE-7915**	No	ns	0.1344
**WT:Vehicle vs. R1441G:Vehicle**	No	ns	0.3003
WT:Vehicle vs. R1441G:GNE-7915	Yes	***	0.0004
WT:GNE-7915 vs. R1441G:Vehicle	No	ns	0.9827
WT:GNE-7915 vs. R1441G:GNE-7915	No	ns	0.2055
**R1441G:Vehicle vs. R1441G:GNE-7915**	No	ns	0.1097

Single factor analysis:

D'Agostino & Pearson test<br><br>pSer129-αSyn / Total αSyn	WT-Veh	WT-GNE	R1441G-Veh	R1441G-GNE
K2	0.8221	1.222	0.3871	1.387
P value	0.6629	0.5429	0.8240	0.4997
Passed normality test (alpha=0.05)?	Yes	Yes	Yes	Yes
P value summary	ns	ns	ns	ns

D'Agostino & Pearson test<br><br>pSer129-αSyn / Actin	WT-Veh	WT-GNE	R1441G-Veh	R1441G-GNE
K2	0.8717	0.04503	0.4955	0.2202
P value	0.6467	0.9777	0.7806	0.8957
Passed normality test (alpha=0.05)?	Yes	Yes	Yes	Yes
P value summary	ns	ns	ns	ns

**Unpaired, parametric Student's t-test**<br><br>**pSer129-αSyn / Total αSyn**	**R1441G-Veh vs. R1441G-GNE**	**WT-Veh vs. R1441G-Veh**	**WT-Veh vs. WT-GNE**
P value	**0.0834**	**0.3884**	**0.0018**
P value summary	**ns**	**ns**	**
Significantly different? (P < 0.05)	No	No	Yes
One- or two-tailed P value?	Two-tailed	Two-tailed	Two-tailed
t, df	t=1.785 df=33	t=0.8744 df=32	t=3.391 df=34
**F test to compare variances**			
F,DFn, Dfd	1.034, 15, 18	6.083, 15, 17	3.432, 17, 17
P value	0.9346	0.0006	0.0150
P value summary	ns	***	*
Significantly different? (P < 0.05)	No	Yes	Yes

**Mann-Whitney U-test (unpaired, non-parametric)**<br><br>**pSer129-αSyn / Total αSyn**	**R1441G-Veh vs. R1441G-GNE**	**WT-Veh vs. R1441G-Veh**	**WT-Veh vs. WT-GNE**
P value	**0.0813**	**0.3994**	**0.0016**
Exact or approximate P value?	**Exact**	**Exact**	**Exact**
P value summary	**ns**	**ns**	**
Significantly different? (P < 0.05)	No	No	Yes
One- or two-tailed P value?	Two-tailed	Two-tailed	Two-tailed
Sum of ranks in column C,D	235.0 , 395.0	290.0 , 305.0	236.0 , 430.0
Mann-Whitney U	99.00	119.0	65.00

**Unpaired, parametric Student's t-test**<br><br>**pSer129-αSyn / Actin**	**R1441G-Veh vs. R1441G-GNE**	**WT-Veh vs. R1441G-Veh**	**WT-Veh vs. WT-GNE**
P value	**0.7227**	**0.4551**	**0.2781**
P value summary	**ns**	**ns**	**ns**
Significantly different? (P < 0.05)	No	No	No
One- or two-tailed P value?	Two-tailed	Two-tailed	Two-tailed
t, df	t=0.3579 df=33	t=0.7561 df=32	t=1.102 df=34
**F test to compare variances**			
F,DFn, Dfd	1.246, 18, 15	2.409, 15, 17	1.946, 17, 17
P value	0.6732	0.0841	0.1802
P value summary	ns	ns	ns
Significantly different? (P < 0.05)	No	No	No

**Mann-Whitney U-test (unpaired, non-parametric)**<br><br>**pSer129-αSyn / Actin**	**R1441G-Veh vs. R1441G-GNE**	**WT-Veh vs. R1441G-Veh**	**WT-Veh vs. WT-GNE**
P value	**0.6301**	**0.3994**	**0.3517**
Exact or approximate P value?	**Exact**	**Exact**	**Exact**

P value summary	ns	ns	ns
Significantly different? (P < 0.05)	No	No	No
One- or two-tailed P value?	Two-tailed	Two-tailed	Two-tailed
Sum of ranks in column C,D	303.0 , 327.0	290.0 , 305.0	303.0 , 363.0
Mann-Whitney U	137.0	119.0	132.0

Two-way ANOVA of <u>CORTEX</u> pRab12 levels in WT and LRRK2^{R1441G} mutant mice after 18-weeks GNE-7915 administration (Fig. 2)

Table Analyzed	pRab12/total (figure)-updated				
**Two-way ANOVA**	Ordinary				
Alpha	0.05				
Source of Variation	% of total variation	P value	P value summary	Significant?	
**Interaction**	**11.72**	**0.0032**	**	**Yes**	
**Mutation**	**0.1090**	**0.7689**	**ns**	**No**	
**GNE-7915**	**4.368**	**0.0663**	**ns**	**No**	
ANOVA table	SS (Type III)	DF	MS	F (DFn, DFd)	P value
Interaction	0.1409	1	0.1409	F (1, 67) = 9.351	P=0.0032
WT / R1441G	0.001311	1	0.001311	F (1, 67) = 0.08701	P=0.7689
Vehicle / GNE-7915	0.05253	1	0.05253	F (1, 67) = 3.486	P=0.0663
Residual	1.010	67	0.01507		

**Two-way ANOVA Analysis<br>Tukey's multiple comparisons test<br><br>pRab12 / Total Rab12<br>(level of phosphorylation)**	**Significant?**	**Summary**	**Adjusted P Value**
**WT:Vehicle vs. WT:GNE-7915**	No	ns	0.8312
**WT:Vehicle vs. R1441G:Vehicle**	No	ns	0.2216
WT:Vehicle vs. R1441G:GNE-7915	No	ns	0.4198
WT:GNE-7915 vs. R1441G:Vehicle	No	ns	0.6885
WT:GNE-7915 vs. R1441G:GNE-7915	No	ns	0.0892
**R1441G:Vehicle vs. R1441G:GNE-7915**	Yes	**	0.0051
**Two-way ANOVA Analysis<br>Tukey's multiple comparisons test<br><br>pRab12 / Actin<br>(cellular amount of phospho-Rab12)**	**Significant?**	**Summary**	**Adjusted P Value**
**WT:Vehicle vs. WT:GNE-7915**	No	ns	0.6590
**WT:Vehicle vs. R1441G:Vehicle**	Yes	*	0.0268
WT:Vehicle vs. R1441G:GNE-7915	No	ns	0.9899
WT:GNE-7915 vs. R1441G:Vehicle	No	ns	0.3184
WT:GNE-7915 vs. R1441G:GNE-7915	No	ns	0.4678
**R1441G:Vehicle vs. R1441G:GNE-7915**	Yes	*	0.0115
**Two-way ANOVA Analysis<br>Tukey's multiple comparisons test<br><br>Total Rab12 / Actin**	**Significant?**	**Summary**	**Adjusted P Value**
**WT:Vehicle vs. WT:GNE-7915**	No	ns	0.9969
**WT:Vehicle vs. R1441G:Vehicle**	No	ns	0.4197
WT:Vehicle vs. R1441G:GNE-7915	No	ns	0.8112
WT:GNE-7915 vs. R1441G:Vehicle	No	ns	0.5431
WT:GNE-7915 vs. R1441G:GNE-7915	No	ns	0.9038

**R1441G:Vehicle vs. R1441G:GNE-7915**	No	ns	0.9098

Single factor analysis:

**D'Agostino & Pearson test**<br><br>**pRab12 / Total Rab12**	**WT-Veh**	**WT-GNE**	**R1441G-Veh**	**R1441G-GNE**
K2	3.207	2.021	0.1677	0.6850
P value	0.2012	0.3641	0.9196	0.7100
Passed normality test (alpha=0.05)?	Yes	Yes	Yes	Yes
P value summary	ns	ns	ns	ns

**D'Agostino & Pearson test**<br><br>**pRab12 / Actin**	**WT-Veh**	**WT-GNE**	**R1441G-Veh**	**R1441G-GNE**
K2	2.145	10.44	0.1858	3.226
P value	0.3421	0.0054	0.9113	0.1993
Passed normality test (alpha=0.05)?	Yes	No	Yes	Yes
P value summary	ns	**	ns	ns

**Unpaired, parametric Student's t-test**<br><br>**pRab12 / Total Rab12**	**R1441G-Veh vs. R1441G-GNE**	**WT-Veh vs. R1441G-Veh**	**WT-Veh vs. WT-GNE**
P value	**0.0008**	**0.0731**	**0.4273**
P value summary	***	**ns**	**ns**
Significantly different? (P < 0.05)	Yes	No	No
One- or two-tailed P value?	Two-tailed	Two-tailed	Two-tailed
t, df	t=3.681 df=33	t=1.852 df=33	t=0.8035 df=34
**F test to compare variances**			
F,DFn, Dfd	1.193, 17, 16	1.714, 17, 16	1.612, 17, 17
P value	0.7281	0.2875	0.3346
P value summary	ns	ns	ns
Significantly different? (P < 0.05)	No	No	No

**Mann-Whitney U-test (unpaired, non-parametric)**<br><br>**pRab12 / Total Rab12**	**R1441G-Veh vs. R1441G-GNE**	**WT-Veh vs. R1441G-Veh**	**WT-Veh vs. WT-GNE**
P value	**0.0012**	**0.0662**	**0.3517**
Exact or approximate P value?	**Exact**	**Exact**	**Exact**
P value summary	**	**ns**	**ns**
Significantly different? (P < 0.05)	Yes	No	No
One- or two-tailed P value?	Two-tailed	Two-tailed	Two-tailed
Sum of ranks in column C,D	401.0 , 229.0	268.0 , 362.0	303.0 , 363.0
Mann-Whitney U	58.00	97.00	132.0

**Unpaired, parametric Student's t-test**<br><br>**pRab12 / Actin**	**R1441G-Veh vs. R1441G-GNE**	**WT-Veh vs. R1441G-Veh**	**WT-Veh vs. WT-GNE**

P value	**0.0006**	**0.0023**	**0.3011**
P value summary	***	**	**ns**
Significantly different? (P < 0.05)	Yes	Yes	No
One- or two-tailed P value?	Two-tailed	Two-tailed	Two-tailed
t, df	t=3.812 df=33	t=3.312 df=33	t=1.050 df=34
**F test to compare variances**			
F,DFn, Dfd	1.189, 17, 16	1.394, 17, 16	1.450, 17, 17
P value	0.7337	0.5110	0.4519
P value summary	ns	ns	ns
Significantly different? (P < 0.05)	No	No	No

**Mann-Whitney U-test (unpaired, non-parametric)**<br><br>**pRab12 / Actin**	**R1441G-Veh vs. R1441G-GNE**	**WT-Veh vs. R1441G-Veh**	**WT-Veh vs. WT-GNE**
P value	**0.0003**	**0.0075**	**0.3357**
Exact or approximate P value?	**Exact**	**Exact**	**Exact**
P value summary	***	**	**ns**
Significantly different? (P < 0.05)	Yes	Yes	No
One- or two-tailed P value?	Two-tailed	Two-tailed	Two-tailed
Sum of ranks in column C,D	410.0 , 220.0	244.0 , 386.0	302.0 , 364.0
Mann-Whitney U	49.00	73.00	131.0

Two-way ANOVA of <u>STRIATUM</u> pRab12 levels in WT and LRRK2^{R1441G} mutant mice after 18-weeks GNE-7915 administration (Fig. 2)

**Two-way ANOVA**	Ordinary				
Alpha	0.05				
Source of Variation	% of total variation	P value	P value summary	Significant?	
**Interaction**	**5.485**	**0.0889**	**ns**	**No**	
**Mutation**	**0.6120**	**0.5646**	**ns**	**No**	
**GNE-7915**	**5.625**	**0.0850**	**ns**	**No**	
ANOVA table	SS (Type III)	DF	MS	F (DFn, DFd)	P value
Interaction	0.04808	1	0.04808	F (1, 48) = 3.016	P=0.0889
WT / R1441G	0.005365	1	0.005365	F (1, 48) = 0.3365	P=0.5646
Vehicle / GNE-7915	0.04931	1	0.04931	F (1, 48) = 3.093	P=0.0850
Residual	0.7653	48	0.01594		

**Two-way ANOVA Analysis**<br>**Tukey's multiple comparisons test**<br><br>**pRab12 / Total Rab12**<br>**(level of phosphorylation)**	**Significant?**	**Summary**	**Adjusted P Value**
**WT:Vehicle vs. WT:GNE-7915**	No	ns	> 0.9999
**WT:Vehicle vs. R1441G:Vehicle**	No	ns	0.8367
WT:Vehicle vs. R1441G:GNE-7915	No	ns	0.3389
WT:GNE-7915 vs. R1441G:Vehicle	No	ns	0.8470
WT:GNE-7915 vs. R1441G:GNE-7915	No	ns	0.3869
**R1441G:Vehicle vs. R1441G:GNE-7915**	No	ns	0.0609

**Two-way ANOVA Analysis**<br>**Tukey's multiple comparisons test**<br><br>**pRab12 / Actin**<br>**(cellular amount of phospho-Rab12)**	**Significant?**	**Summary**	**Adjusted P Value**
**WT:Vehicle vs. WT:GNE-7915**	No	ns	0.9989
**WT:Vehicle vs. R1441G:Vehicle**	No	ns	0.4497
WT:Vehicle vs. R1441G:GNE-7915	No	ns	0.8150
WT:GNE-7915 vs. R1441G:Vehicle	No	ns	0.5787
WT:GNE-7915 vs. R1441G:GNE-7915	No	ns	0.7576
**R1441G:Vehicle vs. R1441G:GNE-7915**	No	ns	0.0871
**Two-way ANOVA Analysis**<br>**Tukey's multiple comparisons test**<br><br>**Total Rab12 / Actin**	**Significant?**	**Summary**	**Adjusted P Value**
**WT:Vehicle vs. WT:GNE-7915**	No	ns	> 0.9999
**WT:Vehicle vs. R1441G:Vehicle**	No	ns	0.8031
WT:Vehicle vs. R1441G:GNE-7915	No	ns	0.9859
WT:GNE-7915 vs. R1441G:Vehicle	No	ns	0.8212
WT:GNE-7915 vs. R1441G:GNE-7915	No	ns	0.9871
**R1441G:Vehicle vs. R1441G:GNE-7915**	No	ns	0.9405

Single factor analysis:

**D'Agostino & Pearson test**<br><br>**pRab12 / Total Rab12**	**WT-Veh**	**WT-GNE**	**R1441G-Veh**	**R1441G-GNE**
K2	0.3884	1.596	0.3434	2.325
P value	0.8235	0.4502	0.8422	0.3127
Passed normality test (alpha=0.05)?	Yes	Yes	Yes	Yes
P value summary	ns	ns	ns	ns

**D'Agostino & Pearson test**<br><br>**pRab12 / Actin**	**WT-Veh**	**WT-GNE**	**R1441G-Veh**	**R1441G-GNE**
K2	1.036	1.920	2.703	1.210
P value	0.5956	0.3829	0.2588	0.5462
Passed normality test (alpha=0.05)?	Yes	Yes	Yes	Yes
P value summary	ns	ns	ns	ns

**Unpaired, parametric Student's t-test**<br><br>**pRab12 / Total Rab12**	**R1441G-Veh vs. R1441G-GNE**	**WT-Veh vs. R1441G-Veh**	**WT-Veh vs. WT-GNE**
P value	**0.0168**	**0.3502**	**0.9880**
P value summary	*	**ns**	**ns**
Significantly different? (P < 0.05)	Yes	No	No
One- or two-tailed P value?	Two-tailed	Two-tailed	Two-tailed
t, df	t=2.556 df=26	t=0.9521 df=25	t=0.01516 df=22
**F test to compare variances**			

F,DFn, Dfd	1.161, 13, 13	2.565, 13, 12	3.863, 10, 12
P value	0.7924	0.1128	0.0303
P value summary	ns	ns	*
Significantly different? (P < 0.05)	No	No	Yes

**Mann-Whitney U-test (unpaired, non-parametric)**<br><br>**pRab12 / Total Rab12**	**R1441G-Veh vs. R1441G-GNE**	**WT-Veh vs. R1441G-Veh**	**WT-Veh vs. WT-GNE**
P value	**0.0162**	**0.2763**	**0.5594**
Exact or approximate P value?	**Exact**	**Exact**	**Exact**
P value summary	*****	**ns**	**ns**
Significantly different? (P < 0.05)	Yes	No	No
One- or two-tailed P value?	Two-tailed	Two-tailed	Two-tailed
Sum of ranks in column C,D	255.0 , 151.0	159.0 , 219.0	173.0 , 127.0
Mann-Whitney U	46.00	68.00	61.00

**Unpaired, parametric Student's t-test**<br><br>**pRab12 / Actin**	**R1441G-Veh vs. R1441G-GNE**	**WT-Veh vs. R1441G-Veh**	**WT-Veh vs. WT-GNE**
P value	**0.0358**	**0.1489**	**0.8724**
P value summary	*****	**ns**	**ns**
Significantly different? (P < 0.05)	Yes	No	No
One- or two-tailed P value?	Two-tailed	Two-tailed	Two-tailed
t, df	t=2.214 df=26	t=1.489 df=25	t=0.1625 df=22
**F test to compare variances**			
F,DFn, Dfd	1.828, 13, 13	3.663, 13, 12	2.829, 10, 12
P value	0.2895	0.0314	0.0915
P value summary	ns	*	ns
Significantly different? (P < 0.05)	No	Yes	No

**Mann-Whitney U-test (unpaired, non-parametric)**<br><br>**pRab12 / Actin**	**R1441G-Veh vs. R1441G-GNE**	**WT-Veh vs. R1441G-Veh**	**WT-Veh vs. WT-GNE**
P value	**0.0495**	**0.3962**	**0.8766**
Exact or approximate P value?	**Exact**	**Exact**	**Exact**
P value summary	*****	**ns**	**ns**
Significantly different? (P < 0.05)	Yes	No	No
One- or two-tailed P value?	Two-tailed	Two-tailed	Two-tailed
Sum of ranks in column C,D	246.0 , 160.0	164.0 , 214.0	159.5 , 140.5
Mann-Whitney U	55.00	73.00	68.50

Two-way ANOVA of LUNG pRab10 levels in WT and LRRK2^{R1441G} mutant mice after 18-weeks GNE-7915 administration (Fig. 2)

**Two-way ANOVA**	Ordinary				
Alpha	0.05				
Source of Variation	% of total variation	P value	P value summary	Significant?	
**Interaction**	**13.11**	**0.0003**	***	**Yes**	
**Mutation**	**7.446**	**0.0050**	**	**Yes**	
**GNE-7915**	**19.25**	**< 0.0001**	****	**Yes**	
ANOVA table	SS (Type III)	DF	MS	F (DFn, DFd)	P value
Interaction	1.596	1	1.596	F (1, 66) = 14.83	P = 0.0003
WT / R1441G	0.9065	1	0.9065	F (1, 66) = 8.421	P = 0.0050
Vehicle / GNE-7915	2.344	1	2.344	F (1, 66) = 21.78	P < 0.0001
Residual	7.104	66	0.1076		

**Two-way ANOVA Analysis<br>Tukey's multiple comparisons test<br><br>pRab10 / Total Rab10<br>(level of phosphorylation)**	**Significant?**	**Summary**	**Adjusted P Value**
**WT:Vehicle vs. WT:GNE-7915**	No	ns	0.9452
**WT:Vehicle vs. R1441G:Vehicle**	Yes	****	< 0.0001
WT:Vehicle vs. R1441G:GNE-7915	No	ns	0.5895
WT:GNE-7915 vs. R1441G:Vehicle	Yes	****	< 0.0001
WT:GNE-7915 vs. R1441G:GNE-7915	No	ns	0.9046
**R1441G:Vehicle vs. R1441G:GNE-7915**	Yes	****	< 0.0001
**Two-way ANOVA Analysis<br>Tukey's multiple comparisons test<br><br>pRab10 / Actin<br>(cellular amount of phospho-Rab10)**	**Significant?**	**Summary**	**Adjusted P Value**
**WT:Vehicle vs. WT:GNE-7915**	No	ns	0.9936
**WT:Vehicle vs. R1441G:Vehicle**	Yes	****	< 0.0001
WT:Vehicle vs. R1441G:GNE-7915	No	ns	0.8341
WT:GNE-7915 vs. R1441G:Vehicle	Yes	****	< 0.0001
WT:GNE-7915 vs. R1441G:GNE-7915	No	ns	0.9419
**R1441G:Vehicle vs. R1441G:GNE-7915**	Yes	****	< 0.0001
**Two-way ANOVA Analysis<br>Tukey's multiple comparisons test<br><br>Total Rab10 / Actin**	**Significant?**	**Summary**	**Adjusted P Value**
**WT:Vehicle vs. WT:GNE-7915**	No	ns	0.8787
**WT:Vehicle vs. R1441G:Vehicle**	No	ns	0.7228
WT:Vehicle vs. R1441G:GNE-7915	No	ns	0.5264
WT:GNE-7915 vs. R1441G:Vehicle	No	ns	0.9922
WT:GNE-7915 vs. R1441G:GNE-7915	No	ns	0.9380
**R1441G:Vehicle vs. R1441G:GNE-7915**	No	ns	0.9904

Two-way ANOVA of LUNG pRab12 levels in WT and LRRK2^{R1441G} mutant mice after 18-weeks GNE-7915 administration (Fig. 2)

**Two-way ANOVA**	Ordinary				
Alpha	0.05				
Source of Variation	% of total variation	P value	P value summary	Significant?	
**Interaction**	**8.483**	**0.0061**	**	**Yes**	
**Mutation**	**1.533**	**0.2303**	**ns**	**No**	
**GNE-7915**	**29.43**	**< 0.0001**	****	**Yes**	
ANOVA table	SS (Type III)	DF	MS	F (DFn, DFd)	P value
Interaction	0.08419	1	0.08419	F (1, 56) = 8.137	P = 0.0061
WT / R1441G	0.01522	1	0.01522	F (1, 56) = 1.471	P = 0.2303
Vehicle / GNE-7915	0.2921	1	0.2921	F (1, 56) = 28.23	P < 0.0001
Residual	0.5794	56	0.01035		

**Two-way ANOVA Analysis<br>Tukey's multiple comparisons test<br><br>pRab12 / Total Rab12<br>(level of phosphorylation)**	**Significant?**	**Summary**	**Adjusted P Value**
**WT:Vehicle vs. WT:GNE-7915**	No	ns	0.3423
**WT:Vehicle vs. R1441G:Vehicle**	Yes	*	0.0245
WT:Vehicle vs. R1441G:GNE-7915	Yes	*	0.0229
WT:GNE-7915 vs. R1441G:Vehicle	Yes	***	0.0002
WT:GNE-7915 vs. R1441G:GNE-7915	No	ns	0.6678
**R1441G:Vehicle vs. R1441G:GNE-7915**	Yes	****	< 0.0001
**Two-way ANOVA Analysis<br>Tukey's multiple comparisons test<br><br>pRab12 / Actin<br>(cellular amount of phospho-Rab12)**	**Significant?**	**Summary**	**Adjusted P Value**
**WT:Vehicle vs. WT:GNE-7915**	No	ns	0.9979
**WT:Vehicle vs. R1441G:Vehicle**	No	ns	0.0606
WT:Vehicle vs. R1441G:GNE-7915	No	ns	0.3386
WT:GNE-7915 vs. R1441G:Vehicle	Yes	*	0.0491
WT:GNE-7915 vs. R1441G:GNE-7915	No	ns	0.4787
**R1441G:Vehicle vs. R1441G:GNE-7915**	Yes	***	0.0004
**Two-way ANOVA Analysis<br>Tukey's multiple comparisons test<br><br>Total Rab12 / Actin**	**Significant?**	**Summary**	**Adjusted P Value**
**WT:Vehicle vs. WT:GNE-7915**	No	ns	0.7627
**WT:Vehicle vs. R1441G:Vehicle**	No	ns	0.9801
WT:Vehicle vs. R1441G:GNE-7915	No	ns	0.9927
WT:GNE-7915 vs. R1441G:Vehicle	No	ns	0.9254
WT:GNE-7915 vs. R1441G:GNE-7915	No	ns	0.8853
**R1441G:Vehicle vs. R1441G:GNE-7915**	No	ns	0.9994

Supplementary Table S2:

Histopathology of kidney after chronic 18-week GNE-7915 treatment (Score: 0=Normal; 1=Mild; 2=Moderate; 3=Severe). For levels of pathological severity of vacuolation: No vacuolation (0 – normal), <25% of the cortical tubules have vacuoles (1 – mild), 25–50% cortical have vacuoles (2 – moderate), >50% cortical tubules have vacuoles (3 – severe); For tubulointerstitial injuries: No observable injuries (0 – normal), <10% of the cortex (1 – mild), 10–50% of the cortex (2 - moderate), >50% of the cortex (3 – severe). For proliferative glomerulus, at least 10-20 glomeruli were examined. No proliferation glomerulonephritis (0 – normal), <25% total glomerulus involved (1 – mild),<50% involvement (2 – moderate), >50% involvement (3 – severe).

Mouse genotype (Male)		Observation	Vacuolation (outer cortex)	Proliferative glomerulus	*Tubulointerstitial injury
**Untreated age-matched normal**					
WT untreated-1		No vacuolation, some tubular injury, some proliferative glomeruli	-	+	+
WT untreated-2		Normal, No vacuoles	-	-	-
WT untreated-3		Normal, no vacuoles	-	-	-
WT untreated-4		Normal glomeruli, no vacuoles, some tubular injury	-	-	+
WT untreated-5		Normal, no vacuoles	-	-	-
R1441G untreated-1		No vacuoles or tubular atrophy, Bowman's capsule (in some) are thicker	-	-	-
R1441G untreated-2		Normal, no vacuoles	-	-	-
R1441G untreated-3		No vacuoles, but tubules look 'jagged', tubular injury	-	-	+
R1441G untreated-4		Normal, no vacuoles	-	-	-
R1441G untreated-5		No Vacuoles, proliferative glomeruli, tubular injury	-	-	-
**Vehicle [40% (w/v) (2-hydroxypropyl)-*β*-cyclodextrin]**					
WT vehicle-1		Vacuoles, tubular injury	+	-	+
WT vehicle-2		Vacuoles present in tubules, glomeruli normal	+	-	-
WT vehicle-3		Vacuoles, proliferative glomeruli, tubular injury, dilation on some tubules	+	+	+
WT vehicle-4		Vacuoles, glomeruli proliferative, tubular injury	+	+	+
WT vehicle-5		Moderate vacuoles, some glomeruli proliferative	++	+	-
WT vehicle-6		Glomeruli normal, vacuoles,	+	-	-
WT vehicle-7		Glomeruli normal, vacuoles, infiltrating cells at tubules	+	-	-
WT vehicle-8		tubular dilation	+	-	+
WT vehicle-9		small regional vacuolation	+	-	-
WT vehicle-10		small regional vacuolation; normal kidney in general	+	-	-

WT vehicle-11		Vacuoles	++	-	-
WT vehicle-12		Vacuoles	+	-	-
WT vehicle-13		Immune cell infiltration	++	-	+
WT vehicle-14		Vacuoles	++	-	-
WT vehicle-15		Vacuoles	++	-	-
WT vehicle-16		Some tubular atrophy & dilation	+	-	+
R1441G vehicle-1		Vacuoles	+	-	-
R1441G vehicle-2		Vacuoles (moderate)	++	-	-
R1441G vehicle-3		Vacuoles, some glomeruli proliferative	+	+	-
R1441G vehicle-4		Vacuoles	+	-	-
R1441G vehicle-5		Vacuoles, some proliferative glomeruli	+	+	-
R1441G vehicle-6		Moderate vacuoles, some glomeruli proliferative	++	+	-
R1441G vehicle-7		Minor vacuolation, infiltrate of immune cells, proliferative glomeruli	+	+	-
R1441G vehicle-8		Moderate vacuolation, tubulo-interstitial, inflammation, some glomeruli proliferative	++	+	+
R1441G vehicle-9		Normal glomeruli, no vacuoles, some tubular injury	-	-	+
R1441G vehicle-10		Enlarged & proliferative glomeruli, minor vacuolation	+	+	-
R1441G vehicle-11		Glomeruli normal, infiltrating cells at tubules	-	-	-
R1441G vehicle-12		Tubular dilation	-	-	+
R1441G vehicle-13		Minor vacuolation	+	-	-
R1441G vehicle-14		Vacuoles	+	-	-
R1441G vehicle-15		Vacuoles	+	-	-
R1441G vehicle-16		Tubular dilation	-	-	+
**GNE-7915 (100mg/kg; twice weekly; 18 weeks; s.c.)**					
WT GNE-1		Vacuoles, some glomeruli proliferative	+	+	-
WT GNE-2		Vacuoles	+	-	-
WT GNE-3		Vacuoles (moderate)	++	-	-
WT GNE-4		Intense vacuoles, tubular atrophy	+++	-	+
WT GNE-5		Vacuoles, some glomeruli proliferative	+	+	-
WT GNE-6		Vacuoles, glomeruli proliferative, tubular injury	+	+	+
WT GNE-7		Vacuoles, glomeruli proliferative	+	+	-
WT GNE-8		Some tubular atrophy, vacuoles, normal glomeruli	+	-	+
WT GNE-9		Vacuoles, glomeruli proliferative, inflammatory cell infiltration	+	+	+
WT GNE-10		Vacuoles	++	-	-
WT GNE-11		Vacuoles	++	-	-

WT GNE-12		Some mesangial expansion	+	-	+
WT GNE-13		Some immune cell infiltration	+	-	+
WT GNE-14		Vacuoles	++	-	-
WT GNE-15		Vacuoles	++	-	-
WT GNE-16		Vacuoles	++	-	-
WT GNE-17		Minor tubular dilation	+	-	+
WT GNE-18		Some tubular dilation	-	-	+
WT GNE-19		Some tubular atrophy & dilation	-	-	+
R1441G GNE-1		Vacuoles (moderate)	++	-	-
R1441G GNE-2		Vacuoles, some tubular atrophy	+	-	+
R1441G GNE-3		Vacuolation, proliferative glomeruli, tubulo-interstitial, inflammation	+	+	+
R1441G GNE-4		Vacuoles (moderate)	++	-	-
R1441G GNE-5		Glomeruli proliferative, vacuoles	+	+	-
R1441G GNE-6		Moderate vacuoles, some glomeruli proliferative	++	+	-
R1441G GNE-7		Moderate vacuoles, glomeruli proliferative	++	+	-
R1441G GNE-8		Some vacuolation, some proliferative glomeruli	+	+	-
R1441G GNE-9		Vacuolation, proliferative glomeruli, tubulo-interstitial, inflammation, tubular dilation	+	+	+
R1441G GNE-10		Vacuoles, proliferative glomeruli, tubular injury	+	+	+
R1441G GNE-11		Glomeruli normal, vacuoles	+	-	-
R1441G GNE-12		Glomeruli normal, vacuoles	+	-	-
R1441G GNE-13		Glomeruli normal, vacuoles	+	-	-
R1441G GNE-14		proliferative glomeruli, vacuoles (moderate)	++	+	-
R1441G GNE-15		Tubular dilation	+	-	+
R1441G GNE-16		Vacuoles	+	-	-
R1441G GNE-17		Minor vacuolation; Tubular dilation; Some mesangial expansion	+	+	-

Original Western blot scans

Fig. 1a

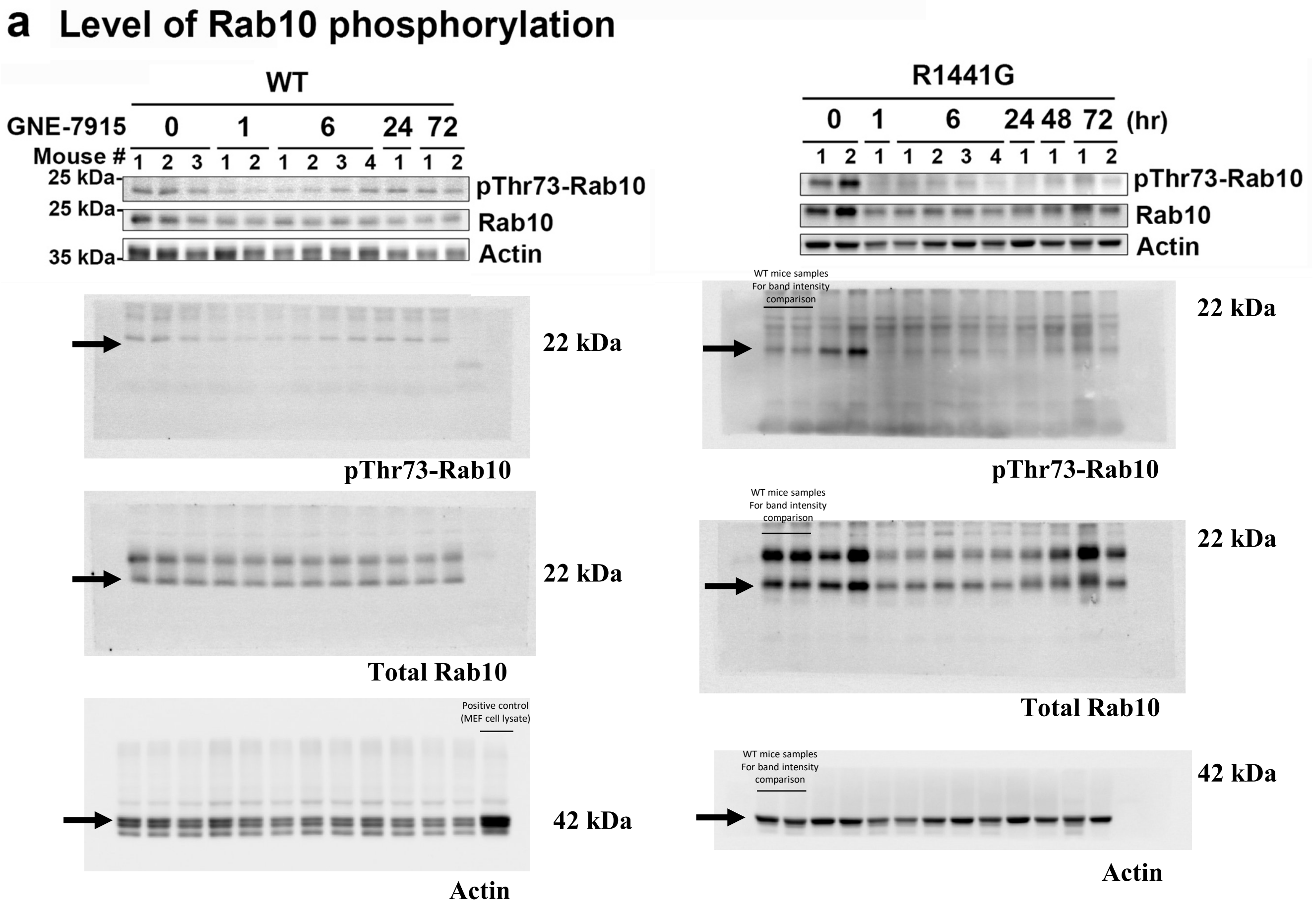

Fig. 1c

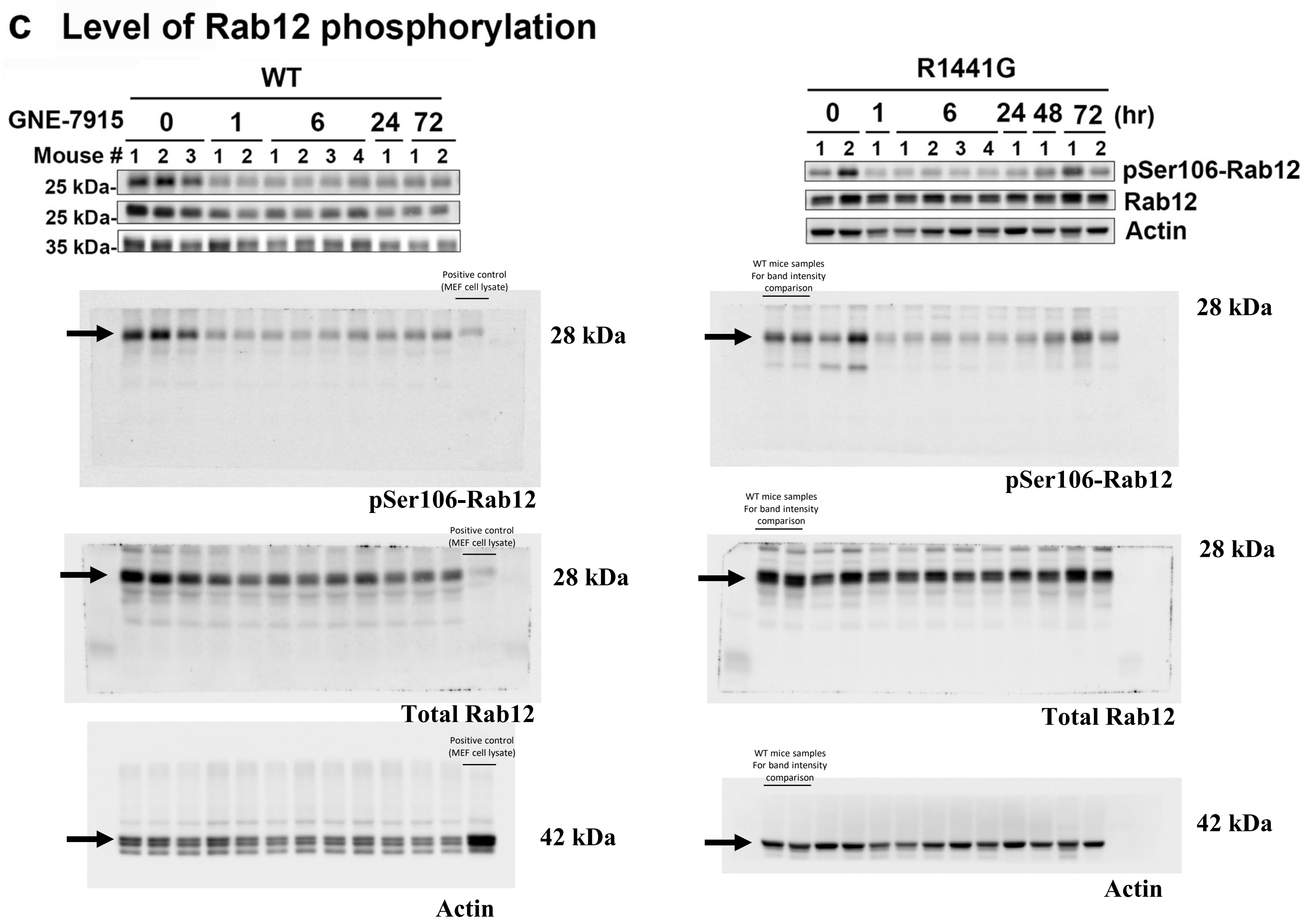

Fig. 2a

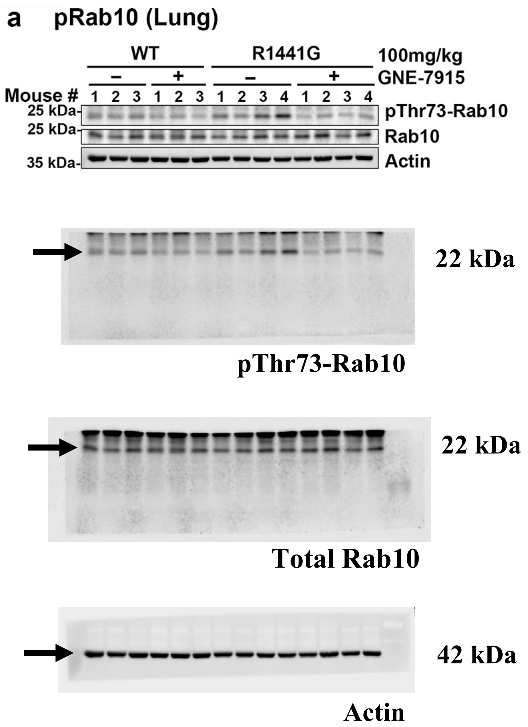

Fig. 2b

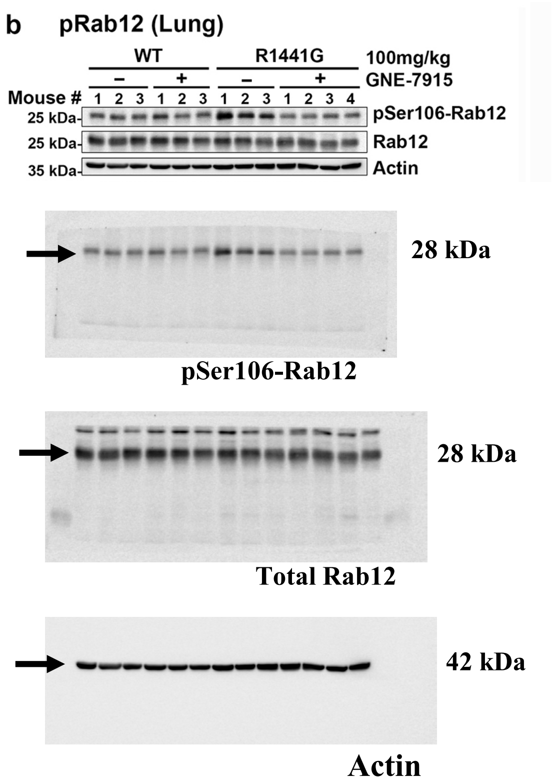

Fig. 2i

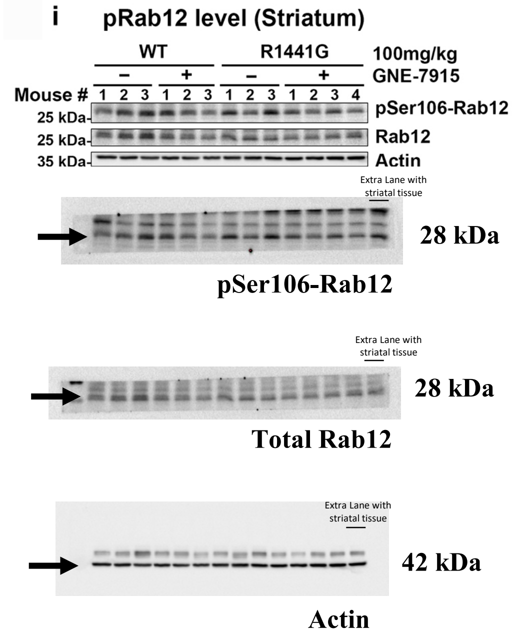

Fig. 2j

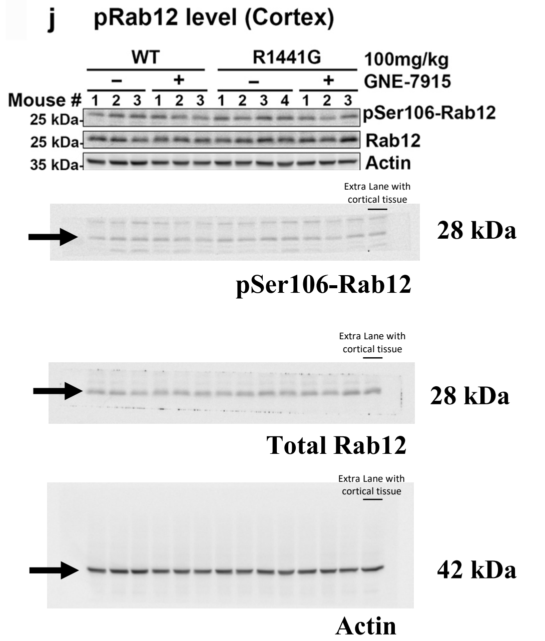

Fig. 4c

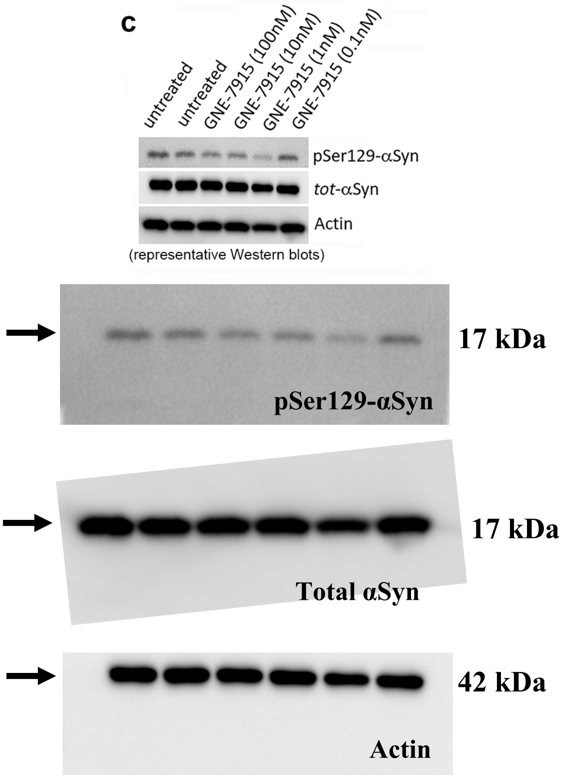

Fig. 4b

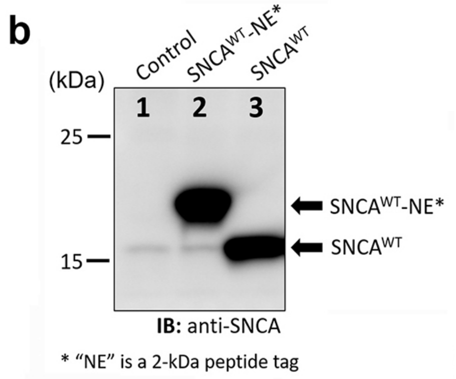

Fig. 4g

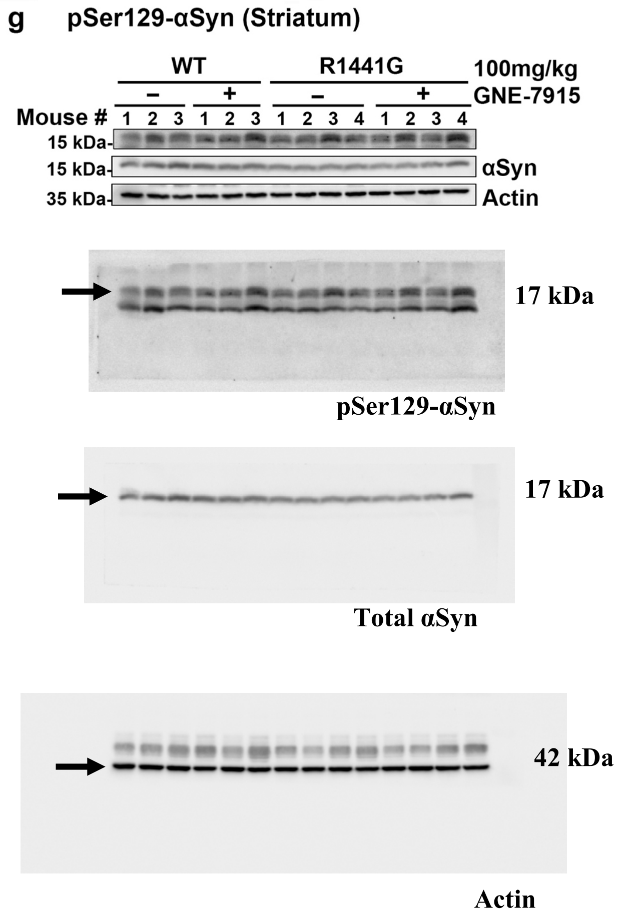

Fig. 4h

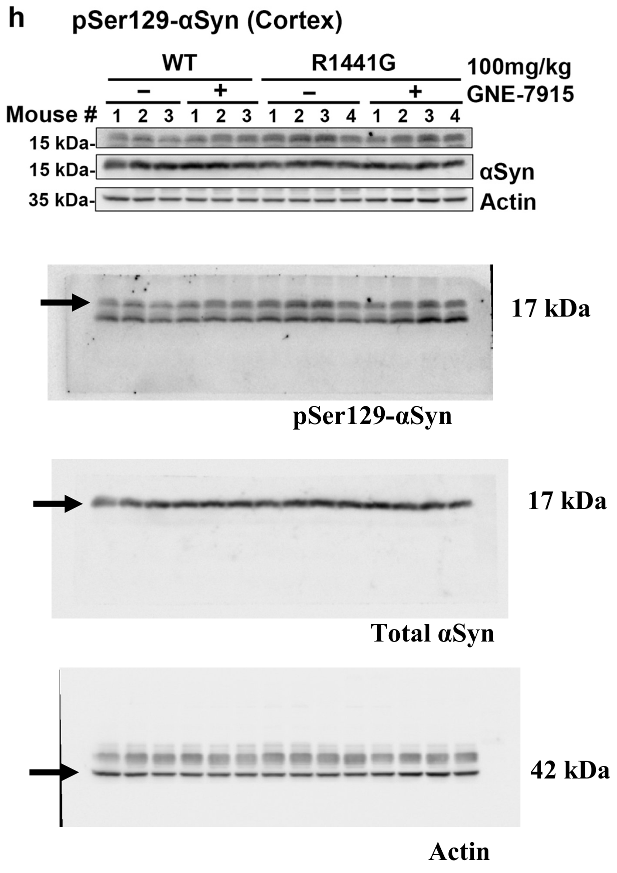

Supplement: Supplementary file 1 — Supplementary Information [file 41531_2022_386_MOESM1_ESM.pdf]
